# Histone acetylation facilitates multidirectional pulp repair through Neuregulin-1 mobilization

**DOI:** 10.1093/stcltm/szaf022

**Published:** 2025-06-28

**Authors:** Zhiwu Wu, Hui Yang, Shaoying Duan, Qianqian Su, Ran Cheng, Tao Hu

**Affiliations:** State Key Laboratory of Oral Diseases & National Center for Stomatology & National Clinical Research Center for Oral Diseases & Frontier Innovation Center for Dental Medicine Plus, West China Hospital of Stomatology, Sichuan University, Chengdu 610041, People’s Republic of China; School of Stomatology, Zhejiang Chinese Medical University, The Stomatology Hospital of Zhejiang Chinese Medical University, Hangzhou 310053, People’s Republic of China; State Key Laboratory of Oral Diseases & National Center for Stomatology & National Clinical Research Center for Oral Diseases & Frontier Innovation Center for Dental Medicine Plus, West China Hospital of Stomatology, Sichuan University, Chengdu 610041, People’s Republic of China; State Key Laboratory of Oral Diseases & National Center for Stomatology & National Clinical Research Center for Oral Diseases & Frontier Innovation Center for Dental Medicine Plus, West China Hospital of Stomatology, Sichuan University, Chengdu 610041, People’s Republic of China; State Key Laboratory of Oral Diseases & National Center for Stomatology & National Clinical Research Center for Oral Diseases & Frontier Innovation Center for Dental Medicine Plus, West China Hospital of Stomatology, Sichuan University, Chengdu 610041, People’s Republic of China; State Key Laboratory of Oral Diseases & National Center for Stomatology & National Clinical Research Center for Oral Diseases & Frontier Innovation Center for Dental Medicine Plus, West China Hospital of Stomatology, Sichuan University, Chengdu 610041, People’s Republic of China; State Key Laboratory of Oral Diseases & National Center for Stomatology & National Clinical Research Center for Oral Diseases & Frontier Innovation Center for Dental Medicine Plus, West China Hospital of Stomatology, Sichuan University, Chengdu 610041, People’s Republic of China

**Keywords:** H3K9ac, H3K27ac, NRG1, pulp regeneration, SAHA

## Abstract

Appropriate dental pulp repair is based on effective control of inflammation and involves the regeneration of dental pulp nerves, blood vessels (soft tissue), and dentin (hard tissue). Limited evidence has shown how to modulate the uncertainty due to individual variability in dental pulp repair. NRG1, a cytokine modulating nerve injury and repair, was intricately associated with the outcome of pulp repair. Yet, its mobilization in spontaneous pulp repair had individual variability. The study further explored the role of NRG1 during pulp repair as well as an epigenetic way to modulate NRG1 through histone acetylation to enhance pulp repair. Overexpression of NRG1 exhibited the effects of anti-inflammation and integrated regeneration of soft and hard tissue, by inhibiting pro-inflammatory factors IL-1β, IL-8, and promoting the expressions of DSPP, DMP1 (dentin regeneration), and nestin (nerve regeneration). Moreover, restricted H3K9 and H3K27 acetylation correlated with NRG1 expression in pulp repair both temporally and spatially, showing individual variability as well. Suberoylanilide hydroxamic acid (SAHA), a histone deacetylase (HDAC) inhibitor, enhanced H3K9ac and H3K27ac, which dramatically activated NRG1, suppressed pulp inflammation, and facilitated soft and hard tissue regeneration. In summary, targeting histone acetylation with HDAC inhibitors may be an effective approach to promote pulp repair by activating NRG1.

## Graphical Abstract

**Figure:**
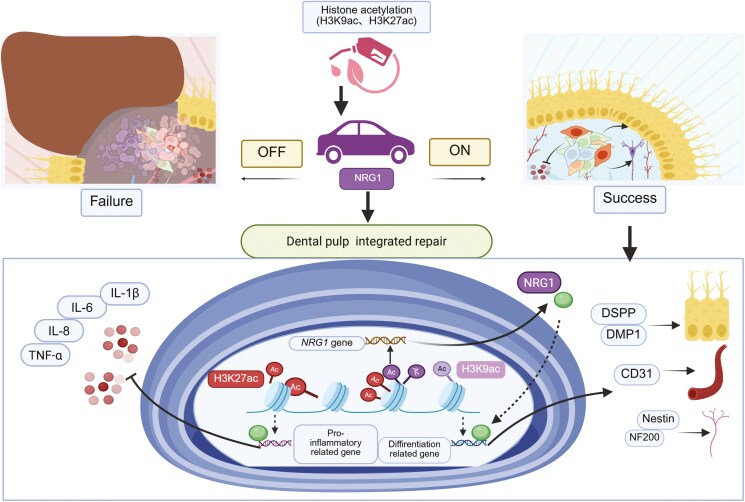
Enhanced histone acetylation facilitated pulp repair through NRG1 mobilization via anti-inflammatory and pro-regenerative mechanisms. NRG1 driven by enhanced histone acetylation, started the journey of dental pulp repair. NRG1 promoted pulp regeneration by inhibiting proinflammatory factors and enhanced dentin and nerve regenerative capacity.

**Significance statement**

Appropriate pulp restoration is currently the focus of attention, and there are uncertainties. Our study showed that NRG1 was related to the outcome of pulp repair, and NRG1 inhibited pulp inflammation and promoted dentin and nerve regeneration. Enhanced histone acetylation can promote NRG1 and realize the function of NRG1 in pulp repair. Our results suggested that enhancing NRG1 through enhanced histone acetylation was a promising option to promote pulp repair.

## Introduction

Appropriate repair of dental pulp injury is evident in effective control of inflammation, the regeneration of dental pulp nerves, blood vessels (soft tissue), and dentin (hard tissue). Dental pulp stem cells (DPSCs) are acknowledged as pivotal reparative seeds in inflammatory regulation, dentin regeneration, neurogenesis, and angiogenesis.^1-4^ However, pulp regeneration and repair are vital clinical challenges.^5^ Clinical findings suggest that the outcome of dental pulp regeneration under both physiological and pathological conditions is ambiguous, with the success rates directly linked to individual variables such as patient age and the severity of injury.^6,7^ Treatments such as vital pulpotomy and revascularization are mostly applicable to immature permanent teeth.^8,9^

Despite the above-mentioned factors, there exists significant uncertainty concerning the outcomes of dental pulp repair. Previous research has predominantly concentrated on the activated or highly expressed factors during pulp repair and hard tissue regeneration.^10-12^ However, some key factors involved in the pulp repair may remain in an “OFF” state, causing DPSCs to be relatively quiescent^13-15^ and resulting in failures in pulp repair remain to be elucidated. Activating these factors might potentially mobilize anti-inflammation, integrated soft and hard tissue regeneration in the dental pulp.

What could activate such quiescent DSPCs? Multi-functional neural repair factor has become our first choice. Neuregulin-1 (NRG1), a member of the epidermal growth factor family, is extensively present in central and peripheral nerves and modulates nerve injury and repair.^16^ NRG1 in unmyelinated neurons is essential for myelinogenesis in nearby interneurons.^17^ It was reported that NRG1 overexpression enhances the proliferation and migration of DPSCs and facilitates their neurogenic differentiation, thereby aiding in facial nerve regeneration.^18^ In addition, NRG1 promotes soft tissue regeneration such as intestinal crypts,^19^ and engages in the osteogenic differentiation of stem cells to modulate bone healing.^20^ NRG1 has been implicated in the regulation of inflammation by reducing ROS production and inhibiting the NLRP3/caspase-1 pathway, thereby attenuating myocardial oxidative damage and inflammatory responses.^21^ NRG1 increases the differential potential of stem cells, inhibits inflammation, and promotes soft and hard tissue regeneration. NRG1 may serve as a crucial factor in light of the uncertainty of the regenerative effect during pulp regeneration.

When NRG1 is determined, how can it be regulated? Prevalent targeted interventions encompass gene editing, epigenetic modification,etc.. Unlike gene editing, epigenetic modification does not involve any alteration to the DNA sequence,^22^ and it has lately emerged as a therapy for numerous diseases.^23^ There are many types of epigenetic modifications, including DNA methylation, histone methylation, histone acetylation, non-coding RNAs, etc.^24^ Histone acetylation is possibly a major way to mobilize NRG1 by its interaction with *NRG1* promoter locus.^25^ Whether histone acetylation are involved in pulp regeneration and their roles deserves further investigation.

Therefore, this study aimed to explore whether NRG1 is a potential target during pulp regeneration, as well as the safer and more effective way—whether NRG1 can be modulated epigenetically through histone acetylation to enhance pulp regeneration.

## Materials and methods

All commercial antibodies (Supplementary Table S1) and some methods used in this study were refer to Supplemental materials.

### Data collection and analysis of targets related to pulp regeneration by Genecards and UniproKB

We examined the Genecards database for genes associated with pulp regeneration (PR), neurogenic differentiation (ND), angiogenic differentiation (AD), osteogenic differentiation (OsD), and odontogenic differentiation (OdD), respectively. The functions of the gene/protein were collected by UniproKB.

### Establishment of pulp injury model and spontaneous mineralization model in rats

The experimental rats were obtained from Beijing HFK Bioscience Co., Ltd. and were 7-week-old SD male rats weighing about 220 g. The rats were anesthetized with isoflurane by inhalation, and the pulp was opened in the mesial half of the occlusal surface of the upper first molar. Subsequently, 2 μL(10 mg/mL) of *Escherichia coli (E. coli* LPS (297-473-0, Sigma) was slowly injected into the pulp cavity.^26^ Cavities were immediately filled with glass ionomer cement. Rats in the control group were untreated. The rats were sacrificed by CO_2_ asphyxiation, and rat maxillae were collected from the control group, 3 hours, 12 hours, 1 day, 3 days, and 7 days after pulp exposure, respectively.

To establish a spontaneous mineralization model, iRoot BP Plus was used on the basis of the pulp injury model for 7 and 28 days.

### Data collection and analysis of RNA-seqencing (RNA-seq) by GEO database

The RNA-seq dataset (GSE198359) was selected in GEO database by searching for the two keywords “pulpitis” and “caries” to verification of NRG1 in pulp tissue from normal, carious, and pulpitis teeth.

### Culture of human DPSCs (hDPSCs)

The hDPSCs were seeded at a density of 1 × 10^6^ cells per well in 6-well plates in this study. The cells were stimulated with 1 μg/mL LPS to create a model of pulp injury *in vitro*.^27^ A mineralization-inducing medium (MIM), comprising complete medium supplemented with 50 mg/L Vitamin C, 10 nM sodium β-glycerophosphate, and 10 mM dexamethasone, was employed to induce OdD of hDPSCs, thereby establishing a spontaneous mineralization model *in vitro*. Histone acetylation intervention was conducted via 1 μM SAHA (S1047, Selleck, USA) and 10 μM C646 (S7152, Selleck) based on pulp injury and OdD model *in vitro*.

### Long non-coding RNA sequencing (lncRNA-seq) and the assay for transposase-accessible chromatin using sequencing (ATAC-seq)

hDPSCs were harvested at 1, 3, 7, and 14 days after establishment of pulp injury and spontaneous mineralization model *in vitro* for lncRNA-seq and ATAC-seq. More details can be found in the Supplemental Materials.

### Lentivirus-mediated *NRG1* knockdown/overexpression

hDPSCs were infected by lentivirus under optimum conditions selected by pre-experiment to obtain cell lines capable of stably knocking down/overexpressing *NRG1*. More details can be found in the Supplemental Materials.

The hDPSCs with *NRG1* knockdown/overexpression were stimulated with 1 μg/mL LPS for 24 hours. hDPSCs were harvested for Western blot. Additionally, hDPSCs were cultured in MIM to create a mineralization model for *NRG1* intervention *in vitro*. hDPSCs were harvested for alkaline phosphatase (ALP), Western blot, and mineralized nodule detection.

### Subcutaneous transplantation in nude mice

4-week-old BALB/c-nu thymus-free nude mice were purchased from Beijing HFK Bioscience Co, Ltd, 4 weeks old, weighing about 15 g. Using isoflurane inhalation anesthesia and routine sterilization, hDPSCs with *NRG1* knockdown/overexpression were mixed with Matrigel (1.0 × 10^6^ cells/10 μL, 354248, Corning, USA) and seeded into the human-treated dentin matrix (TDM, the preparation of TDM could be found in the Supplemental materials) and then subcutaneously transplanted into nude mice for 4 weeks to observe pulp-dentin regeneration. In addition, 1 μg/mL LPS was added to a mixture of hDPSCs with *NRG1* knockdown/overexpression and Matrigel to establish pulp injury *in vivo*.

### Pulp injury and mineralization model of histone acetylation intervention in rats

Following the establishment of the pulp injury and mineralization model in rats, a gelatin sponge was immersed in 2 μL of DMSO/1 μM SAHA/10 μM C646 and placed in the exposed pulp cavity. Subsequently, glass ionomer cement was filled in the pulp cavity while the vehicle (solvent)/SAHA (50 mg/kg/day)^28^/C646 (10 mg/kg/day)^29^ was administered intraperitoneally. The solvent comprised 5% DMSO, 40% PEG300, 5% Tween80, and 50% sterilized ddH_2_O. The rats were euthanized via CO_2_ asphyxiation, and the maxillae were separated at 3, 7, and 28 days.

### Chromatin immunoprecipitation followed by quantitative polymerase chain reaction (ChIP-qPCR)

This experiment was conducted in accordance with the SimpleChIP Chromatin Immunoprecipitation Procedure (9003, Cell Signaling Technology). It encompasses cell culture crosslinking and sample preparation, nuclear preparation, chromatin digestion and concentration analysis, chromatin immunoprecipitation, chromatin elution from antibody/magnetic beads and de-crosslinking, DNA purification, qPCR quantification of DNA, and subsequent analysis. Forward primer: CCCTGCTTGTATCTCTGCTCTTTG, Revese primer: ATGTGCGAGGTCTCTTGAGGTC.

### Statistical analysis

All data in this study were analyzed and plotted using GraphPad Prism 8.0, with findings expressed as mean ± SD. A one-way ANOVA was employed to compare means among various groups, while a T-test was utilized for comparisons between two groups. A *P*-value of < .05 was deemed statistically significant.

## Results

### NRG1 was intricately associated with pulp repair; yet, its mobilization in spontaneous pulp repair posed challenges

**Figure 1.**
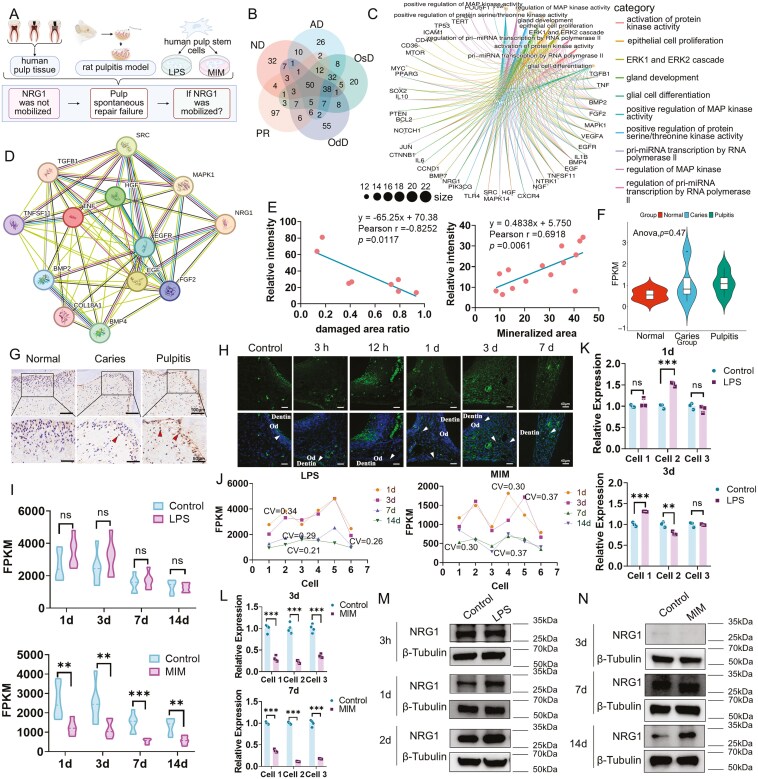
NRG1 was significantly linked to pulp repair but exhibited low expression during pulp inflammation and spontaneous mineralization. (A) The schematic diagram of NRG1 in pulp spontaneous repair (pulp inflammation and spontaneous mineralization). (B)Venn plots of genes associated with pulp regeneration (PR), neurogenic differentiation (ND), vasculogenic differentiation (VD), and osteogenic/odontogenic differentiation (OsD/OdD) were taken from the Genecards database. The diagram was acquired by Bioinformatics platform (https://www.bioinformatics.com.cn). (C) GO pathway enrichment analysis of 50 genes identified in A. The diagram was acquired by Bioinformatics platform (https://www.bioinformatics.com.cn). (D) PPI of 13 genes participating in over 5 biological processes. The diagram was acquired by String(https://www.string-db.org/). (E) Correlation between NRG1 expression and inflammation and mineralization in rat dental pulp. (F) Bioinformatics analysis of human dental pulp. The data was from GEO database (GSE198359). The diagram was acquired by Bioinformatics platform (https://www.bioinformatics.com.cn). (G) Expression of NRG1 in human dental pulp by IHC staining. *N* = 4. (H) Expression of NRG1 in a rat model of pulpitis by IF staining. Od, odontoblast layer. *N* = 4. Bar, 40 μm. (I, J). Bioinformatics analysis of inflammation (LPS) and mineralization (MIM) model of hDPSCs *in vitro*. (K) Validation of *NRG1* gene expression in inflammation (LPS) model of hDPSCs *in vitro* by qPCR, *N* = 3. (L) Validation of *NRG1* gene expression in mineralization (MIM) model of hDPSCs *in vitro* by qPCR, *N* = 4. (M) Expression of NRG1 in hDPSCs during inflammation *in vitro* by Western blot, *N* = 3. (*N*) Expression of NRG1 in hDPSCs in mineralization model *in vitro* by Western blot, *N* = 3. Ns, no significance, **P < .01,***P < .001.

Pulp repair encompasses dentin formation, nerve regeneration, and vascular regeneration. Upon choosing the top 200 genes from each set for intersection (PR, ND, AD, OsD, OdD), 50 genes were identified as being linked with all five dimensions (Figure 1B). 50 screened genes underwent GO pathway enrichment analysis, revealing that 13 genes were implicated in more than five of the top 10 biological pathways (Figure 1C). Ultimately, we investigated the functions of these genes using UniproKB (Supplementary Table S2) and searched the literatures related to pulp regeneration. Except for NRG1, the other 12 genes have been reported. Protein-protein interaction network of 13 genes showed that NRG1 was directly associated with 5 other genes (Figure 1D). To investigate the correlation between NRG1 and pulp repair, we developed a rat pulp injury model and assessed NRG1 expression during inflammation (3 days) and spontaneous mineralization (28 days) of dental pulp. The relative expression of NRG1 was negatively correlated with the necrotic area of the pulp (Pearson *r* = -0.8252, *P* < .05) (Figure 1E and Supplementary Figure S1). NRG1 expression also positively correlated with the region of mineralization (Pearson r = 0.6918, *P* < .01) (Figure 1E and Supplementary Figure S2). Thus, NRG1 expression might be correlated with pulp repair.

We further examined the expression of NRG1 during the spontaneous repair of dental pulp tissue in clinical samples. RNA-seq dataset indicated that NRG1 exhibited low expression, with no significant difference in expression among normal, carious, and pulpitis tissues (Figure 1F). Later, clinical samples revealed that NRG1 was modestly expressed in the pulp of both normal and carious teeth, with no significant difference observed. However, NRG1 expression at the front of the lesion in pulpitis, particularly in the odontoblast layer, was significantly up-regulated (*P < *.01) compared to normal and carious teeth (Figure 1G and Supplementary Figure S3).

To delineate the temporal variations of NRG1, we established a series of rat pulp injury models (Supplementary Figure S4). NRG1 expression was only elevated on the 3rd day (Figure 1H), indicating that NRG1 was only marginally implicated at the designated location and time. Based on the *in vitro-*cultured hDPSCs (Supplementary Figure S5), LPS and MIM were used to replicate the consecutive inflammation and spontaneous OdD of hDPSCs (1 day, 3 days, 7 days, 14 days). LncRNA-seq indicated that NRG1 was not significantly upregulated during LPS stimulation, but it was downregulated in hDPSCs during OdD (Figure 1I). Following GO pathway enrichment analysis, we identified significant enrichment of several inflammation-related pathways (NOD-like receptor signaling pathway, TNF signaling pathway, and NF-κB signaling pathway) after LPS stimulation (Supplementary Figure S6). Conversely, in the MIM condition, osteogenic/odontogenic-related pathways exhibited no significance, with hippo pathway enrichment occurring solely at 14 days (Supplementary Figure S7). The coefficient of variation (CV) was employed to quantify the dispersion of intragroup NRG1 expression among samples, with a CV exceeding 25% being indicative of significant dispersion and substantial intragroup instability.^30^ These findings indicated that NRG1 expression exhibited instability within the group, with coefficients of variation following LPS stimulation of CV(1d) = 0.26, CV(3d) = 0.34, CV(7d) = 0.29, and CV (14d) = 0.21. The coefficient of variation for the OdD is as follows: CV(1d) = 0.30, CV(3d) = 0.37, CV(7d) = 0.24, CV (14d) = 0.37 (Figure 1J). To verify the intragroup variations, hDPSCs from 3 donors were used. After 1 day of LPS stimulation, only one cell strain exhibited up-regulated *NRG1* gene expression. After 3 days of LPS stimulation, *NRG1* expression displayed distinct variations in the three cell strains (Figure 1K). Similarly, NRG1 was programmed to exhibit varied changes temporally (Supplementary Figure S8). After OdD (spontaneous mineralization), *NRG1* gene expression was down-regulated at both day 3 and day 7 (Figure 1L and Supplementary Figure S8). NRG1 protein levels showed no significance following LPS stimulation at 3 hours but were up-regulated at 1 and 2 days (Figure 1M and Supplementary Figure S9). NRG1 was only up-regulated at 14 days of OdD (Figure 1N and Supplementary Figure S9). However, chronologically, NRG1 was down-regulated after LPS stimulation (Supplementary Figure S10A) and up-regulated after OdD (Supplementary Figure S10B). Therefore, expression of NRG1 exhibited significant individual variabilities during inflammation and spontaneous mineralization *in vitro*, which aligned with the restricted capacity of pulp repair noted clinically.

### NRG1 decided on the efficacy of pulp repair

**Figure 2.**
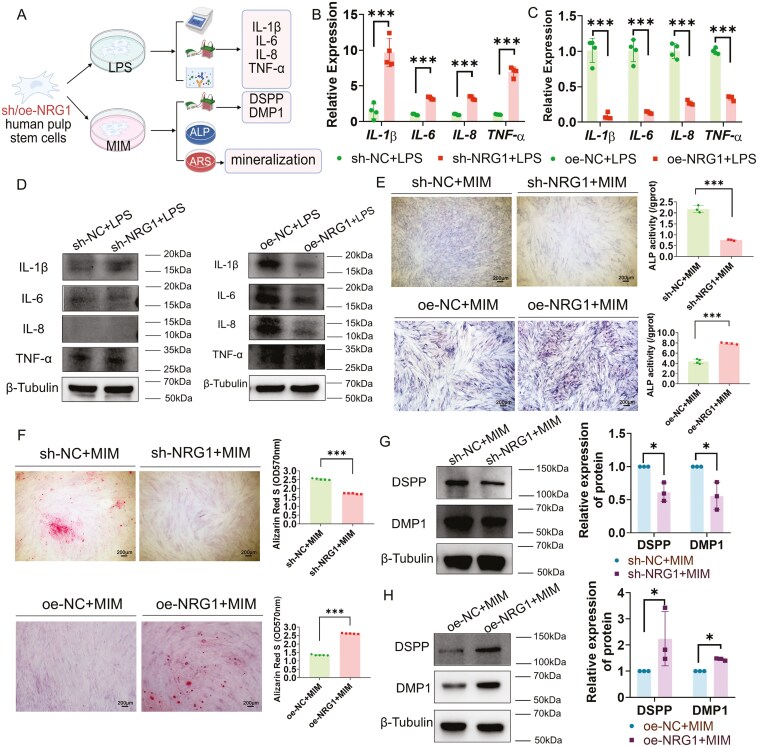
NRG1 expression was linked to the regulation of pulp inflammation and mineralization while demonstrating an *in vitro* inhibitory effect on pulp inflammation and facilitating regeneration. (A) The schematic diagram of effect of NRG1 on pulp inflammation and mineralization. (B) The level of mRNA of proinflammatory factors following NRG1 knockdown by qPCR, *N* = 4. (C) The level of mRNA of pro-inflammatory factors following NRG1 overexpression by qPCR, *N* = 4. (D) The expression of pro-inflammatory factors following NRG1 knockdown/overexpression by Western blot, *N* = 3. (E) The effect of NRG1 knockdown/overexpression on alkaline phosphatase (ALP), *N* = 3-4. Bar, 200μm. (F) The effect of NRG1 knockdown/overexpression on Alizarin red S (ARS), *N* = 5. Bar, 200μm. (G, H) The DSPP, and DMP1 expression following NRG1 knockdown/overexpression by Western blot, *N* = 3.*P < .05, ***P < .001.

The effect of NRG1 was further investigated by establishing NRG1 knockdown (sh-NRG1) and overexpression (oe-NRG1) in hDPSCs via lentiviral transfection (Supplementary Figure S11). The level of mRNA of *IL-1β* (*P < *.001), *IL-6* (*P < *.001), *IL-8* (*P < *.001), and *TNF-α* (*P < *.001) was higher in sh-NRG1 + LPS group than that in sh-NC + LPS group (Figure 2B). Compared with oe-NC + LPS group, the level of mRNA of *IL-1β* (*P < *.001), *IL-6* (*P < *.001), *IL-8* (*P < *.001), and *TNF-α* (*P < *.001) was lower in sh-NRG1 + LPS group (Figure 2C). The expression of IL-1β (*P < *.01), were significantly elevated in the sh-NRG1 + LPS group compared to the sh-NC + LPS group, but there was no difference in the expression of IL-6, IL-8, and TNF-α (*P* >. 05) (Figure 2D and Supplementary Figure S12). The expression of IL-1β (*P < *.001), IL-6 (*P < *.05) and IL-8 (*P < *.01) was significantly reduced in the oe-NRG1 + LPS group compared to the oe-NC + LPS group, but the expression of TNF-α had no difference (*P > *.05) (Figure 2D and Supplementary Figure S12). The ELISA results indicated that NRG1 did not exert a significant impact on the secretion of IL-1β, IL-6, IL-8, and TNF-α (*P > *.05), with minimal release observed for IL-1β and TNFα (Supplementary Figure S13). Thus, NRG1 was proposed to have anti-inflammatory effects by suppressing the production of pro-inflammatory cytokines.

NRG1 knockdown/overexpression hDPSCs underwent ALP staining following 7 days of OdD in MIM. The results indicated reduced ALP staining and activity in the sh-NRG1 + MIM group compared to the sh-NC + MIM group (Figure 2E). ALP staining and activity were augmented in the oe-NRG1 + MIM group compared to the oe-NC + MIM group (*P < *.001, Figure 2E). Alizarin red S staining revealed the presence of distinct mineralized nodules in the oe-NRG1 + MIM group after 14 days of MIM, whereas the oe-NC + MIM group did not exhibit mineralized nodules but displayed darker cellular staining. The optical density values (OD570nm) were markedly elevated in the oe-NRG1 + MIM group in comparison to oe-NC + MIM group (*P < *.001, Figure 2F). After 21 days of OdD, mineralized nodules were evident in the sh-NC + MIM group, while the sh-NRG1 + MIM group exhibited minimal Alizarin Red S staining, with a significantly reduced value compared to the sh-NC + MIM group (*P < *.001, Figure 2F). Following 14 days of OdD, DSPP and DMP1 were downregulated in the sh-NRG1 + MIM group compared to the sh-NC + MIM group (*P < *.05, Figure 2G). Moreover, DSPP and DMP1 exhibited elevated expression levels in the oe-NRG1 + MIM group compared to the oe-NC + MIM group (*P < *.05, Figure 2H).

**Figure 3.**
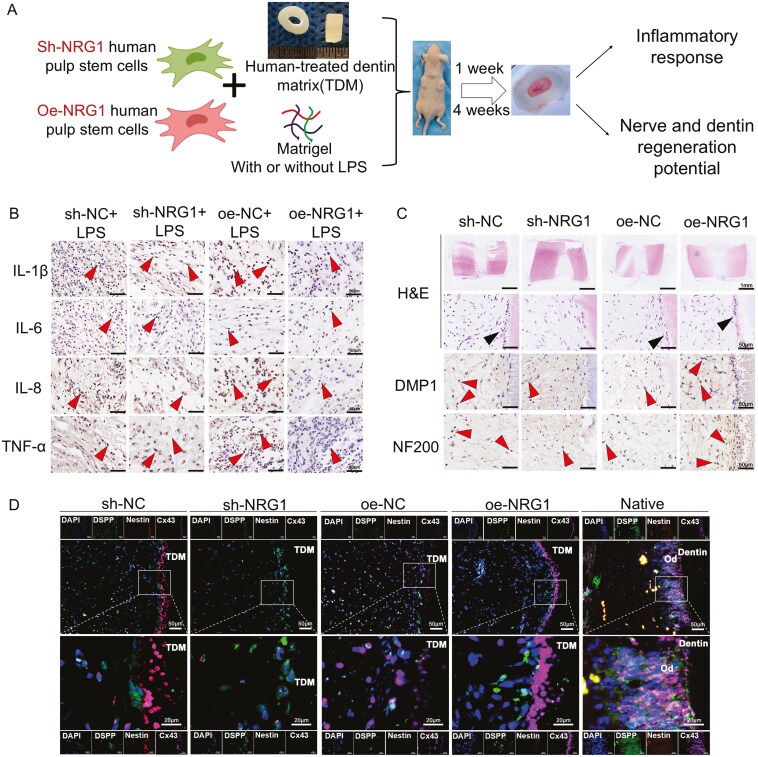
The mobilization of NRG1 promoted the regeneration of both soft and hard tissues within the dental pulp. (A) The *in vivo* experimental paradigm. (B) Expression of pro-inflammatory cytokines following NRG1 knockdown/overexpression *in vivo* by IHC staining, *N* = 6. (C) Impact of NRG1 knockdown/overexpression on mineralization (DMP1) and neuronal differentiation (NF200) *in vivo* by IHC staining. *N* = 8. (D) Impact of NRG1 knockdown/overexpression on the coordinated regeneration of soft and hard tissues *in vivo* by mIHC staining, TDM, human-treated dentin matrix. Od, odontoblast layer. Bar, 50 μm and 20 μm.

Subsequently, we developed an *in vivo* model of NRG1 intervention through knockdown or overexpression of hDPSCs, coupled with TDM transplantation subcutaneously into the dorsal region of nude mice (Figure 3A, Supplementary Figure S14 and S15). We observed increased expression of IL-1β (*P < *.05) and TNF-α (*P < *.001) in the sh-NRG1 + LPS group compared to the sh-NC + LPS group. However, no significant differences were noted for IL-6 or IL-8. In comparison to the oe-NC + LPS group, the levels of IL-1β (*P < *.01), IL-8 (*P < *.05), and TNF-α (*P < *.05) were down-regulated in the oe-NRG1 group, but IL-6 exhibited no significant change (Figure 3B and Supplementary Figure S16). The knockdown of NRG1 gene preferentially enhanced the expression of pro-inflammatory factors, whereas the overexpression of NRG1 inhibited their expression, implicating that NRG1 may contribute to the suppression of inflammation in an *in vivo* model.

The development of pulp-like tissue within the TDM was visualized after 4 weeks (Figure 3C). No discernible odontoblast-like layer was detected in the knockdown of NRG1 group, but a denser odontoblast-like layer was noted at the dentin margins in the sh-NC group, oe-NC group, and oe-NRG1 group. Knockdown of NRG1 led to a reduction in DSPP (Supplementary Figure S17 and S18, *P < *.05) and DMP1 (Figure 3C and Supplementary Figure S18, *P < *.001), nestin (neural differentiation) (Supplementary Figure S17 and S18, *P < *.05), and NF200 (Figure 3C and Supplementary Figure S18, *P < *.05), as well as the CD31 (angiogenesis) (Supplementary Figure S19, *P < *.01). Following the overexpression of NRG1, DSPP (*P < *.05), DMP1 (*P < *.05), Nestin (*P < *.05), and NF200 (*P < *.05) exhibited increased levels (Figure 3C, Supplementary Figure S17 and S18).

To further investigate the clues of pulp regeneration, gap junction protein connexin 43(Cx43), DSPP, and nestin were stained for the integrity of the odontoblasts layer (Figure 3D). High expressions and well organization of nestin, DSPP, and CX43 were observed in the odontoblast layer (palisading patterns) in normal human teeth. Odontoblast-like layers were identified in the sh-NC group, oe-NC group, and oe-NRG1 group. Following the ablation of NRG1, the odontoblasts-like layer was weakened, with both OdD and neuronal differentiation diminished. Following the overexpression of NRG1, an odontoblast-like layer became apparent, with both OdD and neuronal differentiation augmented. Gene editing of NRG1 facilitates both soft and hard integrated regeneration of pulp tissues. Nevertheless, regarding its applicability, can NRG1 and pulp regeneration be regulated without modifying the DNA sequence?

### Restricted role of histone acetylation in epigenetic alterations during the spontaneous repair of dental pulp

**Figure 4.**
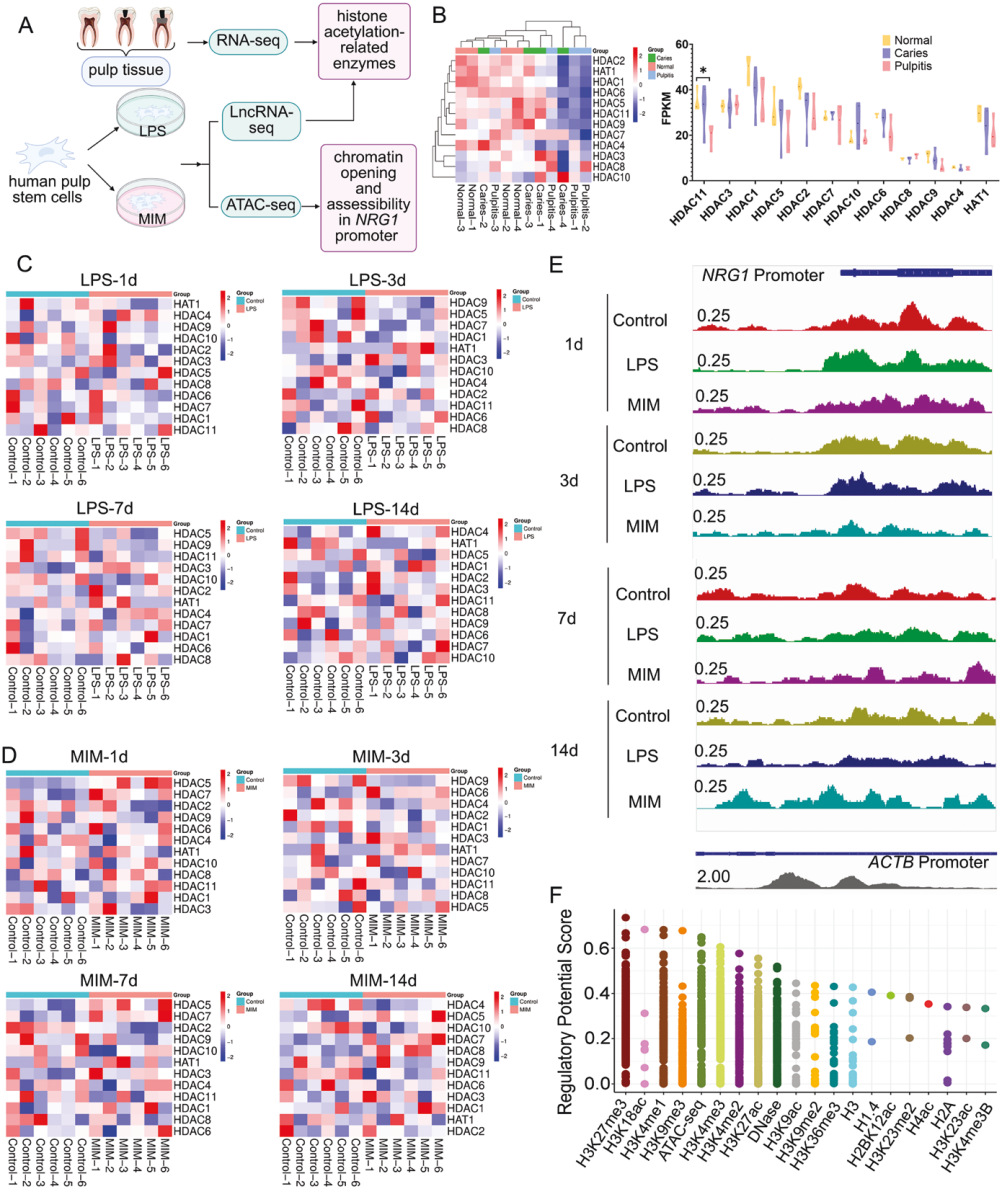
Multi-omics examination of the role of histone acetylation in pulp repair. (A) The schematic diagram of multi-omics examination, including RNA-seq, lncRNA-seq, and ATAC-seq. (B) RNA-seq examination of histone acetylation-related enzymes in spontaneous repair of human dental pulp. The data was from GEO database (GSE198359). The heatmap was acquired by Bioinformatics platform (https://www.bioinformatics.com.cn) (C, D) LncRNA-seq examination of histone acetylation-related enzymes in inflammation and mineralization model of hDPSCs *in vitro*, The heatmap was acquired by Bioinformatics platform (https://www.bioinformatics.com.cn), *N* = 6. (E) Analysis of ATAC-seq about the chromatin opening of the *NRG1* promoter region in inflammation and mineralization model of hDPSCs. The visualization of peak was acquired by Integrative Genomics Viewer (IGV), *N* = 4. (F) Possible regulatory site of histone acetylation for NRG1 from Cistrome Data Browser. *P < .05.

Utilizing the GEO database, we examined pulp tissues from healthy, carious, and pulpitis teeth and identified 12 enzymes associated with histone acetylation (Figure 4B). HDAC11 (*P < *.05) downregulated in the pulpitis group compared to the healthy group, but other enzymes did not change, which indicated histone acetylation may be partly mobilized. We employed lncRNA-seq data from the hDPSCs cultured with LPS or MIM at days 1, 3, 7, and 14. 12 enzymes related to histone acetylation were identified, however, the results indicated no significant changes among them (Figure 4C and D). ATAC-seq was employed to evaluate chromatin opening and the accessibility of the *NRG1* promoter (2000bp upstream to 2000bp downstream of the transcription start site) after LPS and MIM stimulation. If H3K9ac or H3K27ac is present in the *NRG1* promoter, the chromatin would display an open configuration, thereby promoting NRG1 transcription, which could later be detected via ATAC-seq analysis. The peak from ATAC-seq was identified post-data visualization via IGV when the maximum value was set to 0.25 on the vertical axis, while the peak of *ATCB* (positive control to *NRG1*) was set to 2.0. It indicated that the chromatin opening and the accessibility of NRG1 were weak (A smaller set point indicated a weaker chromatin opening). Additionally, it was undetected by Macs2 due to its diminished strength (Figure 4E and Supplementary Figure S20). The four cell strains exhibited inconsistent peaks under identical culture conditions, indicating individual variability in chromatin accessibility. The histone acetylation sites H3K9ac and H3K27ac are closely related to NRG1 (Cistrome Data Browser, Figure 4F). Elevated H3K9ac (*P < *.01) and H3K27ac (*P < *.001) were detected in the pulpitis group (especially in the odontoblast layers) compared to healthy and carious tissues, indicating a pattern of activated gene expressions (Supplementary Figure S21).

**Figure 5.**
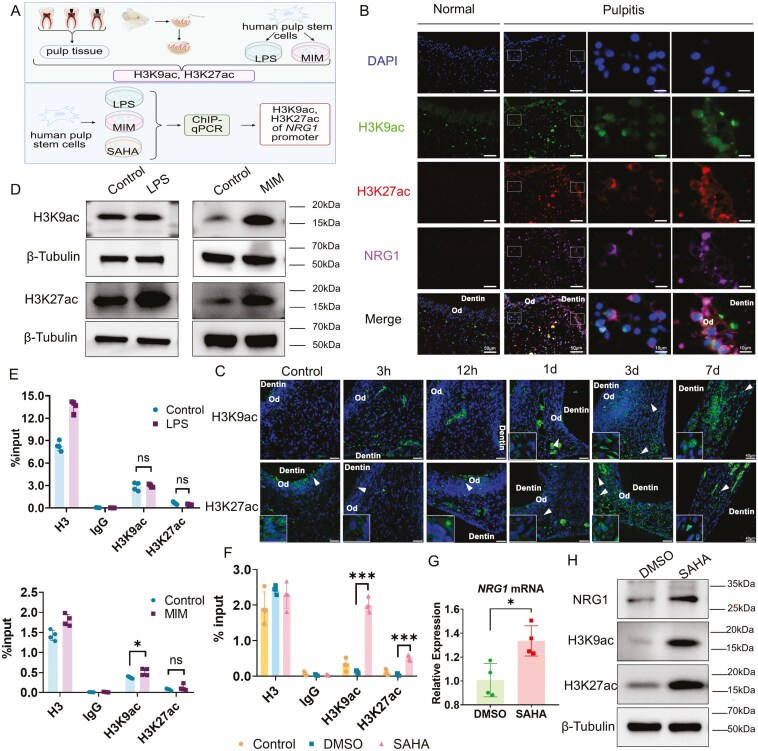
The restricted role of histone acetylation in dental pulp spontaneous repair and the modulation of NRG1. (A) The schematic diagram of histone acetylation (H3K9ac and H3K27ac) in dental pulp spontaneous repair and the modulation of NRG1. (B). Co-localization of NRG1 and histone acetylation (H3K9ac and H3K27ac) by mIHC staining. Bar, 50 μm and 10 μm. (C) Expression of H3K9ac and H3K27ac in a rat model of pulp inflammation by IF staining. *N* = 4. Bar, 40 μm. (D) Expression of H3K9ac and H3K27ac *in vitro* by Western blot, *N* = 3-4. (E) Histone acetylation of H3K9ac and H3K27ac enhances the NRG1 promoter during inflammation and mineralization model *in vitro* by ChIP-qPCR, *N* = 4. (F) Pan-enhanced histone acetylation of H3K9ac and H3K27ac modulates NRG1 by ChIP-qPCR, *N* = 4. (G) Pan-enhanced histone acetylation facilitates the transcription of NRG1 by qPCR, *N* = 4. (H) Pan-enhanced histone acetylation facilitates NRG1 expression by Western blot. *N* = 3-5. Ns, no significance, *P < .05, ***P < .001.

In the rat pulp injury model, H3K9ac and H3K27ac exhibited substantial expression just on day 3 (Figure 5C). Then the *in vitro* results indicated that H3K9ac exhibited no significant alterations during LPS stimulation, whereas H3K27ac was markedly elevated. After OdD, H3K9ac and H3K27ac were increased (Figure 5D and Supplementary Figure S22). Individual variations in histone acetylation also existed. The site-specific and time-restricted alterations in histone acetylation exhibited a pattern analogous to that of NRG1.

Interestingly, H3K9ac and H3K27ac levels correlated with NRG1 expression temporally and spatially. To explore their relationship, we conducted multiplex immunohistochemical staining (mIHC) (Figure 5B) on human dental pulp tissues. Our findings revealed that NRG1, H3K9ac, and H3K27ac were up-regulated in pulpitis tissues, demonstrating a co-expression pattern among all three. Then the enrichment of H3K9ac and H3K27ac in the *NRG1* promoter region was examined by using ChIP-qPCR. The enrichment of H3K9ac and H3K27ac within the promoter region of *NRG1* was not substantially affected by LPS (Figure 5E). The enrichment of H3K9ac in the promoter region of *NRG1* was considerably up-regulated by MIM (*P < *.05), whereas the enrichment of H3K27ac in the same promoter region exhibited no significant change (Figure 5E). Consequently, in pulp inflammation and mineralization, partial histone acetylation may influence *NRG1* transcription by enhancing its association with the *NRG1* promoter, albeit to a limited degree.

### H3K9ac and H3K27ac regulated inflammation and facilitated OdD via NRG1 *in vitro*

The results of ATAC-seq indicated limited chromatin accessibility in the *NRG1* promoter region, corresponding with a low level of histone acetylation (Figure 4E and Supplementary Figure S20). Then the broad-spectrum histone acetylase inhibitor SAHA was used to induce pan-enhanced histone acetylation in hDPSCs.^31^ The enrichment of H3K9ac and H3K27ac in the *NRG1* promoter region was subsequently analyzed. Figure 5F indicated that after 1 day of SAHA treatment on hDPSCs, the enrichment of H3K9ac (*P < *.001) and H3K27ac (*P < *.001) in the *NRG1* promoter region was significantly increased. This indicated enhanced histone acetylation could influence the transcription of *NRG1*. Subsequently, the transcriptional levels of *NRG1* were elevated following pan-enhanced histone acetylation of hDPSCs (Figure 5G, *P < *.05). At the protein level, NRG1 expression was significantly elevated (*P < *.01) in response to the increased levels of H3K9ac (*P < *.001) and H3K27ac (*P < *.01) (Figure 5H and Supplementary Figure S23). Pan-enhanced acetylation facilitated *NRG1* transcription and elevated protein levels by augmenting H3K9ac and H3K27ac enrichment in the *NRG1* promoter area.

**Figure 6.**
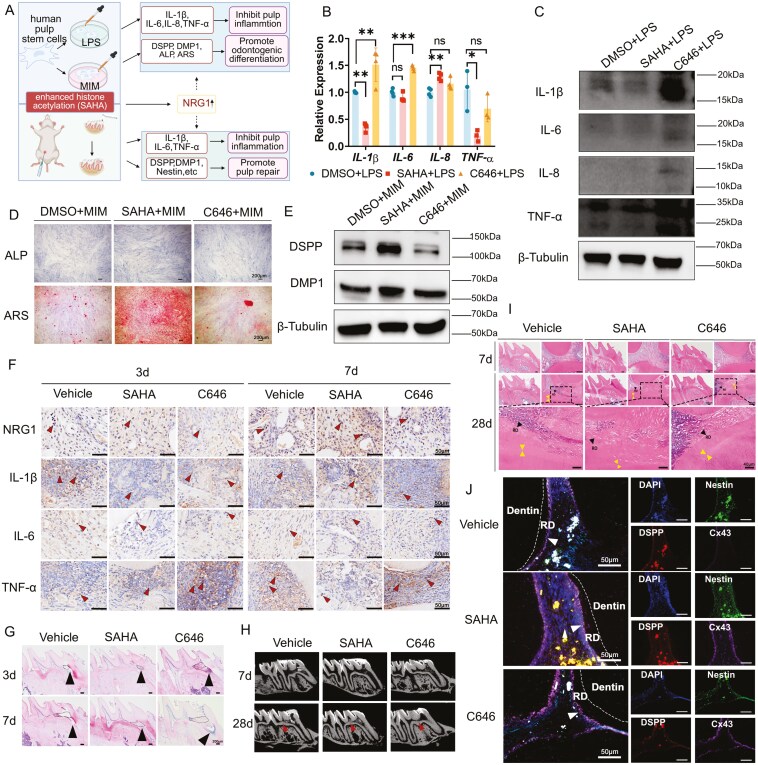
Pan-enhanced histone acetylation enhanced the anti-inflammatory and OdD of hDPSCs, concurrently boosting NRG1 expression and facilitating the integrated healing of soft and hard dental pulp tissues. (A) The schematic diagram of the effect of pan-enhanced histone acetylation on dental pulp repair. (B) The expression of mRNA of proinflammatory factors following pan-enhanced histone acetylation by qPCR, *N* = 3-4. (C) Impact of pan-enhanced histone acetylation on pro-inflammatory factors in hDPSCs by Western blot, *N* = 3. (D) Pan-enhanced histone acetylation enhances the OdD potential of hDPSCs by ALP and ARS, *N* = 5-6. Bar, 200μm (E) Pan-enhanced histone acetylation enhances the expression of DSPP and DMP1 by Western blot, *N* = 3-4. (F) Impact of pan-enhanced histone acetylation on NRG1 and pro-inflammatory factors in rats by IHC staining, *N* = 4-5. Bar, 50 μm (G) Pan-enhanced histone acetylation inhibited the region of the inflammatory region in rats by H&E staining. *N* = 4-5. Bar, 200 μm (H, I) Impact of pan-enhanced histone acetylation on dentin regeneration by H&E staining and micro-CT. *N* = 14-16. Bar, 200 μm and 40 μm. (J) Pan-enhanced histone acetylation facilitates the integrated regeneration of both soft and hard dental pulp tissues by mIHC staining, RD, regenerative dentin. Bar, 50 μm.

Histone acetyltransferases p300 and CBP are responsible for depositing H3K9ac and H3K27ac. The inflammatory and mineralizing effects of hDPSCs were analyzed following SAHA or C646, a p300/CBP inhibitor treatment. SAHA inhibited the expression of *IL-1β* (*P < *.01) and *TNF-α* (*P < *.05) mRNA, increased *IL-8* (*P < *.01) mRNA and had no effect on *IL-6* mRNA (Figure 6B). IL-1β (*P < *.01) were down-regulated in the SAHA + LPS group compared to the DMSO + LPS group, with no significant effect on IL-6, IL-8 and TNF-α. In the C646 + LPS group, IL-1β (*P < *.05), IL-6 (*P < *.05) and IL-8 (*P < *.05) were up-regulated, also showing no significant effect on TNF-α (Figure 6C and Supplementary Figure S24). The secretion of IL-1β, IL-6, IL-8, and TNF-α had no significance among groups (Supplementary Figure S25) by ELISA. After OdD, the results indicated significantly more intense ALP staining and elevated ALP activity in the SAHA + MIM group compared to the DMSO + MIM group (*P < *.001), whereas ALP activity was suppressed in the C646 + MIM group (*P < *.001), which also resulted in diminished ALP staining (Figure 6D and Supplementary Figure S26). These findings indicated that the SAHA + MIM group produced more mineralized nodules compared to the DMSO + MIM group (*P < *.001), while C646 + MIM had reverse results (*P < *.01). The levels of DSPP (*P < *.01) and DMP1 (*P < *.05) were considerably increased in the SAHA + MIM group relative to the DMSO + MIM group, whereas the inhibitory impact of C646 + MIM was not statistically significant (Figure 6E and Supplementary Figure S27).

### Enhanced histone acetylation by SAHA activated NRG1, regulates inflammation and facilitated both soft and hard tissue regeneration of pulp *in vivo*

To investigate the role of H3K9ac and H3K27ac in regulating pulp regeneration, a rat pulp injury model was subsequently developed, with SAHA and C646 administered for 3, 7 and 28 days. At day3, neither pan-enhancement nor inhibition of histone acetylation significantly affected NRG1 expression. At day 7, SAHA significantly increased NRG1 expression compared to the vehicle group (*P* < .05), but C646 had no notable inhibitory effect on NRG1 (Figure 6F and Supplementary Figure S28). It showed that elevated histone acetylation upon SAHA treatment may upregulate NRG1; however, inhibition of histone acetylation was not significant due to the low degree of original acetylation.

We examined the area of pulp necrosis in the inflammatory region (Figure 6G and Supplementary Figure S29). At day 3, the SAHA group did not significantly differ compared to the vehicle group; nevertheless, the C646 group exhibited a greater extent of necrosis (*P < *.05). At day 7, SAHA suppressed the progression of pulp inflammation (*P < *.01). Nonetheless, we observed that on day 3, SAHA markedly reduced the expression of IL-1β (*P < *.05) and IL-6 (*P < *.05). C646 enhanced the expression of TNF-α (*P < *.05), with no notable impact on IL-1β and IL-6. After 7 days, SAHA suppressed the expression of TNF-α (*P < *.01). C646 enhanced the expression of IL-1β (*P < *0.01)(Figure 6F and Supplementary Figure S30).

Micro-CT scanning revealed at 7 days, the images of calcification in the rat's pulp cavity were less discernible; however, at 28 days, a distinct image was evident in the inferior wall of the medullary cavity (Figure 6J). We examined BMD, BV/TV, Tb.Th, Tb.*N*, and Tb.Sp to access mineralization^32^ (Supplementary Figure S31). The results indicated that after 7 days, BMD, BV/TV, Tb.Th, Tb.N, and Tb.Sp exhibited no significant changes in the SAHA group compared to the vehicle group. BMD (*P < *.05), Tb.N (*P < *.01) dropped and Tb.Sp (*P < *.01) increased in the C646 group. It indicates that C646 may exert a minor inhibitory influence on dentin regeneration after 7 days. At 28 days, the SAHA group exhibited increased BMD (*P < *.01) and BV/TV (*P < *.05), compared to the vehicle group. All parameters remained unchanged in the C646 group. This indicated that SAHA may facilitate odontogenesis. Dentin regeneration within the pulp was additionally examined by H&E staining. At 7 days, negligible newformed dentin was detected; however, after 28 days, a layer of newformed dentin was noted accumulating on the bottom of the pulp cavity, with a more distinct boundary with the primary dentin. The SAHA group exhibited a greater formation of newborn dentin, nearly fulfilling the pulp cavity (Figure 6H and Supplementary Figure S31).

To assess the impact of SAHA on the regeneration of both soft and hard tissues, we analyzed the nestin, NF200, DSPP, DMP1, and CD31. At 3 days, nestin staining exhibited no notable alterations in either the SAHA group or the C646 group compared to the vehicle group. At 7 days, notable nestin expression was evident in the mature odontoblasts-like layer, which was further augmented by the SAHA group (*P < *.01, Supplementary Figure S32). Nonetheless, NF200 was hardly detected in the tissue, with only minimal expression noted in the SAHA group (Supplementary Figure S32). The results indicated that the expression levels of NRG1 (*P < *.05), DSPP(*P < *.01), and DMP1(*P < *.05) were elevated in the SAHA group. None of the expressions in the C646 group exhibited substantial changes (Supplementar Figure S32). The SAHA group increased CD31 expression at 7 days, whereas the C646 group showed no significant difference compared to the vehicle group at 3 and 7 days (Supplementary Figure S33). To further investigate the integrated regeneration of soft and hard tissues, Cx43, DSPP, and nestin were co-stained (Figure 6J). These findings indicated that DSPP, nestin, and Cx43 can be concurrently expressed, with their expression augmented by upregulating histone acetylation.

## Discussion

The repairing capacity of the dental pulp is limited, potentially due to some vital components being dormant, rendering them less likely to be triggered spontaneously. The “dormant” components may represent a novel way for pulp repair.^13-15^Figure 1 depicts a result consistent with the clinical observation of NRG1 unstable expression pattern during pulp self-repair, marked by site-specificity (in clinical samples), temporal variability (in animal models), and individual variability. NRG1 expression was increased in cases showing enhanced pulp repair, as demonstrated in the rat pulp injury model (Figure 1E). NRG1 was up-regulated in human pulpitis samples, particularly within the odontoblast layer, whereas in the rat central pulp, rather than in the odontoblasts. This could be related to the variations in models. The human pulpitis samples were obtained from individuals diagnosed with chronic pulpitis, whereas the pulp injury in rats is considered to be acute. DPSCs and odontoblasts play distinct roles at various stages of pulp injury. The lncRNA-seq results from six hDPSCs strains indicated that NRG1 expression exhibited variability among strains. The expression of NRG1 in the LPS groups exhibits a decreasing trend from day 1 to day 14 (Figure 1I and K). Three additional strains of hDPSCs were used to confirm the findings. The LPS group demonstrated significant variations in the mRNA levels of NRG1. The protein levels also decreased from day 0 to 2 (Supplementary Figure S10). RNA-seq and qPCR investigations of six and three cell strains showed a steady reduction in the MIM group from days 1 to 14. However, the protein concentration of NRG1 demonstrated an increase across three cell strains. As hypothesized, some post-transcriptional regulatory mechanisms may be involved ^33^ In certain conditions, NRG1 may be dormant and hence incapable of assuming an active role. Activating “quiescent” NRG1 may be a feasible approach to augment pulp repair.^15^ The hypothesis is both intriguing and compelling. Consequently, it is imperative to validate the function of NRG1 in dental pulp repair.

We conducted *in vitro* studies by manipulating hDPSCs to knock down and overexpress *NRG1* through gene editing. The effect of NRG1 on pulp inflammation and mineralization was assessed. The current experiment revealed the effects of NRG1 on IL-6 and TNF-α. However, NRG1 had little effect on the secretion of proinflammatory factors. IL-1β and TNF-α secretion were difficult to detect by ELISA. Our previous study showed that the secretion of IL-β was little when human periodontal ligament fibroblasts were stimulated by *E. coli* LPS (about 20 pg/mL).^34^NRG1 may act as a resistance to inflammation mainly by inhibiting IL-1β production. In terms of mineralization, it has been shown that NRG1 does not have a promoting effect on the osteogenic differentiation of periodontal ligament stem cells (PLSCs).^35^ However, it is essential for cartilage regeneration in zebrafish.^36^ Macrophage-secreted NRG1 recruits periosteal stem cells, which is crucial for bone repair.^20^ Our study, on the other hand, shows that NRG1 facilitates the differentiation of hDPSCs. Subcutaneously transplanted oe-NRG1 hDPSCs exhibited enhanced OdD capability and facilitated the development of the odontoblast-like layer. This suggests that NRG1 can play a positive role in dental pulp repair.

NRG1 also augments the neuronal and vascular differentiation capabilities of hDPSCs. hDPSCs have some neural differentiation potential and are stained for nestin and NF200.^37,38^ The findings showed that NRG1 enhanced hDPSCs to differentiate neuronally. It was reported that hDPSCs with elevated NRG1 expression facilitate facial nerve regeneration.^18^ Additionally, NRG1 also affects vascularization. It has been shown that NRG1 promotes angiogenic differentiation of PLSCs and upregulates CD31 to promote vascularization.^35^ NRG1 promotes myocardial angiogenesis in rats with diabetic cardiomyopathy.^39^ Bone marrow stem cells overexpressing NRG1 favor angiogenesis in ischemic skeletal muscle.^40^ In this study, *NRG1* knockdown inhibited angiogenesis, suggesting that NRG1 deficiency affects angiogenesis. The palisading patterns, fenestrated configuration of the odontoblast-like layer, are crucial for effective pulp repair.^41^ Cx43 is crucial for preserving the organization of odontoblasts and influences the efficacy of pulp repair by regulating cell differentiation.^41,42^ NRG1 enhances the expression of Cx43, thus aiding in the organization of odontoblasts. Furthermore, NRG1 increased the expression of nestin, DSPP, illustrating its positive impact on the nerve and odontoblasts, culminating in “pulp regeneration.”

However, all of these exciting results have been performed by gene editing. Taking into account the feasibility of future clinical applications, gene editing alters DNA sequence.^43^ Histone acetylation, an epigenetic alteration, regulates genes without modifying the DNA sequence and is mostly linked to gene activation.^44^ Histone acetylation promotes the expression of NRG1.^25^ We found weak chromatin opening in the *NRG1* promoter region by ATAC-seq under spontaneous repair, which is consistent with the results of lncRNA-seq (Figure 1F). The multi-omics analysis and validation of RNA-seq, lncRNA-seq, and ATAC-seq illustrated that in the case of spontaneous pulp repair, histone acetylation also had a limited role and did not significantly regulate NRG1. In the case of pulp injury, despite the occurrence of histone acetylation, its insufficient expression may fail to adequately activate NRG1 expression, leading to preferential histone acetylation at alternative gene loci and diminished enrichment at the *NRG1* promoter region.^45^ This suggests that turning “ON” histone acetylation is one of the possible strategies and regulatory mechanisms to enhance NRG1, thereby promoting pulp regeneration.

Epigenetic drugs are compounds that interact with the cellular epigenome to exert their effects, including DNA methyltransferases, DNA demethylases, HDAC, histone acetyltransferases, histone methyltransferases, and histone demethylases.^22^ Given the diminished histone acetylation during the spontaneous pulp repair and lack of selective histone deacetylase inhibitor, we employed a broad-spectrum histone deacetylase inhibitor, SAHA, which enhances histone acetylation.^46-48^ The present study showed SAHA increased the enrichment of both H3K9ac and H3K27ac within the *NRG1* promoter region, resulting in chromatin opening of *NRG1* promoter and the activation of *NRG1*. SAHA selectively inhibited the expression of pro-inflammatory factors by regulating NRG1. In addition, SAHA promoted OdD of hDPSCs, which is consistent with previous studies.^49^ SAHA can affect Wnt, Notch pathways, etc.,^50,51^ which may regulate OdD. *In vivo*, pan-enhanced histone acetylation promoted pulp regeneration while increasing NRG1 expression. In addition, SAHA promoted the expression of nestin, DSPP, and Cx43, further suggesting that pan-enhancer histone acetylation facilitates pulp regeneration. Thus, enhancing histone acetylation by SAHA could promote pulp regeneration via modulating NRG1. However, SAHA may have non-specific effects on many other genes. Therefore, it is worthwhile to explore the specific regulation of histone acetylation by targeting NRG1 and to develop specific histone acetylation drugs in the future.

## Conclusions

NRG1 and histone acetylation may be dormant in pulp spontaneous repair. The limited regulation of NRG1 by histone acetylation could impact pulp repair. The overexpression of NRG1 enhanced the anti-inflammation capacity and integrated regeneration of both soft and hard pulp tissues, while comprehensive enhancement of histone acetylation presented a feasible and effective method to activate NRG1. Consequently, enhanced histone acetylation may be pivotal in augmenting anti-inflammatory and regeneration of dental pulp through the modulation of NRG1.

## Supplementary Material

szaf022_suppl_Supplementary_Tables

szaf022_suppl_Supplementary_Material

## Data Availability

The data that support the findings of this study are available from the corresponding author upon reasonable request.
