# Supplementary material for: Histone acetylation facilitates multidirectional pulp repair through Neuregulin-1 mobilization
Source: Stem Cells Transl Med. 2025 Jun 28;14(7):szaf022. doi: 10.1093/stcltm/szaf022 (PMC12205360; doi:10.1093/stcltm/szaf022)
Supplement: szaf022_suppl_Supplementary_Tables [file szaf022_suppl_supplementary_tables.docx]

Table 1. The *P* value of IHC staining of NRG1 in pulp tissue from normal, caries and pulpitis teeth (Fig.1G and S3).

| Groups | *P* value* |
| --- | --- |
| Normal vs. Caries | 0.6637 |
| Normal vs. Pulpitis | 0.0024 |
| Caries vs. Pulpitis | 0.0086 |

* One-way Anova test

Table 2. The *P* value of validation of NRG1 gene expression in inflammation (LPS)/ mineralization (MIM) model of hDPSCs in vitro by qPCR (Fig.1K and 1L).

| Groups | Cell | *P* value* |
| --- | --- | --- |
| Control vs. LPS 1d | Cell 1 | 0.3050 |
| Control vs. LPS 1d | Cell 2 | 0.0003 |
| Control vs. LPS 1d | Cell 3 | 0.3162 |
| Control vs. LPS 3d | Cell 1 | 0.0004 |
| Control vs. LPS 3d | Cell 2 | 0.0087 |
| Control vs. LPS 3d | Cell 3 | 0.8803 |
| Control vs.MIM 3d | Cell 1 | <0.0001 |
| Control vs.MIM 3d | Cell 2 | <0.0001 |
| Control vs.MIM3d | Cell 3 | <0.0001 |
| Control vs.MIM 7d | Cell 1 | <0.0001 |
| Control vs.MIM 7d | Cell 2 | <0.0001 |
| Control vs.MIM7d | Cell 3 | <0.0001 |

* t-test

Table 3 The *P* value of temporal variation of NRG1 mRNA during inflammation (LPS)/ mineralization (MIM) model in vitro among cells (Fig S8)

| Groups | Cell | *P* value* |
| --- | --- | --- |
| Control vs. LPS 1d | Cell 1 | 0.3813 |
| Control vs. LPS 3d | Cell 1 | 0.0978 |
| LPS 1d vs. LPS 3d | Cell 1 | 0.0172 |
| Control vs. LPS 1d | Cell 2 | 0.1800 |
| Control vs. LPS 3d | Cell 2 | <0.0001 |
| LPS 1d vs. LPS 3d | Cell 2 | <0.0001 |
| Control vs. LPS 1d | Cell 3 | 0.4036 |
| Control vs. LPS 3d | Cell 3 | 0.7536 |
| LPS 1d vs. LPS 3d | Cell 3 | 0.1651 |
| Control vs.MIM 3d | Cell 1 | <0.0001 |
| Control vs.MIM 7d | Cell 1 | <0.0001 |
| MIM 3d vs.MIM 7d | Cell 1 | 0.1846 |
| Control vs.MIM 3d | Cell 2 | <0.0001 |
| Control vs.MIM 7d | Cell 2 | <0.0001 |
| MIM 3d vs.MIM 7d | Cell 2 | 0.3760 |
| Control vs.MIM 3d | Cell 3 | <0.0001 |
| Control vs.MIM 7d | Cell 3 | <0.0001 |
| MIM 3d vs.MIM 7d | Cell 3 | 0.0003 |

* One-way Anova test

Table 4. The *P* value of western blot of NRG1 during inflammation and mineralization model *in vitro* (Fig 1M,1N and S9)

| Groups | *P* value* |
| --- | --- |
| Control vs. LPS 3h | 0.2000 |
| Control vs. LPS 1d | 0.0159 |
| Control vs. LPS 2d | 0.0145 |
| Control vs. MIM 3d | 0.9671 |
| Control vs. LPS 7d | 0.3705 |
| Control vs. LPS 14d | 0.0237 |

* t-test

Table 5. The *P* value of the temporal variation of NRG1 during inflammation and mineralization model *in vitro* (Fig. S10)

| Groups | *P* value* |
| --- | --- |
| Control vs. LPS 3h | 0.9993 |
| Control vs. LPS 1d | 0.0143 |
| Control vs. LPS 2d | 0.0057 |
| LPS 3h vs. LPS 1d | 0.0168 |
| LPS 3h vs. LPS 2d | 0.0066 |
| LPS1d vs. LPS 2d | 0.8897 |
| Control vs. MIM 3d | 0.9939 |
| Control vs. MIM 7d | 0.0439 |
| Control vs. MIM 14d | 0.0025 |
| MIM 3d vs. MIM 7d | 0.0309 |
| MIM 3d vs. MIM 14d | 0.0019 |
| MIM 7d vs. MIM 14d | 0.1992 |

* One-way Anova test

Table 6. The *P* value of mRNA of proinflammatory factors following NRG1 knockdown/overexpression by qPCR (Fig.2B and 2C)

| Groups | Proinflammatory factors | *P* value* |
| --- | --- | --- |
| sh-NC+LPS vs. sh-NRG1+LPS | IL-1β | 0.0003 |
|  | IL-6 | <0.0001 |
|  | IL-8 | <0.0001 |
|  | TNF-α | <0.0001 |
| oe-NC+LPS vs. oe-NRG1+LPS | IL-1β | <0.0001 |
|  | IL-6 | <0.0001 |
|  | IL-8 | <0.0001 |
|  | TNF-α | <0.0001 |

* t-test

Table 7. The *P* value of pro-inflammatory factors following NRG1 knockdown/overexpression by western blot (Fig.2D and S12)

| Groups | Proinflammatory factors | *P* value* |
| --- | --- | --- |
| sh-NC+LPS vs. sh-NRG1+LPS | IL-1β | 0.0092 |
|  | IL-6 | 0.5779 |
|  | IL-8 | 0.5854 |
|  | TNF-α | 0.1577 |
| oe-NC+LPS vs. oe-NRG1+LPS | IL-1β | 0.0001 |
|  | IL-6 | 0.0378 |
|  | IL-8 | 0.0050 |
|  | TNF-α | 0.2273 |

* t-test

Table 8. The *P* value of proinflammatory factors secretion following NRG1 knockdown/overexpression by ELISA (Fig.S13)

| Groups | Proinflammatory factors | *P* value* |
| --- | --- | --- |
| sh-NC+LPS vs. sh-NRG1+LPS | IL-1β | 0.1034 |
|  | IL-6 | 0.5494 |
|  | IL-8 | 0.9640 |
|  | TNF-α | 0.1340 |
| oe-NC+LPS vs. oe-NRG1+LPS | IL-1β | 0.5776 |
|  | IL-6 | 0.2417 |
|  | IL-8 | 0.8607 |
|  | TNF-α | 0.8422 |

* t-test

Table 9. The *P* value of NRG1 knockdown/overexpression on alkaline phosphatase (ALP) and Alizarin red S (ARS) (Fig.2E and 2F)

| Groups | ALP/ARS | *P* value* |
| --- | --- | --- |
| sh-NC+MIM vs. sh-NRG1+MIM | ALP | 0.0001 |
|  | ARS | <0.0001 |
| oe-NC+MIM vs. oe-NRG1+MIM | ALP | <0.0001 |
|  | ARS | <0.0001 |

* t-test

Table 10. The *P* value of NRG1 knockdown/overexpression on DSPP and DMP1 (Fig.2G and 2H)

| Groups | DSPP/DMP1 | *P* value* |
| --- | --- | --- |
| sh-NC+MIM vs. sh-NRG1+MIM | DSPP | 0.0431 |
|  | DMP1 | 0.0300 |
| oe-NC+MIM vs. oe-NRG1+MIM | DSPP | 0.0210 |
|  | DMP1 | 0.0150 |

* t-test

Table 11. The *P* value of verification of NRG1 expression of NRG1 knockdown/overexpression in vivo (Fig S14 and S15)

| Groups | *P* value* |
| --- | --- |
| sh-NC+LPS vs. sh-NRG1+LPS | 0.0271 |
| oe-NC+LPS vs. oe-NRG1+LPS | 0.0079 |
| sh-NC vs. sh-NRG1 | 0.0039 |
| oe-NC vs. oe-NRG1 | 0.0192 |

* t-test

Table 12. The *P* value of IHC staining of of proinflammatory factors following NRG1 knockdown/overexpression in vivo (Fig.3B and S16).

| Groups | Proinflammatory factors | *P* value* |
| --- | --- | --- |
| sh-NC+LPS vs. sh-NRG1+LPS | IL-1β | 0.0203 |
|  | IL-6 | 0.5587 |
|  | IL-8 | 0.3192 |
|  | TNF-α | <0.0001 |
| oe-NC+LPS vs. oe-NRG1+LPS | IL-1β | 0.0073 |
|  | IL-6 | 0.3622 |
|  | IL-8 | 0.0201 |
|  | TNF-α | 0.0275 |

* t-test

Table 13. The *P* value of IHC staining of mineralization (DSPP and DMP1), neuronal differentiation (Nestin and NF200) and CD 31 following NRG1 knockdown/overexpression *in vivo* (Fig 3C, S17-19).

| Groups | Proinflammatory factors | *P* value* |
| --- | --- | --- |
| sh-NC vs. sh-NRG1 | DSPP | 0.0246 |
|  | DMP1 | 0.0007 |
|  | Nestin | 0.0170 |
|  | NF200 | 0.0205 |
|  | CD31 | 0.0088 |
| oe-NC vs. oe-NRG1 | DSPP | 0.0297 |
|  | DMP1 | 0.0010 |
|  | Nestin | 0.0011 |
|  | NF200 | 0.0272 |
|  | CD31 | 0.3811 |

* t-test

Table 14. The *P* value of RNA-seq examination of histone acetylation-related enzymes in spontaneous repair of human dental pulp (Fig.4B).

| Groups | histone acetylation-related enzymes | *P* value* |
| --- | --- | --- |
| Normal vs. Pulpitis | HDAC11 | 0.0473 |

Only show the comparison that was different significantly.

* One-way Anova test

Table 15. The *P* value of expression of H3K9ac and H3K27ac in human pulp (Fig. S21)

| Groups | H3K9ac and H3K27ac | *P* value* |
| --- | --- | --- |
| Normal vs. Caries | H3K9ac | 0.8714 |
| Normal vs. Pulpitis | H3K9ac | 0.0011 |
| Caries vs. Pulpitis | H3K9ac | 0.0022 |
| Normal vs. Caries | H3K27ac | 0.4885 |
| Normal vs. Pulpitis | H3K27ac | <0.0001 |
| Caries vs. Pulpitis | H3K27ac | 0.0003 |

* One-way Anova test

Table 16. The *P* value of western blot of H3K9ac and H3K27ac during inflammation and mineralization model *in vitro* (Fig.5D and S22)

| Groups | H3K9ac and H3K27ac | *P* value* |
| --- | --- | --- |
| Control vs. LPS | H3K9ac | 0.3812 |
| Control vs. LPS | H3K27ac | <0.0001 |
| Control vs. MIM | H3K9ac | 0.0104 |
| Control vs. MIM | H3K27ac | 0.0167 |

* t-test

Table 16. The *P* value of histone acetylation of H3K9ac and H3K27ac enhances the NRG1 promoter during inflammation and mineralization model in vitro by ChIP-qPCR (Fig.5E)

| Groups | H3K9ac and H3K27ac | *P* value* |
| --- | --- | --- |
| Control vs. LPS | H3K9ac | 0.7440 |
| Control vs. LPS | H3K27ac | 0.0720 |
| Control vs. MIM | H3K9ac | 0.0286 |
| Control vs. MIM | H3K27ac | 0.3145 |

* t-test

Table 17. The *P* value of pan-enhanced histone acetylation of H3K9ac and H3K27ac modulated NRG1 by ChIP-qPCR (Fig.5F)

| Groups | H3K9ac and H3K27ac | *P* value* |
| --- | --- | --- |
| DMSO vs. SAHA | H3K9ac | <0.0001 |
| DMSO vs. SAHA | H3K27ac | <0.0001 |

* t-test

Table 18. The *P* value of pan-enhanced histone acetylation facilitates the transcription of NRG1 by qPCR (Fig.5G)

| Groups | *P* value* |
| --- | --- |
| DMSO vs. SAHA | 0.0130 |

* t-test

Table 19. The *P* value of pan-enhanced histone acetylation facilitates NRG1 expression by western blot (Fig.5H and S23)

| Groups | H3K9ac, H3K27ac and NRG1 | *P* value* |
| --- | --- | --- |
| DMSO vs. SAHA | H3K9ac | 0.0009 |
| DMSO vs. SAHA | H3K27ac | 0.0054 |
| DMSO vs. SAHA | NRG1 | 0.0012 |

* t-test

Table 20. The *P* value of mRNA of proinflammatory factors following pan-enhanced histone acetylation by qPCR (Fig.6B)

| Groups | Proinflammatory factors | *P* value* |
| --- | --- | --- |
| DMSO+LPS vs. SAHA+LPS | IL-1β | 0.0021 |
|  | IL-6 | 0.1300 |
|  | IL-8 | 0.0027 |
|  | TNF-α | 0.0183 |
| DMSO+LPS vs. C646+LPS | IL-1β | 0.0086 |
|  | IL-6 | <0.0001 |
|  | IL-8 | 0.0661 |
|  | TNF-α | 0.3147 |

* One-way Anova test

Table 21. The *P* value of pan-enhanced histone acetylation on pro-inflammatory factors in hDPSCs by western blot (Fig.6C and S24)

| Groups | Proinflammatory factors | *P* value* |
| --- | --- | --- |
| DMSO+LPS vs. SAHA+LPS | IL-1β | 0.0074 |
|  | IL-6 | 0.4481 |
|  | IL-8 | 0.9365 |
|  | TNF-α | 0.9007 |
| DMSO+LPS vs. C646+LPS | IL-1β | 0.0258 |
|  | IL-6 | 0.0172 |
|  | IL-8 | 0.0349 |
|  | TNF-α | 0.3819 |

* One-way Anova test

Table 22. The *P* value of proinflammatory factors secretion during inflammation following pan-enhanced histone acetylation *in vitro* (Fig. S25)

| Groups | Proinflammatory factors | *P* value* |
| --- | --- | --- |
| DMSO+LPS vs. SAHA+LPS | IL-1β | 0.5287 |
|  | IL-6 | 0.9550 |
|  | IL-8 | 0.7062 |
|  | TNF-α | 0.6410 |
| DMSO+LPS vs. C646+LPS | IL-1β | 0.7140 |
|  | IL-6 | 0.6686 |
|  | IL-8 | 0.6106 |
|  | TNF-α | >0.9999 |

* One-way Anova test

Table 23. The *P* value of pan-enhanced histone acetylation enhances the OdD potential of hDPSCs by ALP and ARS (Fig. 6D and S26)

| Groups | ALP/ARS | *P* value* |
| --- | --- | --- |
| DMSO+MIM vs. SAHA+MIM | ALP | <0.0001 |
|  | ARS | <0.0001 |
| DMSO+MIM vs. C646+MIM | ALP | <0.0001 |
|  | ARS | 0.0064 |

* One-way Anova test

Table 24. The *P* value of pan-enhanced histone acetylation enhances the expression of DSPP and DMP1 by western blot (Fig.6E and S27)

| Groups | DSPP/DMP1 | *P* value* |
| --- | --- | --- |
| DMSO+MIM vs. SAHA+MIM | DSPP | 0.0095 |
|  | DMP1 | 0.0284 |
| DMSO+MIM vs. C646+MIM | DSPP | 0.9186 |
|  | DMP1 | 0.1279 |

* One-way Anova test

Table 25. The *P* value of NRG1 in rat pulp injury tissue following pan-enhanced histone acetylation (Fig. 6F and S28)

| Groups | 3d/7d | *P* value* |
| --- | --- | --- |
| DMSO vs. SAHA | 3d | 0.9741 |
|  | 7d | 0.0195 |
| DMSO vs. C646 | 3d | 0.1363 |
|  | 7d | 0.6760 |

* One-way Anova test

Table 26. The *P* value of pan-enhanced histone acetylation on pro-inflammatory factors in rats by IHC staining (Fig.6F and S30)

| Groups | Proinflammatory factors | *P* value* |
| --- | --- | --- |
| DMSO vs. SAHA 3d | IL-1β | 0.0179 |
|  | IL-6 | 0.0120 |
|  | TNF-α | 0.9959 |
| DMSO vs. C646 3d | IL-1β | 0.7120 |
|  | IL-6 | 0.2126 |
|  | TNF-α | 0.0317 |
| DMSO vs. SAHA 7d | IL-1β | 0.3115 |
|  | IL-6 | 0.1272 |
|  | TNF-α | 0.0013 |
| DMSO vs. C646 7d | IL-1β | 0.0368 |
|  | IL-6 | 0.8759 |
|  | TNF-α | 0.0972 |

* One-way Anova test

Table 27. The *P* value of inflammatory region in rats following pan-enhanced histone acetylation (Fig.6G and S29).

| Groups | 3d/7d | *P* value* |
| --- | --- | --- |
| DMSOvs. SAHA | 3d | 0.9205 |
|  | 7d | 0.0045 |
| DMSO vs. C646 | 3d | 0.0441 |
|  | 7d | 0.1883 |

* One-way Anova test

Table 28. The *P* value of Micro-CT quantitative analysis of histone acetylation in intervention of pulpitis in rats and regeneration of hard tissue in the pulp (Fig.6H and S31).

| Groups | index | *P* value* |
| --- | --- | --- |
| DMSO vs. SAHA 7d | BMD | 0.6900 |
| DMSO vs. C646 7d |  | 0.0114 |
| DMSO vs. SAHA 28d |  | 0.0076 |
| DMSO vs. C646 28d |  | 0.9038 |
| DMSO vs. SAHA 7d | BV/TV | 0.7056 |
| DMSO vs. C646 7d |  | 0.0973 |
| DMSO vs. SAHA 28d |  | 0.0109 |
| DMSO vs. C646 28d |  | 0.9751 |
| DMSO vs. SAHA 7d | Tb.Th | 0.9963 |
| DMSO vs. C646 7d |  | 0.7450 |
| DMSO vs. SAHA 28d |  | 0.1908 |
| DMSO vs. C646 28d |  | 0.6365 |
| DMSO vs. SAHA 7d | Tb.Sp | 0.1117 |
| DMSO vs. C646 7d |  | 0.0023 |
| DMSO vs. SAHA 28d |  | 0.1406 |
| DMSO vs. C646 28d |  | 0.5857 |
| DMSO vs. SAHA 7d | Tb.N | 0.0522 |
| DMSO vs. C646 7d |  | 0.0023 |
| DMSO vs. SAHA 28d |  | 0.1406 |
| DMSO vs. C646 28d |  | 0.5857 |

* One-way Anova test

Table 29. The *P* value of pan-enhanced histone acetylation in nerve and dentin repair (Fig.S32)

| Groups | index | *P* value* |
| --- | --- | --- |
| DMSO vs. SAHA | NRG1 | 0.0114 |
| DMSO vs. C646 |  | >0.9999 |
| DMSO vs. SAHA 7d | Nestin | 0.0052 |
| DMSO vs. C646 7d |  | 0.2104 |
| DMSO vs. SAHA 7d | DSPP | 0.0062 |
| DMSO vs. C646 7d |  | 0.9997 |
| DMSO vs. SAHA 7d | DMP1 | 0.0245 |
| DMSO vs. C646 7d |  | 0.9979 |

* One-way Anova test
