# Supplementary material for: Histone acetylation facilitates multidirectional pulp repair through Neuregulin-1 mobilization
Source: Stem Cells Transl Med. 2025 Jun 28;14(7):szaf022. doi: 10.1093/stcltm/szaf022 (PMC12205360; doi:10.1093/stcltm/szaf022)
Supplement: szaf022_suppl_Supplementary_Material [file szaf022_suppl_supplementary_material.docx]

Histone acetylation facilitates multidirectional pulp repair through Neuregulin-1 mobilization

Running head: Histone acetylation promotes pulp repair via NRG1

Zhiwu Wu^1,2‡^, Hui Yang^1‡^, Shaoying Duan^1^, Qianqian Su^1^, Ran Cheng^1*^, Tao Hu^1*^

^1^State Key Laboratory of Oral Diseases & National Center for Stomatology & National Clinical Research Center for Oral Diseases & Frontier Innovation Center for Dental Medicine Plus, West China Hospital of Stomatology, Sichuan University, Chengdu, 610041, China

^2^ School of Stomatology, Zhejiang Chinese Medical University, Stomatology Hospital affiliated with Zhejiang Chinese Medical University, Hangzhou, 310053, China

^*^ Correspondence

Tao Hu, Doctor

State Key Laboratory of Oral Diseases & National Center for Stomatology & National Clinical Research Center for Oral Diseases & Frontier Innovation Center for Dental Medicine Plus, West China Hospital of Stomatology, Sichuan University, Chengdu, 610041, China.

E-mail: [hutao@scu.edu.cn](mailto:hutao@scu.edu.cn) Tel. 00-86-028-85503486

Ran Cheng, Doctor

State Key Laboratory of Oral Diseases & National Center for Stomatology & National Clinical Research Center for Oral Diseases & Frontier Innovation Center for Dental Medicine Plus, West China Hospital of Stomatology, Sichuan University, Chengdu, 610041, China.

E-mail: [chengran@scu.edu.cn](mailto:chengran@scu.edu.cn) Tel. 00-86-028-85503486

^‡^These authors contributed equally to this work

**Author contributions**

Zhiwu Wu: collection and/or assembly of data, data analysis and interpretation, and manuscript writing. Hui Yang: data analysis, interpretation, financial support, and manuscript writing.

Shaoying Duan: collection and/or assembly of data, data analysis and interpretation.

Qianqian Su: data analysis and interpretation.

Ran Cheng: conception and design, data analysis and interpretation, financial surport, and manuscript writing.

Tao Hu: conception and design, data analysis and interpretation, financial surport, and manuscript writing.

**Acknowlegements**

The authors would like to express our heartfelt gratitude to the National Natural Science Foundation of China (U20A20365 and 81970948) and Sichuan Science and Technology Program (2024NSFSC0543 and 2024YFHZ0043) for funding supports. The schematic diagram in figures were created with BioRender.com.

**Keywords:** H3K9ac, H3K27ac, NRG1, Pulp regeneration, SAHA

**Supplemental materials**

1. **Supplemental materials and methods**

**Clinical specimens and isolation of human dental pulp stem cells (hDPSCs)**

Human normal teeth, as well as teeth affected by caries and pulpitis, were procured from patients at West China Hospital of Stomatology, Sichuan University, with informed consent obtained from the patients or their families. Inclusion Criteria: The teeth with a diagnosis of dental caries or chronic pulpitis. Exclusion Criteria: Patients with systemic diseases or those who had taken antibiotics or non-steroidal anti-inflammatory drugs within the past three months. Normal teeth were collected from patients that premolar for orthodontic purposes and third molar teeth that needed to be extracted. Both affected and normal teeth were rinsed with saline.

Pulp tissues were derived from normal teeth of patients aged 18-25 years and then digested in 3 mg/mL collagenase type I (HyClone, USA) for 1 h at 37°C. DPSCs were cultured in Dulbecco’s modified Eagle’s medium (DMEM, Gibco, USA) with 10% fetal bovine serum (Gibco) and 1% penicillin/streptomycin (HyClone) in 5% CO_2_ incubator. 3-6 generation hDPSCs were used in this study. Flow cytometry was used to characterize stemness of hDPSCs. ALP staining and alizarin red S staining, oil red O staining, and saffron O staining were used to verify the differentiatial capacity of hDPSCs.

**Lentivirus mediated *NRG1* gene knockdown/overexpression**

A total of three NRG1-shRNAs were designed and screened to identify the shRNA with the best knockdown effect for subsequent studies. The NRG1-shRNA lentiviral vector was GV493, and the detailed information is shown in the Appendix. Lentiviral packaging of NRG1 overexpression plasmid was utilized, and then DPSCs were infected to obtain cell lines capable of stably overexpressing NRG1. The NRG1 overexpression lentiviral vector was GV492, the cloning site: BamHI-/-AgeI, and the viral titer was 5×10^8^. The lentiviral re-infection index MOI was 10, and the transfection enhancer A was selected, while LV-NRG1- RNAi (98709-1) for the best knockdown effect. Follow-up experiments were performed. 2×10^4^ cells per well were seeded in 6-well plates and cultured for 1 day. Remove the original medium in the well plate and add fresh medium containing 1mL of lentivirus and A-enhancer per well, and calculate the required volume of lentivirus according to MOI, cell number and lentiviral titer. Replace with 2 mL of fresh medium after 16 h. Fluorescence microscopy was performed to observe the infection efficiency after 3 days of infection, puromycin (3 μg/mL) was used to screen the stable cell lines, and some of the cells were collected for the validation of transfection effect.

**Flow cytometry**

hDPSCs from generation 3 were harvested and subjected to two washes with PBS via resuspension, followed by aspiration of the supernatant. Subsequently, 100 μL of PBS was utilized for cell resuspension, and the requisite antibodies (CD29, CD34, CD44, CD90, and CD105) were added in accordance with the protocol. The cells were incubated for 30 minutes at 4°C in the absence of light, then centrifuged at 1500 rpm for 5 minutes. The supernatant was discarded, and the cells underwent two additional washes with 1 mL of PBS. After another centrifugation at 1500 rpm for 5 minutes, the supernatant was aspirated, and the cells were resuspended in 500 μL of PBS for flow cytometry analysis (Beckman Cytoflex, USA).

**Micro-computed tomography**

Maxillary samples of mineralization model from day 7 and 28 were subjected to scanning using the Scanco μCT45 (Acanco Medical, Scanco Medical, Switzerland). The scanning parameters included an accuracy of 10 μm, 55 kVp, 145 μA, and an exposure duration of 250 ms. The analyzed region was the crown of the maxillary first molar. Dentin formation within the pulp cavity was quantified through bone mineral density (BMD), bone volume/total volume (BV/TV), trabecular number (Tb.N), trabecular thickness (Tb.Th), and trabecular spacing (Tb.Sp). Elevated values of BMD, BV/TV, Tb.N, and Tb.Th, alongside reduced values of Tb.Sp, signify increased dentin formation in the pulp cavity. Dentin formation in the pulp cavity of rats was observed via μCT cross-sections.

**Hematoxylin and eosin (H&E) staining**

The procedure was conducted in accordance with the H&E staining kit (G1120, Solarbio, China). The slices were dewaxed and hydrated. Subsequently, the nucleus and cytoplasm were stained with haematoxylin and eosin, respectively, and the slices were dehydrated, made transparent, sealed, and observed under a microscope (Leica DM2000, Leica Corporation, Wetzlar, Germany).

**Immunofluorescence staining and multiplex immunohistochemical (mIHC)**

Tris-EDTA solution was employed for antigen retrieval and 5% BSA solution were applied for antigen blocking for 30 minutes at 37°C. Subsequently, primary antibodies (NRG1, H3K9ac, H3K27ac) were applied. The following day, secondary antibody was incubated at 37°C for 30 minutes. The nuclei were visualized using DAPI solution at room temperature for 10 minutes. Images were captured using a microscope (Olympus BX53, Japan).

The procedures for mIHC were conducted in accordance with the Multiplex Fluorescence Immunohistochemical Staining Kit (abs50012, Absin, China). The slices were incubated in 5% BSA solution at room temperature for 30 minutes. The first primary antibody was applied overnight incubation at 4°C. Then the slices were incubated in secondary antibody for 10 minutes at room temperature. The fluorescence staining working solution was then incubated 10-minute incubation at room temperature. DAPI solution was applied for staining at room temperature for 10 minutes.

**Immunohistochemistry staining**

The procedures were performed according to the immunohistochemical staining kit (E-IR-R217, Elabscience Biotechnology Co., LTD, China). After goat serum blocking solution for 30 minutes at 37℃, the slides were incubated overnight with the primary antibodies (NRG1, H3K9ac, H3K27ac, IL-1β, IL-6, IL-8, TNF-α, DSPP, DMP1, Nestin, NF200, CD31) at 4℃, followed by incubation with an anti- rabbit/mouse secondary antibody for 30 min at 37°C. Images were acquired using a microscope (Leica DM2000). Relative positive expression (integrated optical density (IOD)/area) was calculated using Image- Pro Plus 6.0 software (Media Cybernetics, Inc., USA).

**Real-time quantitative polymerase chain reaction (qPCR)**

The hDPSCs were seeded at a density of 1 × 10^6^ cells per well in 6-well plates, followed by treated with LPS for 1day and 3 days or induced OD for 3 and 7day. In addition, the sh/oe-NRG1 hDPSCs was treated with LPS and the hDPSCs was stimulated with LPS and SAHA/C646. mRNA was extracted after using the Animal RNA Extraction Kit (R0026, Beyotime, China). Reverse transcription was conducted using the Reverse Transcription Kit (11141ES60, Yeason, China). The cDNA was acquired and preserved at -20°C. Real-time quantitative polymerase chain reaction (qPCR) was conducted utilizing TB Green® Premix Ex Taq™ II FAST qPCR (CN830S Takara, Japan). The result was normalized to controls and presented as relative expression.

| Gene | Forward primers | Revese primer |
| --- | --- | --- |
| *NRG1* | ATGGAGGCGGAGGAGCTGTA | TTGCAGTAGGCCACCACACA |
| *IL-1β* | TTATTACAGTGGCAATGAGGATGAC | CCTGAAGCCCTTGCTGTAGTG |
| *IL-6* | ACTCACCTCTTCAGAACGAATTG | CCATCTTTGGAAGGTTCAGGTTG |
| *IL-8* | AAGAAACCACCGGAAGGAAC | ACTCCTTGGCAAAACTGCAC |
| *TNF-α* | CGTGGAGCTGGCCGAGGAG | AGGAAGGAGAAGAGGCTGAGGAAC |

**Western blotting**

The hDPSCs were seeded at a density of 1 × 10^6^ cells per well in 6-well plates, followed by treated with LPS for 3 hours, 1day and 2 days or induced OD for 3 days,7days and 14 day. The hDPSCs were seeded at a density of 1 × 10^6^ cells per well in 6-well plates, followed by treated with LPS/mineralization induced medium (MIM) and SAHA/C646. The sh/oe-NRG1 hDPSCs were treated with LPS/ MIM.The protein was extracted according to the Total Protein Extraction Kit (PE001, Signalway Antibody, USA). Equal amounts of protein were separated using SDS–PAGE (ET15420gel, ACE Biotechnology, China) and transferred to polyvinylidene difluoride membranes (GE Healthcare Life Science, USA). The membrane was incubated 5% BSA solution for 1 hour at room temperature and then incubated with the primary antibodies (NRG1, H3K9ac, H3K27ac, IL-1β, IL-6, IL-8, TNF-α, DSPP, DMP1, β-Tubulin) overnight at 4 °C. The next day, the secondary antibody was incubated for 1 hour at room temperature. After incubation with exposure solution for a few seconds, images were taken on a gel imaging system (ChemiDoc imaging system Bio-Rad, USA).

**Enzyme-linked immunosorbent assay (ELISA)**

hDPSCs seeded and intervened as explained in the paragraph “Western blot”. IL-1β, IL-6,IL-8 and TNF-α levels in the culture supernatants were measured using ELISA kit according to the manufacturer's instructions. Details are as follows:

| Target | Details |
| --- | --- |
| IL-1β | Human, abs510002, Absin, China |
| IL-1β | Human, EH0006, Youke Life Sciences Technology CO.,LTD, China |
| IL-6 | Human, abs510003, Absin, China |
| IL-8 | Human, E-EL-H6008, Elabscience Biotechnology Co., LTD, China |
| TNF-α | Human, abs510006, Absin, China |
| TNF-α | Human, EH0002, Youke Life Sciences Technology CO.,LTD, China |

**Alkaline phosphatase (ALP) staining and activity assay**

The hDPSCs were seeded at a density of 1 × 10^6^ cells per well in 6-well plates, followed by induced OD with/without SAHA and C646 for 7 days. ALP staining was conducted in accordance with the BCIP/NBT alkaline phosphatase chromogenic kit (C3206, Beyotime, China). The BCIP/NBT staining working solution was introduced, and the reaction was halted by incubating for 5 minutes at room temperature. Finally, the cells were examined using a microscope (Olympus 1X7, Japan).

ALP activity assay was conducted in accordance with AKP/ALP test kit (A059-2, Jiancheng Bioengineering Institute, China) and normalized with total protein loadings. Absorbance values were measured using an enzyme labeler (Molecular Devices SpectraMax iD3, USA) at 520 nm.

**Alizarin red S staining and measurement of calcium concentration**

The hDPSCs were seeded at a density of 1 × 10^6^ cells per well in 6-well plates, followed by induced OD with/without SAHA and C646. The hDPSCs was incubated in Alizarin Red S staining solution (G1450, Solarbio, China) for 30 minutes at room temperature and examine the chromatograms using microscopy (Olympus 1X7, Japan) to observe calcium deposition by ARS staining. Mineralized bone nodules were destained with 10% cetylpyridinium chloride in double distilled water, and the calcium concentration was was measured at 570 nm by microplate reader (Molecular Devices SpectraMax iD3, USA).

**Long non-coding RNA sequencing (lncRNA-seq)**

Library Construction, Quality Control and Sequencing: Total RNA was used as input material for the RNA sample preparations. Sequencing libraries were generated using RNA Library Prep Kit following manufacturer’s recommendations and index codes were added to attribute sequences to each sample. Briefly, regulatory ncRNA and mRNA were purified from total RNA using probes to remove rRNA. Fragmentation was carried out using divalent cations under elevated temperature in First Strand Synthesis Reaction Buffer(5X). First strand cDNA was synthesized using random hexamer primer and M-MuLV Reverse Transcriptase (RNaseH). Second strand cDNA synthesis was subsequently performed using DNA Polymerase I and RNase H. Remaining overhangs were converted into blunt ends via exonuclease/polymerase activities. After adenylation of 3’ ends of DNA fragments, NEB Next Adaptor with hairpin loop structure were ligated to prepare for hybridization. In order to select cDNA fragments of preferentially 370~420 bp in length, the library fragments were purified with AMPure XP system. Then 3 µL USER Enzyme was used with size-selected, adaptor-ligated cDNA at 37°C for 15 min followed by 5 min at 95 °C before PCR. Then PCR was performed with Phusion High-Fidelity DNA polymerase, Universal PCR primers and Index Primer. At last, PCR products were purified library quality was assessed on the Agilent 5400 system and quantified by QPCR (1.5 nM). The Qualified libraries were pooled and sequenced on Illumina platforms with PE150 strategy in Novogene Bioinformatics Technology Co., Ltd (Beijing, China), according to effective library concentration and data amount required.

Bioinformatics Analysis Pipeline: The original fluorescence image files obtained from Illumina platform are transformed to short reads (Raw data) by base calling and these short reads are recorded in FASTQ format, which contains sequence information and corresponding sequencing quality information. Sequence artifacts, including reads containing adapter contamination, low quality nucleotides and unrecognizable nucleotide (N), undoubtedly set the barrier for the subsequent reliable bioinformatics analysis. Hence quality control is an essential step and applied to guarantee the meaningful downstream analysis. we used Fastp (version 0.23.1) (Chen et al., 2018) to perform basic statistics on the quality of the raw reads. The steps of data processing were as follows: (1) Discard a paired reads if either one read contains adapter contamination; (2) Discard a paired reads if more than 10% of bases are uncertain in either one read; (3) Discard a paired reads if the proportion of low quality (Phred quality <5) bases is over 50% in either one read.

**The assay for transposase-accessible chromatin using sequencing (ATAC-seq)**

Sample collection and preparation: Take out the cryovial and shake it in 37℃ water to quickly thaw it within 1-2 min. hDPSCs were centrifuged at 4 ℃ 500×g for 5 min. hDPSCs activity was detected with LUNA-FL and counted.

50000 live hDPSCs were centrifuged with 500 g for 5 minutes, supernatant was discarded, the precipitation was resuspended with 50 μL lysis buffer, and placed on ice for 5 minutes, then 1mL wash buffer was added, mixed, 500 g centrifuged for 5 minutes, supernatant was discarded, and nucleus was collected. Taking 50000 nucleus, adding 1 mL washing buffer, mixing them well, ang then centrifuged 500 g for 5 minutes, discarding supernatant, transposing finally.

Library construction for ATAC-seq: ATAC-seq was performed as previously reported. Briefly, nucleus was extracted from samples, and the nucleus pellet was resuspended in the Tn5 transposase reaction mix. The transposition reaction was incubated at 37℃ for 30 min. Equimolar Adapter1 and Adatper 2 were added after transposition, PCR was then performed to amplify the library (ATAC-seq_Novogene Kit). After the PCR reaction, libraries were purified with the AMPure beads and library quality was assessed with Qubit.

Sequencing: The clustering of the index-coded samples was performed on a cBot Cluster Generation System using TruSeq PE Cluster Kit v3-cBot-HS (Illumina) according to the manufacturer’s instructions. The library preparations were sequenced on Illumina Novaseq platform at Tianjin Novogene Bioinformatic Technology Co., Ltd (Beijing, China) and 150 bp paired-end reads were generated.

Quality control for raw data: Raw data (raw reads) of fastq format were firstly processed using fastp (v 0.20.0). In this step, clean data (clean reads) were obtained by removing reads containing adapter, reads containing ploy-N and low-quality reads (reads with a mass value of less than 15 bases more than 40% of the number of bases of the reads and pruned reads shorter than 18bp) from raw data. At the same time, Q20, Q30 and GC content of the clean data were calculated. All the downstream analyses were based on the clean data.

Mapping: Reference genome and gene annotation files were downloaded from genome website directly. Index of the reference genome was built using BWA (v 0.7.12) and clean reads were aligned to the reference genome using BWA mem. Reads that were derived from mitochondrion DNA and chloroplast DNA were discarded. These reads were then filtered for high quality (MAPQ ≥ 13),we also removed reads that were not properly paired and with PCR duplicates. Only uniquely mapped(MAPQ ≥ 13) and de-duplicated reads were used for further analysis.

Peak calling: All peak calling was performed with MACS2 (v 2.2.7.1) using ‘macs2 -q 0.05 --call-summits --nomodel --shift -100 --extsize 200 --keep-dup all’. By default, peaks with q-value threshold of 0.05 was used for all data sets.

Motif analysis: Peaks were adjusted to the same size (500 bp) centered peak summits and motif discoveries of these loci sequences were performed using findMotifsGenome.pl program in HOMER (v4.11) checksoftware with ‘-len 8,10,12,14 -gc -size given -p 2 -S 25 -homer2 -dumpFasta’.

Peak annotation: The position of peak summit around transcript start sites of genes can predict the interaction sites between protein and gene. ChIPseeker was used to retrieve the nearest genes around the peak and annotate genomic region of the peak. Peak-related genes can be confirmed by ChIPseeker, and then Gene Ontology (GO) enrichment analysis was performed to identify the function enrichment results. GO enrichment analysis was implemented by the GOseq R package, in which gene length bias was corrected. GO terms with corrected P-value less than 0.05 were considered significantly enriched by peak-related genes. KEGG is a database resource for understanding high-level functions and utilities of the biological system, such as the cell, the organism and the ecosystem, from molecular-level information, especially large-scale molecular datasets generated by genome sequencing and other high-throughput experimental technologies (http://www.genome.jp/kegg/). We used KOBAS software to test the statistical enrichment of peak related genes in KEGG pathways.

Different peak analysis: Peaks of different groups were merged using ‘bedtools merge’.We caltulated the mean RPM of each group in the merge peak. Only peaks with fold change of RPM more than 2（|log2fc |>1）were considered as differential peaks. Genes associated with different peaks were identified using ChIPseeker.

**Preparation of human-treated dentin matrix (TDM)**

Premolars extracted for orthodontic purposes or intact third molars were used to prepare human-treated dentin matrix (TDM). The roots were sectioned into 3-5 mm segments. Osteoid and dentin were partially abraded with a handpiece. The root fragments underwent ultrasonic cleaning, followed by treatment with EDTA (17%, 10% and 5%) for 20 minutes. The fragments were immersed in iodophor for 30 minutes, followed by three PBS washes of 5 minutes each. They were treated with 5.25% sodium hypochlorite for 10-15 minutes ultimately.

1. **Table S1 (Antibodies)**

| Antibodies | Catalog Number | Supplier | Application |
| --- | --- | --- | --- |
| rabbit anti-NRG1 | 10527-1-AP | Proteintech, USA | IF, mIHC |
| rabbit anti-H3K9ac | YK0006 | Immonoway, USA | IF, mIHC |
| mouse anti-H3K27ac | HA500046 | Huabio Hangzhou, China | IF, mIHC |
| rabbit anti-IL-1β | TA 5103 | Abmart, China | IHC |
| rabbit anti-IL-6 | R1412-2 | Huabio, China | IHC |
| rabbit anti-IL-8 | R1511-15 | Huabio, China | IHC |
| rabbit anti-TNF-α | ab6671 | Abcam, England | IHC |
| mouse anti-DSPP | sc-73632 | Santa Cruz, USA | IHC, mIHC |
| mouse anti-DMP1 | sc73633 | Santa Cruz, USA | IHC |
| rabbit anti-Nestin | 19483-1-AP | Proteintech, USA | IHC, mIHC |
| rabbit anti-NF200 | ET1702-72 | Huabio, Hangzhou, China | IHC |
| rabbit anti-CD31 | ab28369 | Abcam, England | IHC |
| mouse anti-NRG1 | sc-393006 | Santa Cruz, USA | WB |
| rabbit anti-H3K9ac | YK0006 | Immonoway, USA | WB |
| mouse anti-H3K27ac | HA500046 | Huabio, Hangzhou, China | WB |
| rabbit anti-IL-1β | TA5103 | Abmart, China | WB |
| rabbit anti-IL-6 | R1412-2 | Huabio, China | WB |
| rabbit anti-IL-8 | R1511-15 | Huabio, China | WB |
| rabbit anti-TNF-α | ab6671 | Abcam, England | WB |
| mouse anti-DSPP | sc-73632 | Santa Cruz, USA | WB |
| mouse anti-DMP1 | sc73633 | Santa Cruz, USA | WB |
| mouse anti-β-tubulin | 44032 | Signalway Antibody, USA | WB |
| rabbit anti-conecexin43 | 26980-1-AP | Proteintech, USA | mIHC |
| rabbit anti-H3K9ac ChIP grade | ab32129 | Abcam, England | ChIP |
| rabbit anti-H3K27ac ChIP grade | ab4729 | Abcam, England | ChIP |
| mouse anti-CD29-APC | 17-0299-41 | Thermo Fisher, USA | Flow cytometry |
| mouse anti-CD34-FITC | 343603 | Biolegend, USA | Flow cytometry |
| mouse anti-CD44-FITC | 338803 | Biolegend, USA | Flow cytometry |
| mouse anti-CD45-PE | 368509 | Biolegend, USA | Flow cytometry |
| mouse anti-CD90-APC | 17-0909-41 | Thermo Fisher, USA | Flow cytometry |
| mouse anti-CD105-APC | 17-1057-41 | Thermo Fisher, USA | Flow cytometry |
| 488 goat anti-mouse IgG | A11011 | Invitrogen, Carlsbad, CA, USA | IF |
| 488 goat anti-rabbit IgG | A11008 | Invitrogen, Carlsbad, CA, USA | IF |
| goat anti-mouse IgG | L3032 | Signalway Antibody, USA | WB |
| goat anti-rabbit IgG | (L3012 | Signalway Antibody, USA | WB |

1. Table S2. The functions of gene/protein were collected by UniproKB (<https://www.uniprot.org/>)

| Gene symbol | Functions |
| --- | --- |
| TGFB1 | Multifunctional protein that regulates the growth and differentiation of various cell types and is involved in various processes, such as normal development, immune function, microglia function and responses to neurodegeneration .  Activation into mature form follows different steps: following cleavage of the proprotein in the Golgi apparatus, Latency-associated peptide (LAP) and Transforming growth factor beta-1 (TGF-beta-1) chains remain non-covalently linked rendering TGF-beta-1 inactive during storage in extracellular matrix.  At the same time, LAP chain interacts with 'milieu molecules', such as LTBP1, LRRC32/GARP and LRRC33/NRROS that control activation of TGF-beta-1 and maintain it in a latent state during storage in extracellular milieus.  TGF-beta-1 is released from LAP by integrins (ITGAV:ITGB6 or ITGAV:ITGB8): integrin-binding to LAP stabilizes an alternative conformation of the LAP bowtie tail and results in distortion of the LAP chain and subsequent release of the active TGF-beta-1.  Once activated following release of LAP, TGF-beta-1 acts by binding to TGF-beta receptors (TGFBR1 and TGFBR2), which transduce signal.  While expressed by many cells types, TGF-beta-1 only has a very localized range of action within cell environment thanks to fine regulation of its activation by Latency-associated peptide chain (LAP) and 'milieu molecules.  Plays an important role in bone remodeling: acts as a potent stimulator of osteoblastic bone formation, causing chemotaxis, proliferation and differentiation in committed osteoblasts.  Can promote either T-helper 17 cells (Th17) or regulatory T-cells (Treg) lineage differentiation in a concentration-dependent manner.  At high concentrations, leads to FOXP3-mediated suppression of RORC and down-regulation of IL-17 expression, favoring Treg cell development.  At low concentrations in concert with IL-6 and IL-21, leads to expression of the IL-17 and IL-23 receptors, favoring differentiation to Th17 cells.  Stimulates sustained production of collagen through the activation of CREB3L1 by regulated intramembrane proteolysis (RIP).  Mediates SMAD2/3 activation by inducing its phosphorylation and subsequent translocation to the nucleus.  Positively regulates odontoblastic differentiation in dental papilla cells, via promotion of IPO7-mediated translocation of phosphorylated SMAD2 to the nucleus and subsequent transcription of target genes.  Can induce epithelial-to-mesenchymal transition (EMT) and cell migration in various cell types. |
| TNF | Cytokine that binds to TNFRSF1A/TNFR1 and TNFRSF1B/TNFBR. It is mainly secreted by macrophages and can induce cell death of certain tumor cell lines. It is potent pyrogen causing fever by direct action or by stimulation of interleukin-1 secretion and is implicated in the induction of cachexia, Under certain conditions it can stimulate cell proliferation and induce cell differentiation. Impairs regulatory T-cells (Treg) function in individuals with rheumatoid arthritis via FOXP3 dephosphorylation. Up-regulates the expression of protein phosphatase 1 (PP1), which dephosphorylates the key 'Ser-418' residue of FOXP3, thereby inactivating FOXP3 and rendering Treg cells functionally defective (PubMed:23396208). Key mediator of cell death in the anticancer action of BCG-stimulated neutrophils in combination with DIABLO/SMAC mimetic in the RT4v6 bladder cancer cell line. Induces insulin resistance in adipocytes via inhibition of insulin-induced IRS1 tyrosine phosphorylation and insulin-induced glucose uptake. Induces GKAP42 protein degradation in adipocytes which is partially responsible for TNF-induced insulin resistance. Plays a role in angiogenesis by inducing VEGF production synergistically with IL1B and IL6. Promotes osteoclastogenesis and therefore mediates bone resorption.  The TNF intracellular domain (ICD) form induces IL12 production in dendritic cells. |
| BMP2 | Growth factor of the TGF-beta superfamily that plays essential roles in many developmental processes, including cardiogenesis, neurogenesis, and osteogenesis.  Induces cartilage and bone formation.  Initiates the canonical BMP signaling cascade by associating with type I receptor BMPR1A and type II receptor BMPR2.  Once all three components are bound together in a complex at the cell surface, BMPR2 phosphorylates and activates BMPR1.  In turn, BMPR1A propagates signal by phosphorylating SMAD1/5/8 that travel to the nucleus and act as activators and repressors of transcription of target genes. Also acts to promote expression of HAMP, via the interaction with its receptor BMPR1A/ALK3.  Can also signal through non-canonical pathways such as ERK/MAP kinase signaling cascade that regulates osteoblast differentiation.  Also stimulates the differentiation of myoblasts into osteoblasts via the EIF2AK3-EIF2A-ATF4 pathway by stimulating EIF2A phosphorylation which leads to increased expression of ATF4 which plays a central role in osteoblast differentiation.  Acts as a positive regulator of odontoblast differentiation during mesenchymal tooth germ formation, expression is repressed during the bell stage by MSX1-mediated inhibition of CTNNB1 signaling. |
| FGF2 | Acts as a ligand for FGFR1, FGFR2, FGFR3 and FGFR4. Also acts as an integrin ligand which is required for FGF2 signalin. Binds to integrin ITGAV:ITGB3 . Plays an important role in the regulation of cell survival, cell division, cell differentiation and cell migration. Functions as a potent mitogen in vitro. Can induce angiogenesis. Mediates phosphorylation of ERK1/2 and thereby promotes retinal lens fiber differentiation. |
| MAPK1 | Serine/threonine kinase which acts as an essential component of the MAP kinase signal transduction pathway. MAPK1/ERK2 and MAPK3/ERK1 are the 2 MAPKs which play an important role in the MAPK/ERK cascade. They participate also in a signaling cascade initiated by activated KIT and KITLG/SCF. Depending on the cellular context, the MAPK/ERK cascade mediates diverse biological functions such as cell growth, adhesion, survival and differentiation through the regulation of transcription, translation, cytoskeletal rearrangements. The MAPK/ERK cascade also plays a role in initiation and regulation of meiosis, mitosis, and postmitotic functions in differentiated cells by phosphorylating a number of transcription factors. About 160 substrates have already been discovered for ERKs. Many of these substrates are localized in the nucleus, and seem to participate in the regulation of transcription upon stimulation. However, other substrates are found in the cytosol as well as in other cellular organelles, and those are responsible for processes such as translation, mitosis and apoptosis. Moreover, the MAPK/ERK cascade is also involved in the regulation of the endosomal dynamics, including lysosome processing and endosome cycling through the perinuclear recycling compartment (PNRC); as well as in the fragmentation of the Golgi apparatus during mitosis. The substrates include transcription factors (such as ATF2, BCL6, ELK1, ERF, FOS, HSF4 or SPZ1), cytoskeletal elements (such as CANX, CTTN, GJA1, MAP2, MAPT, PXN, SORBS3 or STMN1), regulators of apoptosis (such as BAD, BTG2, CASP9, DAPK1, IER3, MCL1 or PPARG), regulators of translation (such as EI[F4EBP1](https://www.uniprot.org/uniprotkb/F4EBP1) and FXR1) and a variety of other signaling-related molecules (like ARHGEF2, DCC, FRS2 or GRB10). Protein kinases (such as RAF1, RPS6KA1/RSK1, RPS6KA3/RSK2, RPS6KA2/RSK3, RPS6KA6/RSK4, SYK, MKNK1/MNK1, MKNK2/MNK2, RPS6KA5/MSK1, RPS6KA4/MSK2, MAPKAPK3 or MAPKAPK5) and phosphatases (such as DUSP1, DUSP4, DUSP6 or DUSP16) are other substrates which enable the propagation the MAPK/ERK signal to additional cytosolic and nuclear targets, thereby extending the specificity of the cascade. Mediates phosphorylation of TPR in response to EGF stimulation. May play a role in the spindle assembly checkpoint. Phosphorylates PML and promotes its interaction with PIN1, leading to PML degradation. Phosphorylates CDK2AP2. Acts as a transcriptional repressor. Binds to a [GC]AAA[GC] consensus sequence. Repress the expression of interferon gamma-induced genes. Seems to bind to the promoter of CCL5, DMP1, IFIH1, IFITM1, IRF7, IRF9, LAMP3, OAS1, OAS2, OAS3 and STAT1. Transcriptional activity is independent of kinase activity. |
| VEGFA | Growth factor active in angiogenesis, vasculogenesis and endothelial cell growth.  Induces endothelial cell proliferation, promotes cell migration, inhibits apoptosis and induces permeabilization of blood vessels. Binds to the FLT1/VEGFR1 and KDR/VEGFR2 receptors, heparan sulfate and heparin. Binds to the NRP1/neuropilin-1 receptor. Binding to NRP1 initiates a signaling pathway needed for motor neuron axon guidance and cell body migration, including for the caudal migration of facial motor neurons from rhombomere 4 to rhombomere 6 during embryonic development.  Also binds the DEAR/FBXW7-AS1 receptor. |
| EGFR | Receptor tyrosine kinase binding ligands of the EGF family and activating several signaling cascades to convert extracellular cues into appropriate cellular responses.  Known ligands include EGF, TGFA/TGF-alpha, AREG, epigen/EPGN, BTC/betacellulin, epiregulin/EREG and HBEGF/heparin-binding EGF.  Ligand binding triggers receptor homo- and/or heterodimerization and autophosphorylation on key cytoplasmic residues. The phosphorylated receptor recruits adapter proteins like GRB2 which in turn activates complex downstream signaling cascades. Activates at least 4 major downstream signaling cascades including the RAS-RAF-MEK-ERK, PI3 kinase-AKT, PLCgamma-PKC and STATs modules.  May also activate the NF-kappa-B signaling cascade.  Also directly phosphorylates other proteins like RGS16, activating its GTPase activity and probably coupling the EGF receptor signaling to the G protein-coupled receptor signaling.  Also phosphorylates MUC1 and increases its interaction with SRC and CTNNB1/beta-catenin.  Positively regulates cell migration via interaction with CCDC88A/GIV which retains EGFR at the cell membrane following ligand stimulation, promoting EGFR signaling which triggers cell migration.  Plays a role in enhancing learning and memory performance.  Plays a role in mammalian pain signaling (long-lasting hypersensitivity) |
| BMP4 | Growth factor of the TGF-beta superfamily that plays essential roles in many developmental processes, including neurogenesis, vascular development, angiogenesis and osteogenesis. Acts in concert with PTHLH/PTHRP to stimulate ductal outgrowth during embryonic mammary development and to inhibit hair follicle induction. Initiates the canonical BMP signaling cascade by associating with type I receptor BMPR1A and type II receptor BMPR2. Once all three components are bound together in a complex at the cell surface, BMPR2 phosphorylates and activates BMPR1A. In turn, BMPR1A propagates signal by phosphorylating SMAD1/5/8 that travel to the nucleus and act as activators and repressors of transcription of target genes. Positively regulates the expression of odontogenic development regulator MSX1 via inducing the IPO7-mediated import of SMAD1 to the nucleus. Required for MSX1-mediated mesenchymal molar tooth bud development beyond the bud stage, via promoting Wnt signaling (By similarity). Acts as a positive regulator of odontoblast differentiation during mesenchymal tooth germ formation, expression is repressed during the bell stage by MSX1-mediated inhibition of CTNNB1 signaling. Able to induce its own expression in dental mesenchymal cells and also in the neighboring dental epithelial cells via an MSX1-mediated pathway. Can also signal through non-canonical BMP pathways such as ERK/MAP kinase, PI3K/Akt, or SRC cascades. For example, induces SRC phosphorylation which, in turn, activates VEGFR2, leading to an angiogenic response |
| EGF | EGF stimulates the growth of various epidermal and epithelial tissues in vivo and in vitro and of some fibroblasts in cell culture. Magnesiotropic hormone that stimulates magnesium reabsorption in the renal distal convoluted tubule via engagement of EGFR and activation of the magnesium channel TRPM6. Can induce neurite outgrowth in motoneurons of the pond snail Lymnaea stagnalis in vitro |
| TNFSF11 | Cytokine that binds to TNFRSF11B/OPG and to TNFRSF11A/RANK. Osteoclast differentiation and activation factor. Augments the ability of dendritic cells to stimulate naive T-cell proliferation. May be an important regulator of interactions between T-cells and dendritic cells and may play a role in the regulation of the T-cell-dependent immune response. May also play an important role in enhanced bone-resorption in humoral hypercalcemia of malignancy.  Induces osteoclastogenesis by activating multiple signaling pathways in osteoclast precursor cells, chief among which is induction of long lasting oscillations in the intracellular concentration of Ca 2+ resulting in the activation of NFATC1, which translocates to the nucleus and induces osteoclast-specific gene transcription to allow differentiation of osteoclasts. During osteoclast differentiation, in a TMEM64 and ATP2A2-dependent manner induces activation of CREB1 and mitochondrial ROS generation necessary for proper osteoclast generation |
| HGF | Potent mitogen for mature parenchymal hepatocyte cells, seems to be a hepatotrophic factor, and acts as a growth factor for a broad spectrum of tissues and cell types.  Activating ligand for the receptor tyrosine kinase MET by binding to it and promoting its dimerization.  Activates MAPK signaling following TMPRSS13 cleavage and activation |
| SRC | Non-receptor protein tyrosine kinase which is activated following engagement of many different classes of cellular receptors including immune response receptors, integrins and other adhesion receptors, receptor protein tyrosine kinases, G protein-coupled receptors as well as cytokine receptors. Participates in signaling pathways that control a diverse spectrum of biological activities including gene transcription, immune response, cell adhesion, cell cycle progression, apoptosis, migration, and transformation. Due to functional redundancy between members of the SRC kinase family, identification of the specific role of each SRC kinase is very difficult. SRC appears to be one of the primary kinases activated following engagement of receptors and plays a role in the activation of other protein tyrosine kinase (PTK) families. Receptor clustering or dimerization leads to recruitment of SRC to the receptor complexes where it phosphorylates the tyrosine residues within the receptor cytoplasmic domains. Plays an important role in the regulation of cytoskeletal organization through phosphorylation of specific substrates such as AFAP1. Phosphorylation of AFAP1 allows the SRC SH2 domain to bind AFAP1 and to localize to actin filaments. Cytoskeletal reorganization is also controlled through the phosphorylation of cortactin (CTTN) (Probable). When cells adhere via focal adhesions to the extracellular matrix, signals are transmitted by integrins into the cell resulting in tyrosine phosphorylation of a number of focal adhesion proteins, including PTK2/FAK1 and paxillin (PXN).  In addition to phosphorylating focal adhesion proteins, SRC is also active at the sites of cell-cell contact adherens junctions and phosphorylates substrates such as beta-catenin (CTNNB1), delta-catenin (CTNND1), and plakoglobin (JUP). Another type of cell-cell junction, the gap junction, is also a target for SRC, which phosphorylates connexin-43 (GJA1). SRC is implicated in regulation of pre-mRNA-processing and phosphorylates RNA-binding proteins such as KHDRBS1 (Probable). Phosphorylates PKP3 at 'Tyr-195' in response to reactive oxygen species, which may cause the release of PKP3 from desmosome cell junctions into the cytoplasm.  Also plays a role in PDGF-mediated tyrosine phosphorylation of both STAT1 and STAT3, leading to increased DNA binding activity of these transcription factors.  Involved in the RAS pathway through phosphorylation of RASA1 and RASGRF1.  Plays a role in EGF-mediated calcium-activated chloride channel activation.  Required for epidermal growth factor receptor (EGFR) internalization through phosphorylation of clathrin heavy chain (CLTC and CLTCL1) at 'Tyr-1477'. Involved in beta-arrestin (ARRB1 and ARRB2) desensitization through phosphorylation and activation of GRK2, leading to beta-arrestin phosphorylation and internalization. Has a critical role in the stimulation of the CDK20/MAPK3 mitogen-activated protein kinase cascade by epidermal growth factor (Probable). Might be involved not only in mediating the transduction of mitogenic signals at the level of the plasma membrane but also in controlling progression through the cell cycle via interaction with regulatory proteins in the nucleus.  Plays an important role in osteoclastic bone resorption in conjunction with PTK2B/PYK2. Both the formation of a SRC-PTK2B/PYK2 complex and SRC kinase activity are necessary for this function. Recruited to activated integrins by PTK2B/PYK2, thereby phosphorylating CBL, which in turn induces the activation and recruitment of phosphatidylinositol 3-kinase to the cell membrane in a signaling pathway that is critical for osteoclast function.  Promotes energy production in osteoclasts by activating mitochondrial cytochrome C oxidase.  Phosphorylates DDR2 on tyrosine residues, thereby promoting its subsequent autophosphorylation.  Phosphorylates RUNX3 and COX2 on tyrosine residues, TNK2 on 'Tyr-284' and CBL on 'Tyr-731.  Enhances RIGI-elicited antiviral signaling.  Phosphorylates PDPK1 at 'Tyr-9', 'Tyr-373' and 'Tyr-376'.  Phosphorylates BCAR1 at 'Tyr-128'.  Phosphorylates CBLC at multiple tyrosine residues, phosphorylation at 'Tyr-341' activates CBLC E3 activity (PubMed:20525694).  Phosphorylates synaptic vesicle protein synaptophysin (SYP).  Involved in anchorage-independent cell growth.  Required for podosome formation.  Mediates IL6 signaling by activating YAP1-NOTCH pathway to induce inflammation-induced epithelial regeneration.  Phosphorylates OTUB1, promoting deubiquitination of RPTOR.  Phosphorylates caspase CASP8 at 'Tyr-380' which negatively regulates CASP8 processing and activation, down-regulating CASP8 proapoptotic function |
| NRG1 | Direct ligand for ERBB3 and ERBB4 tyrosine kinase receptors. Concomitantly recruits ERBB1 and ERBB2 coreceptors, resulting in ligand-stimulated tyrosine phosphorylation and activation of the ERBB receptors. The multiple isoforms perform diverse functions such as inducing growth and differentiation of epithelial, glial, neuronal, and skeletal muscle cells; inducing expression of acetylcholine receptor in synaptic vesicles during the formation of the neuromuscular junction; stimulating lobuloalveolar budding and milk production in the mammary gland and inducing differentiation of mammary tumor cells; stimulating Schwann cell proliferation; implication in the development of the myocardium such as trabeculation of the developing heart. Isoform [10](https://www.uniprot.org/#Isoform_10) may play a role in motor and sensory neuron development. Binds to ERBB4. Binds to ERBB3. Acts as a ligand for integrins and binds (via EGF domain) to integrins ITGAV:ITGB3 or ITGA6:ITGB4. Its binding to integrins and subsequent ternary complex formation with integrins and ERRB3 are essential for NRG1-ERBB signaling. Induces the phosphorylation and activation of MAPK3/ERK1, MAPK1/ERK2 and AKT1. Ligand-dependent ERBB4 endocytosis is essential for the NRG1-mediated activation of these kinases in neurons |

**Supplemental data**

**Figure S1**


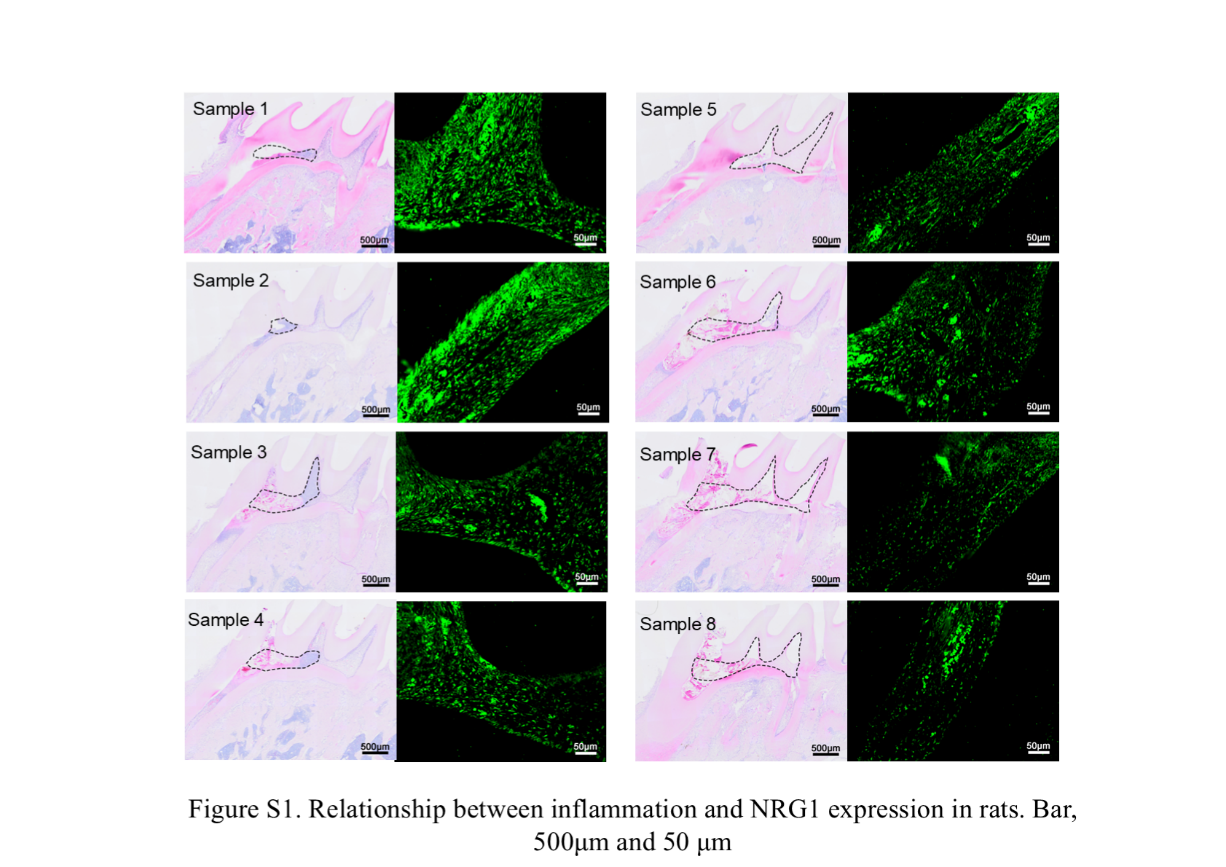


Figure S1. Relationship between inflammation and NRG1 expression in rats. Bar, 500μm and 50 μm.

**Figure S2**


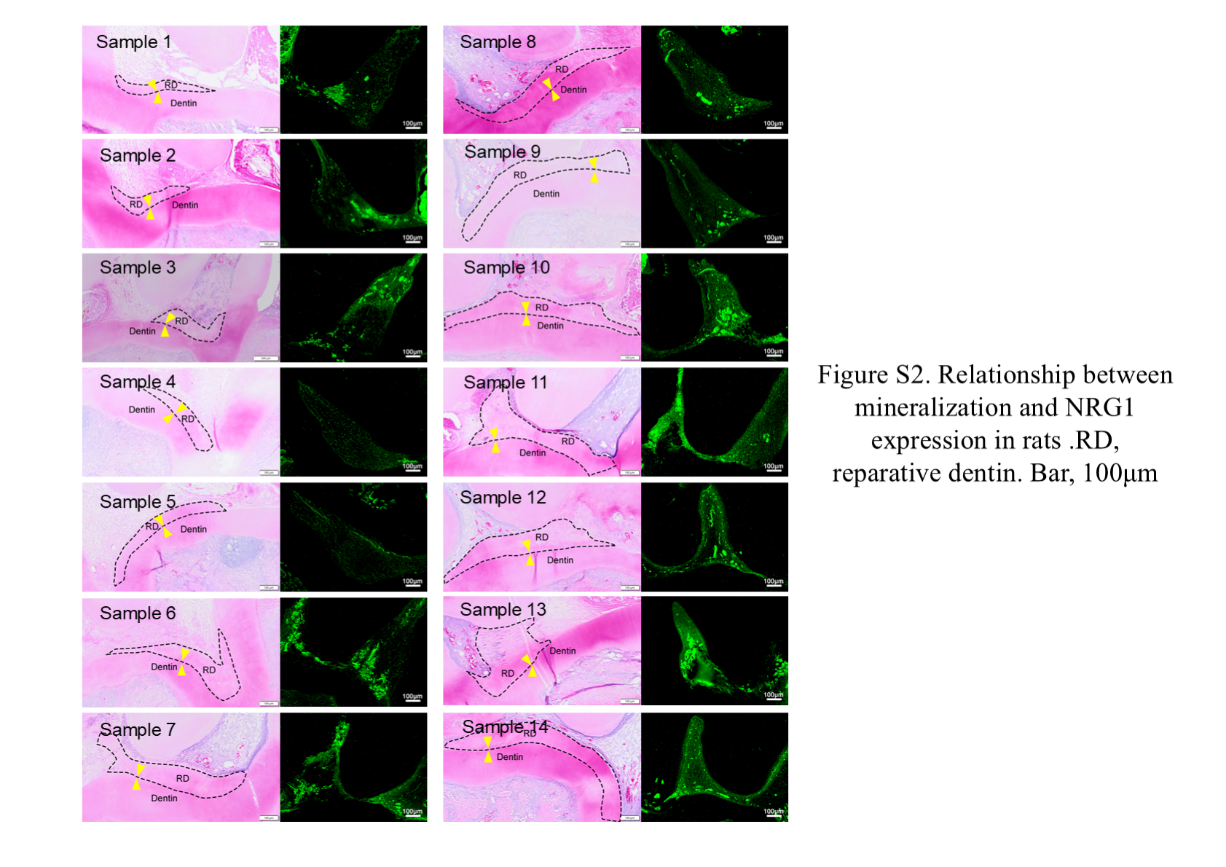


Figure S2. Relationship between mineralization and NRG1 expression in rats. RD, reparative dentin. Bar, 100μm.

**Figure S3**


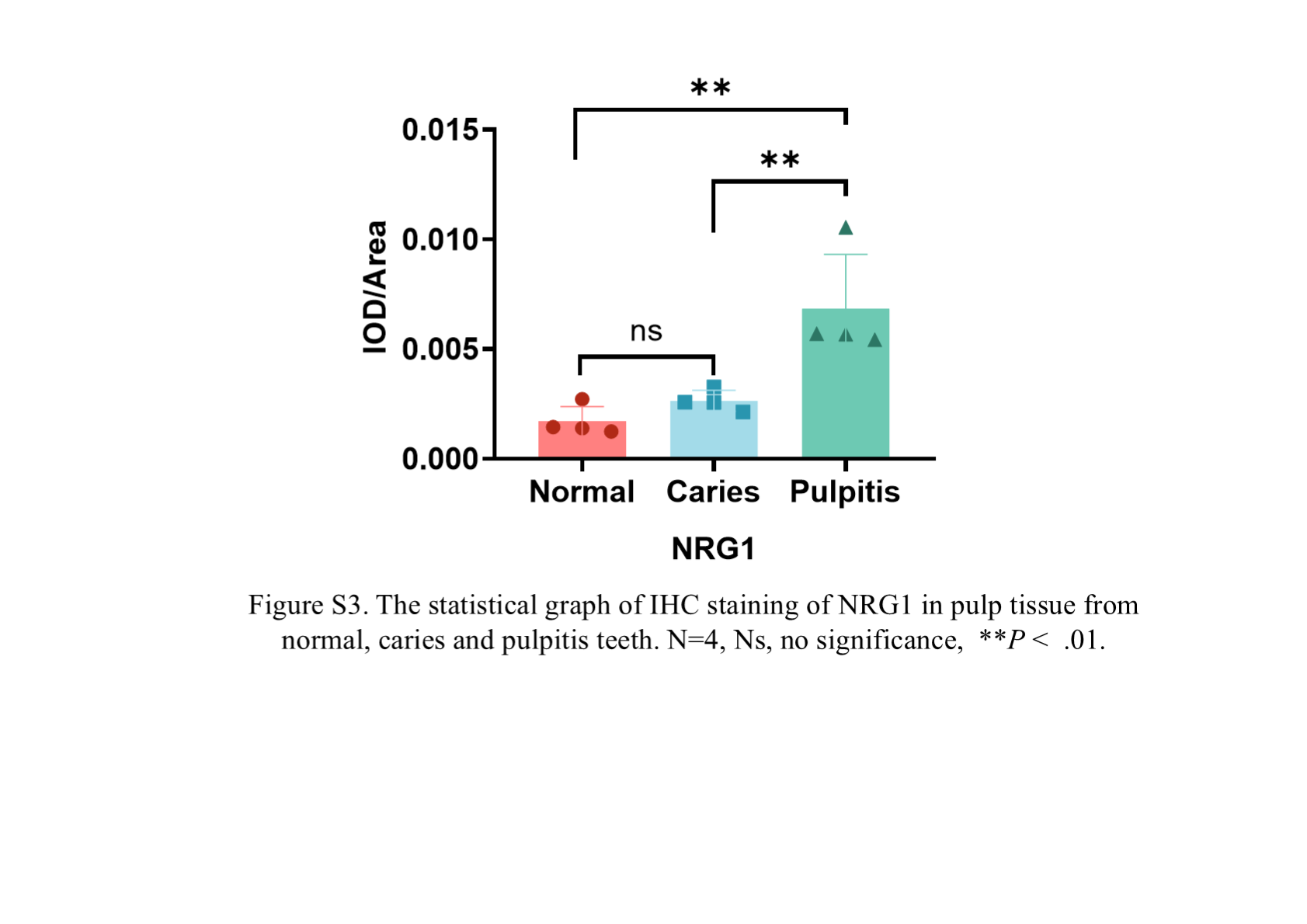


Figure S3. The statistical graph of IHC staining of NRG1 in pulp tissue from normal, caries and pulpitis teeth. N=4, Ns, no significance, ***P* < .01.

**Figure S4**


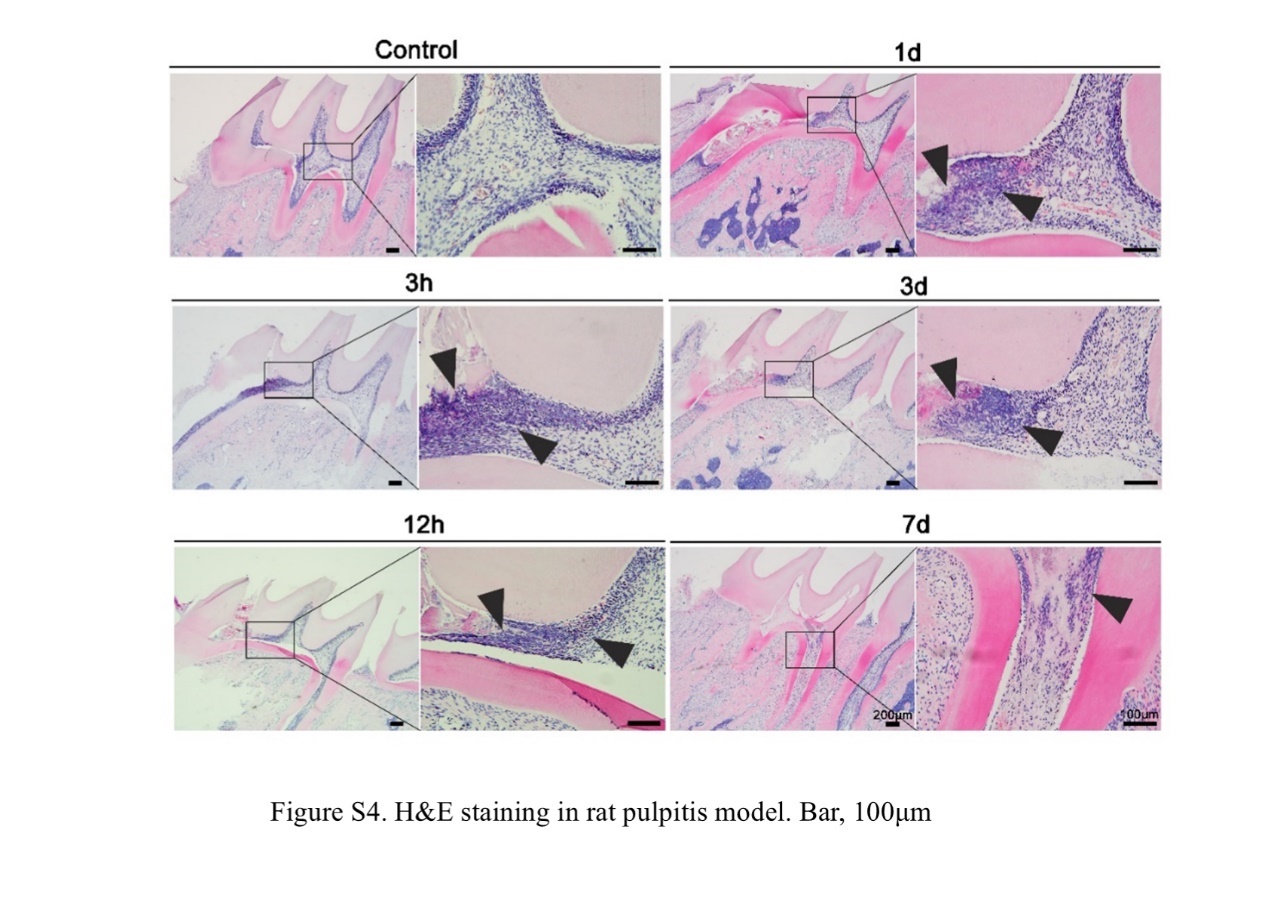


Figure S4. H&E staining in rat pulpitis model. Bar, 100μm.

**Figure S5**


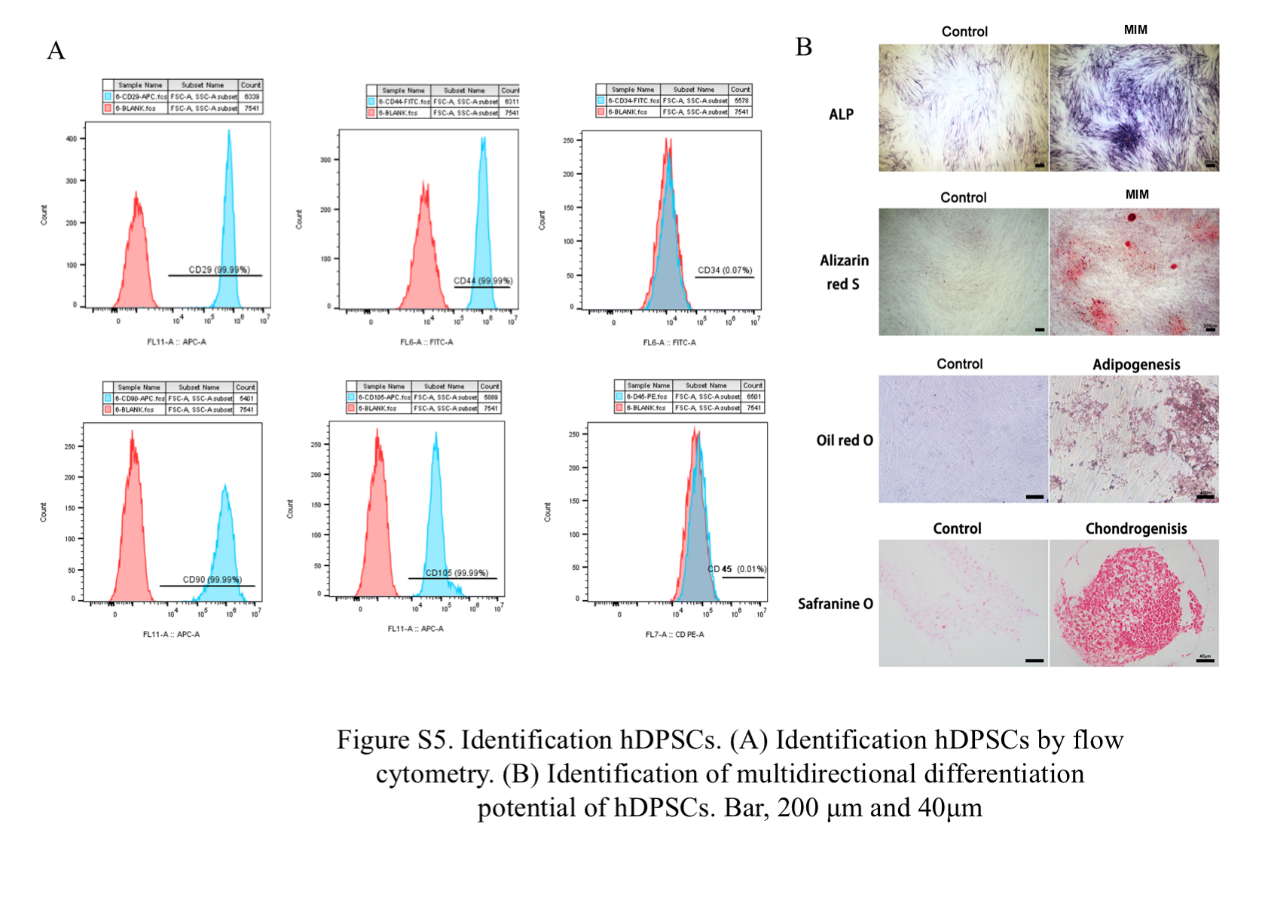


Figure S5. Identification hDPSCs. (A) Identification hDPSCs by flow cytometry. (B) Identification of multidirectional differentiation potential of hDPSCs. Bar, 200 μm and 40μm

**Figure S6**


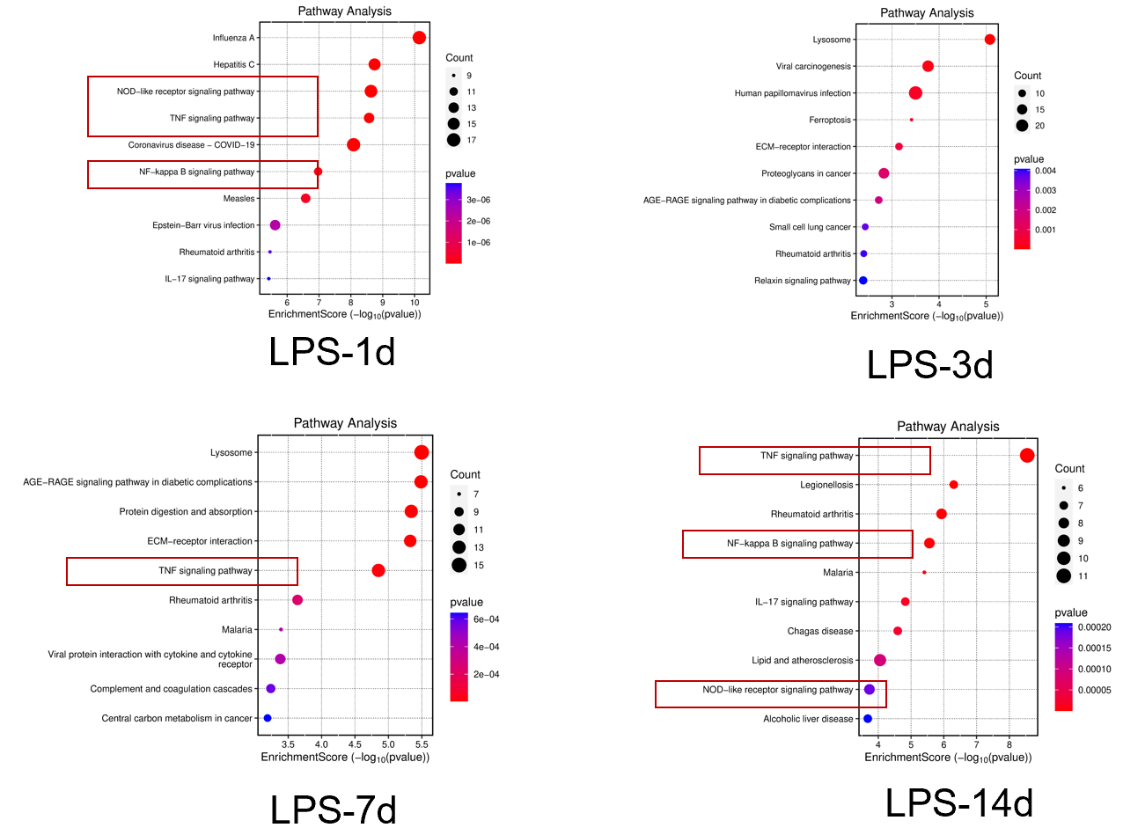


Figure S6. GO pathways analysis of inflammation model of hDPSCs *in vitro*. The graphs were acquired by Bioinformatics platform (<https://www>.bioinformatics.com.cn).

**Figure S7**


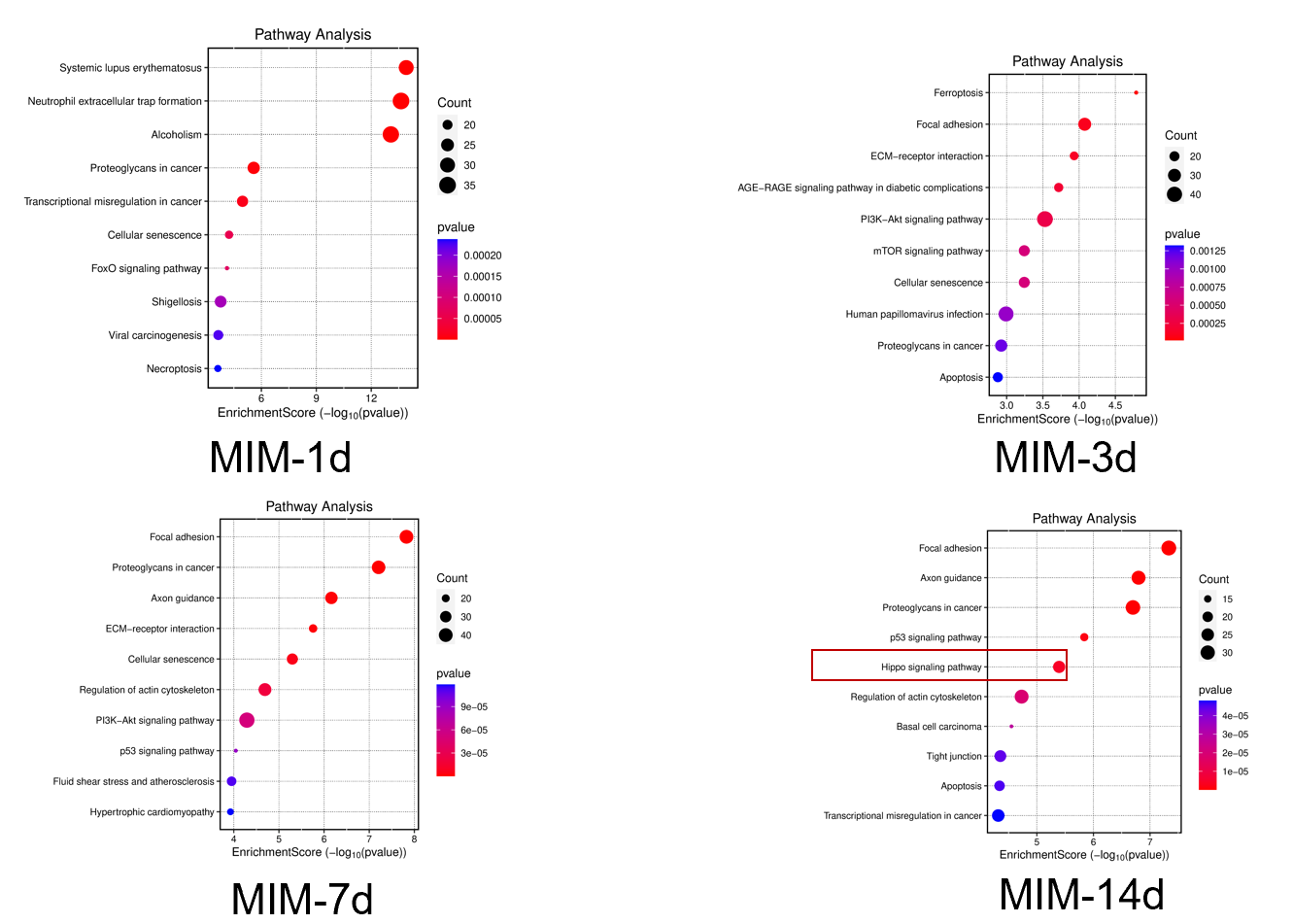


Figure S7. GO pathways analysis of mineralization model of hDPSCs *in vitro*. The graphs were acquired by Bioinformatics platform (https://www.bioinformatics.com.cn).

**Figure S8**


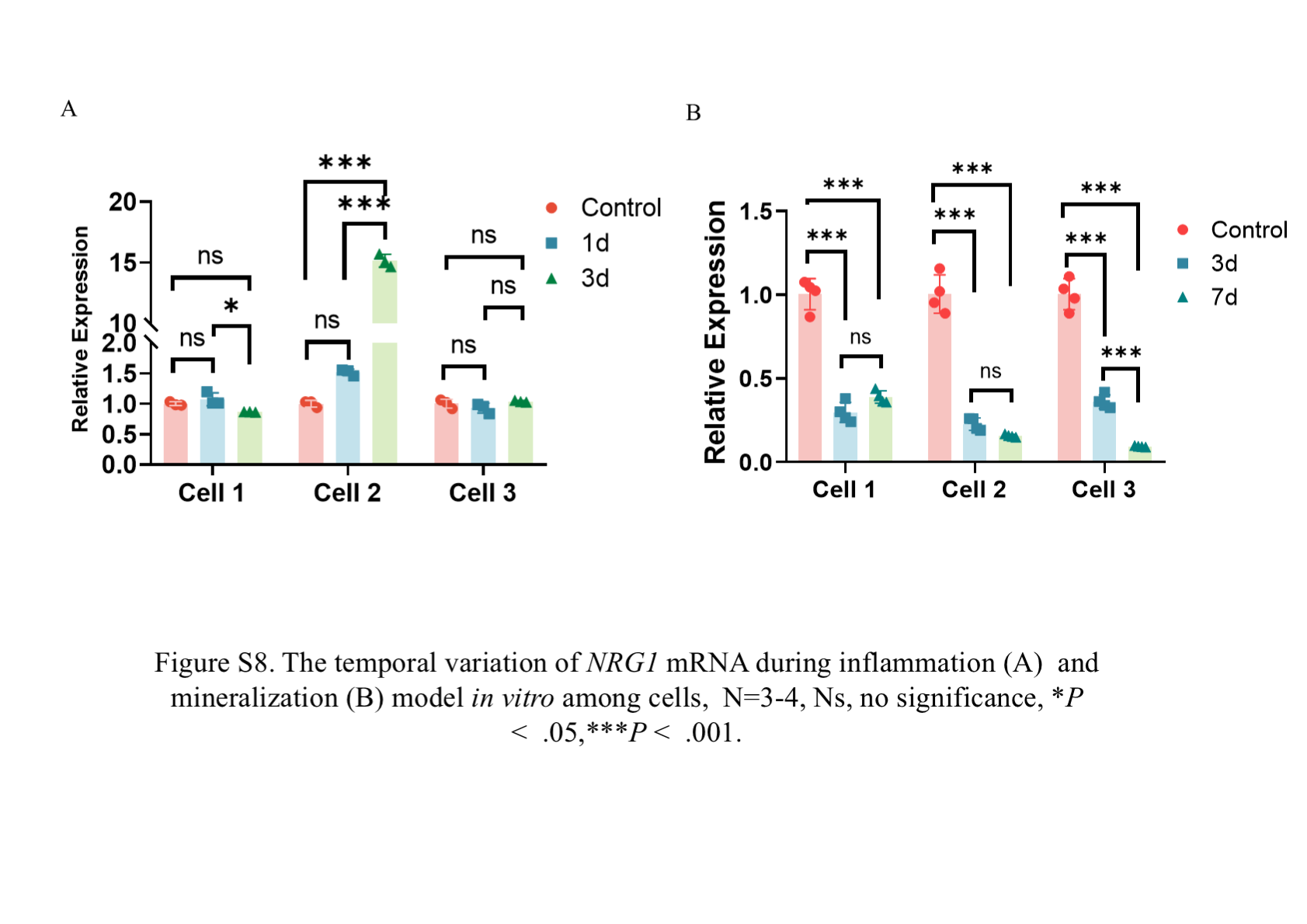


Figure S8. The temporal variation of *NRG1* mRNA during inflammation (A) and mineralization (B) model *in vitro* among cells. N=3-4, Ns, no significance, **P* < .05, ****P* < .001.

**Figure S9**


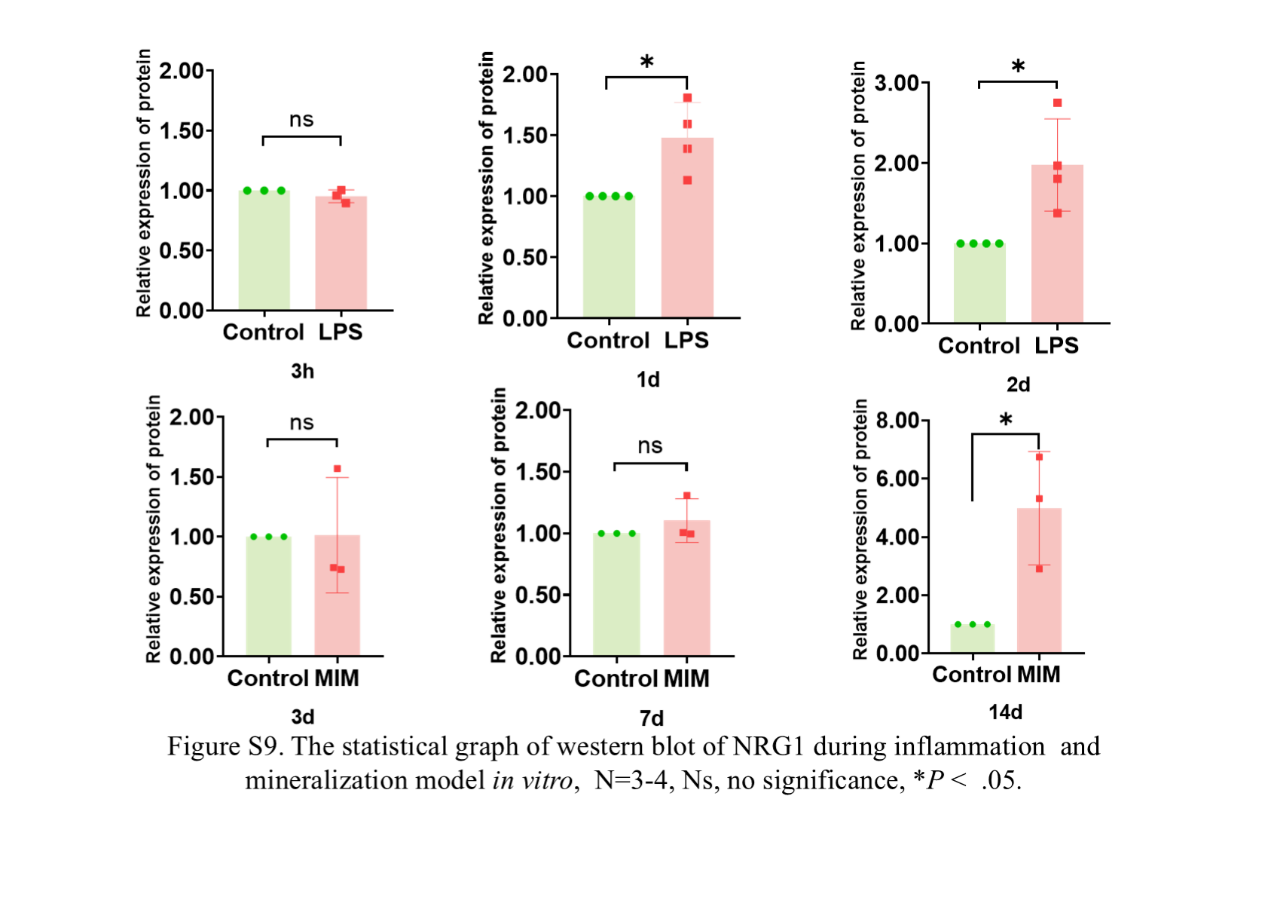


Figure S9. The statistical graph of western blot of NRG1 during inflammation and mineralization model *in vitro*, N=3-4, Ns, no significance, **P* < .05.

**Figure S10**


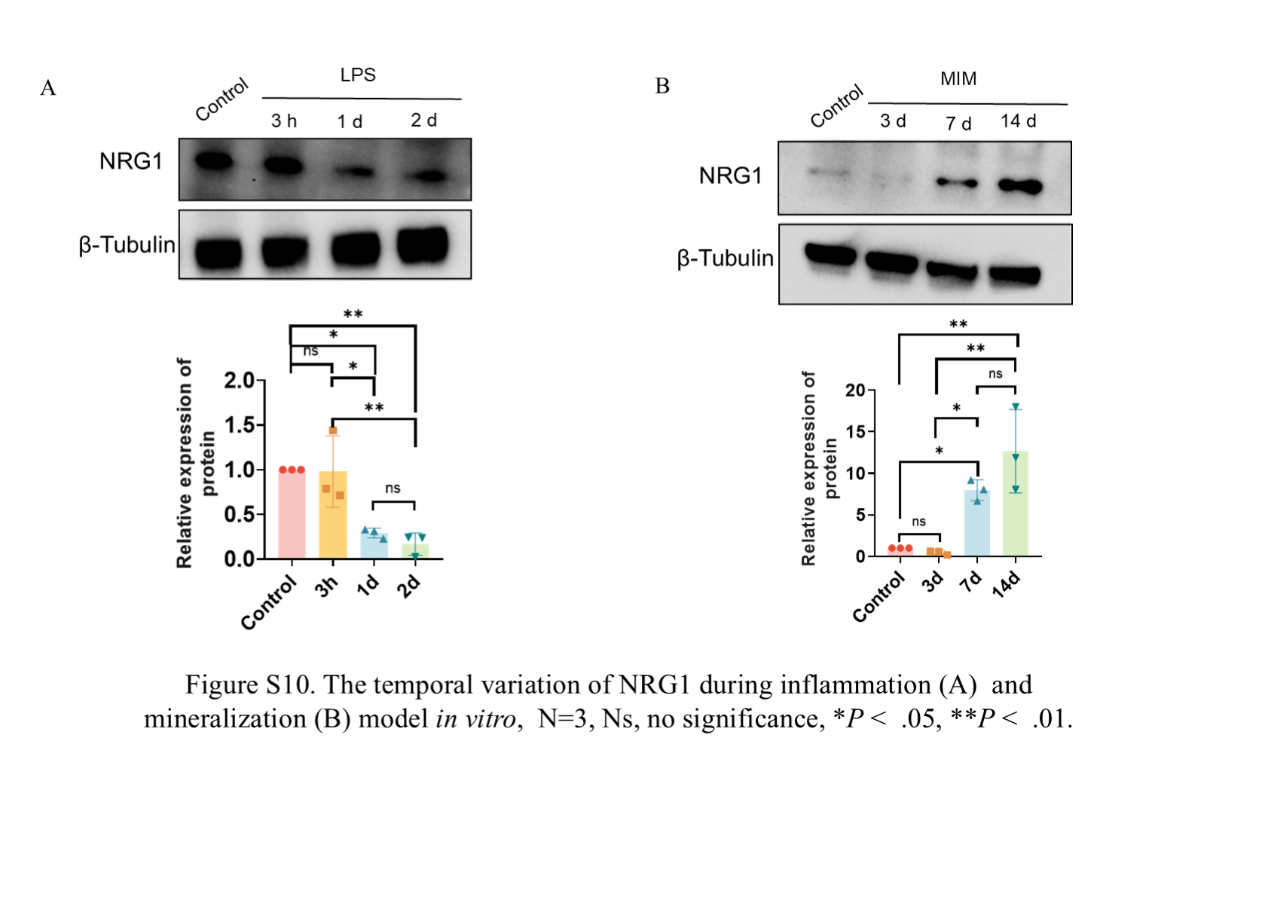


Figure S10. The temporal variation of NRG1 during inflammation (A) and mineralization (B) model *in vitro*, N=3, Ns, no significance, **P* < .05, ***P* < .01.

**Figure S11**


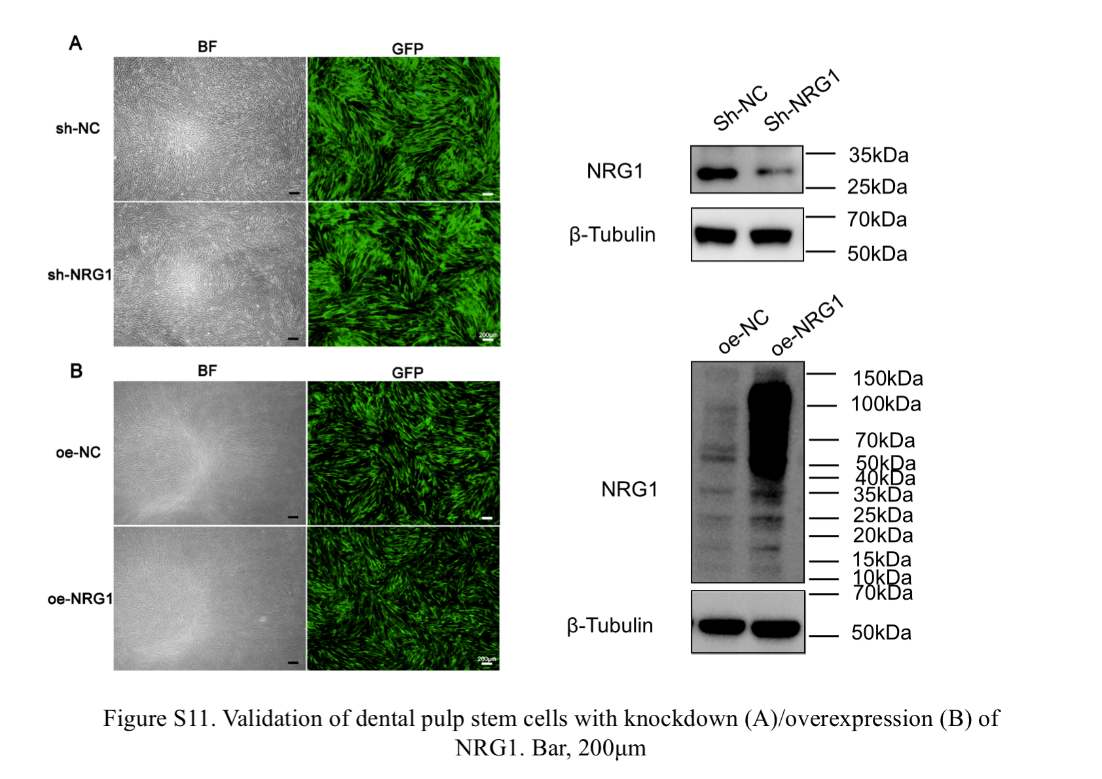


Figure S11. Validation of dental pulp stem cells with knockdown (A)/overexpression (B) of NRG1. Bar, 200μm.

**Figure S12**


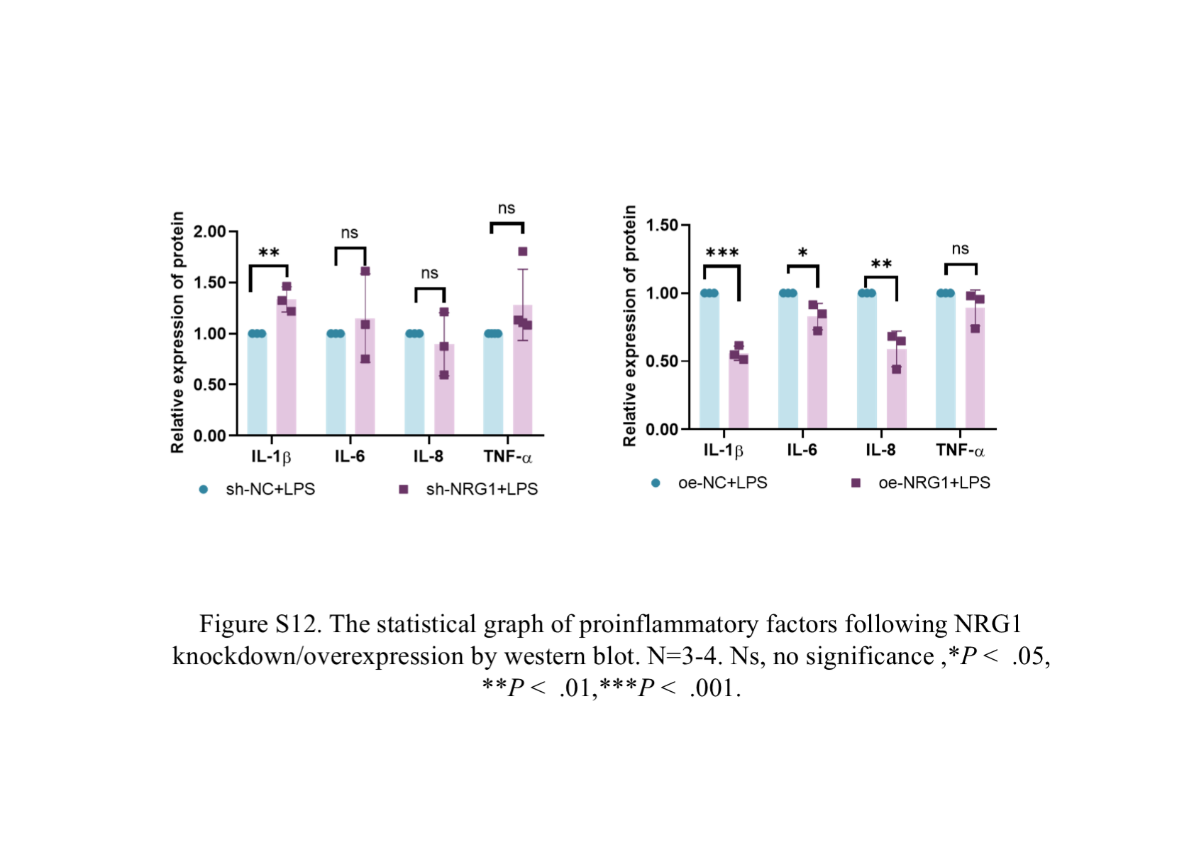


Figure S12. The statistical graph of proinflammatory factors following NRG1 knockdown/overexpression by western blot. N=3-4. Ns, no significance ,**P* < .05, ***P* < .01,****P* < .001.

**Figure S13**


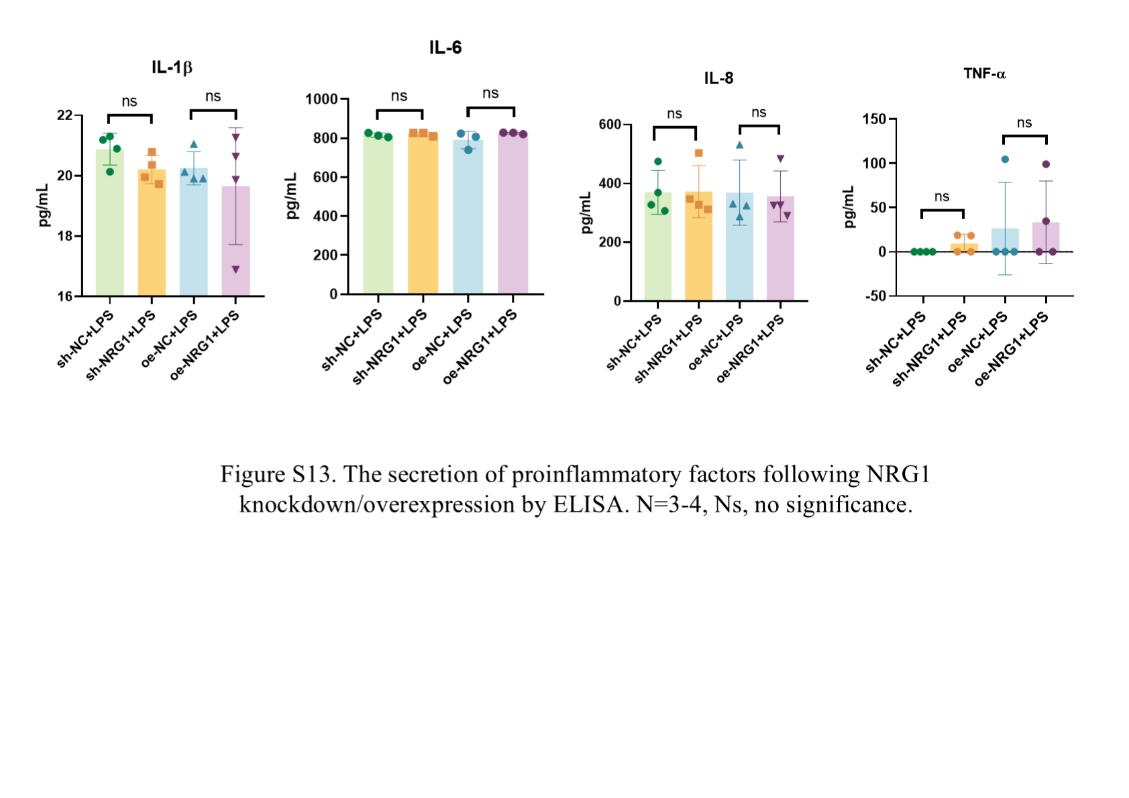


Figure S13. The secretion of proinflammatory factors following NRG1 knockdown/overexpression by ELISA. N=3-4, Ns, no significance.

**Figure S14**


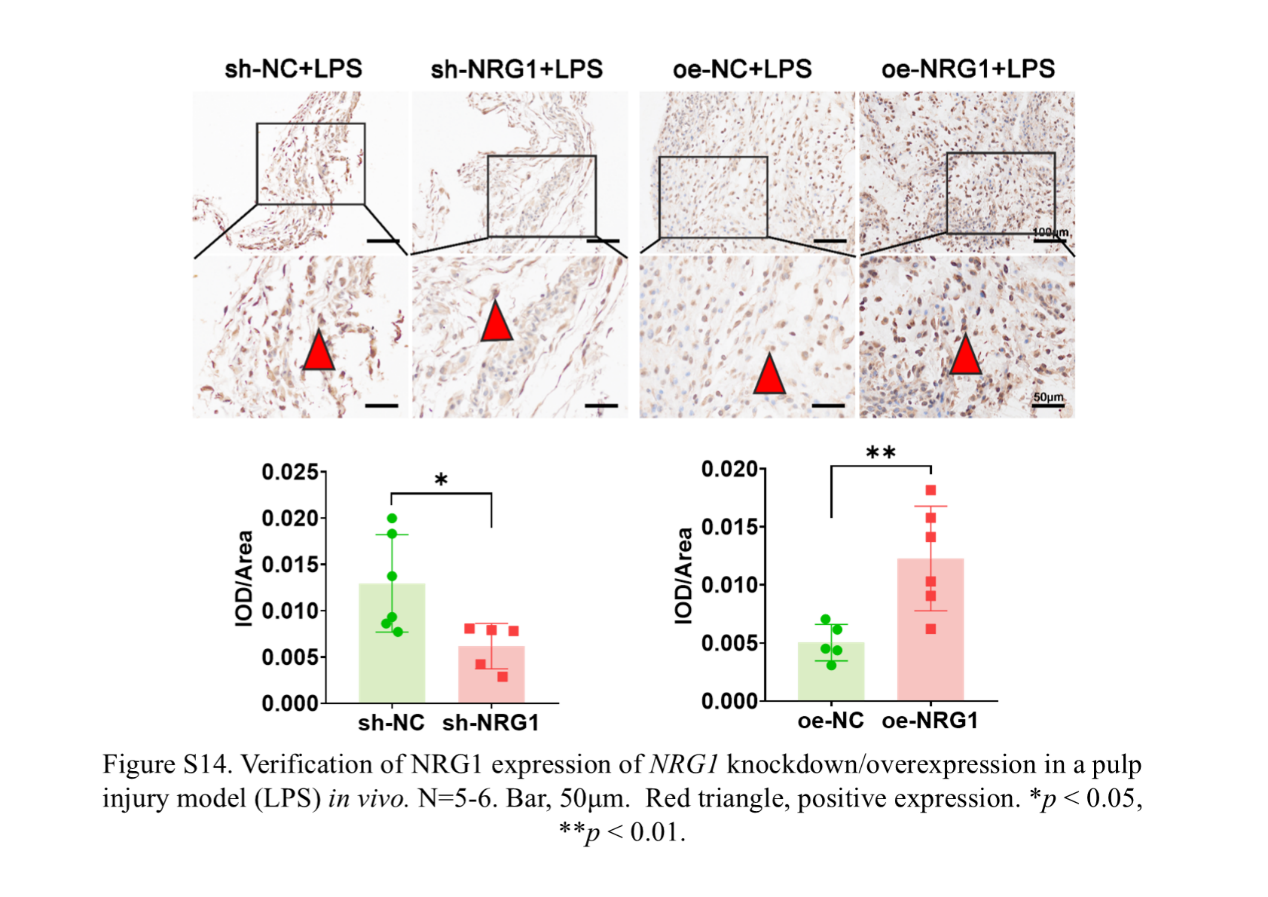


Figure S14. Verification of NRG1 expression of *NRG1* knockdown/overexpression in a pulp injury model (LPS) *in vivo.* N=5-6. Bar, 50μm. Red triangle, positive expression. **p* < 0.05, ***p* < 0.01.

**Figure S15**


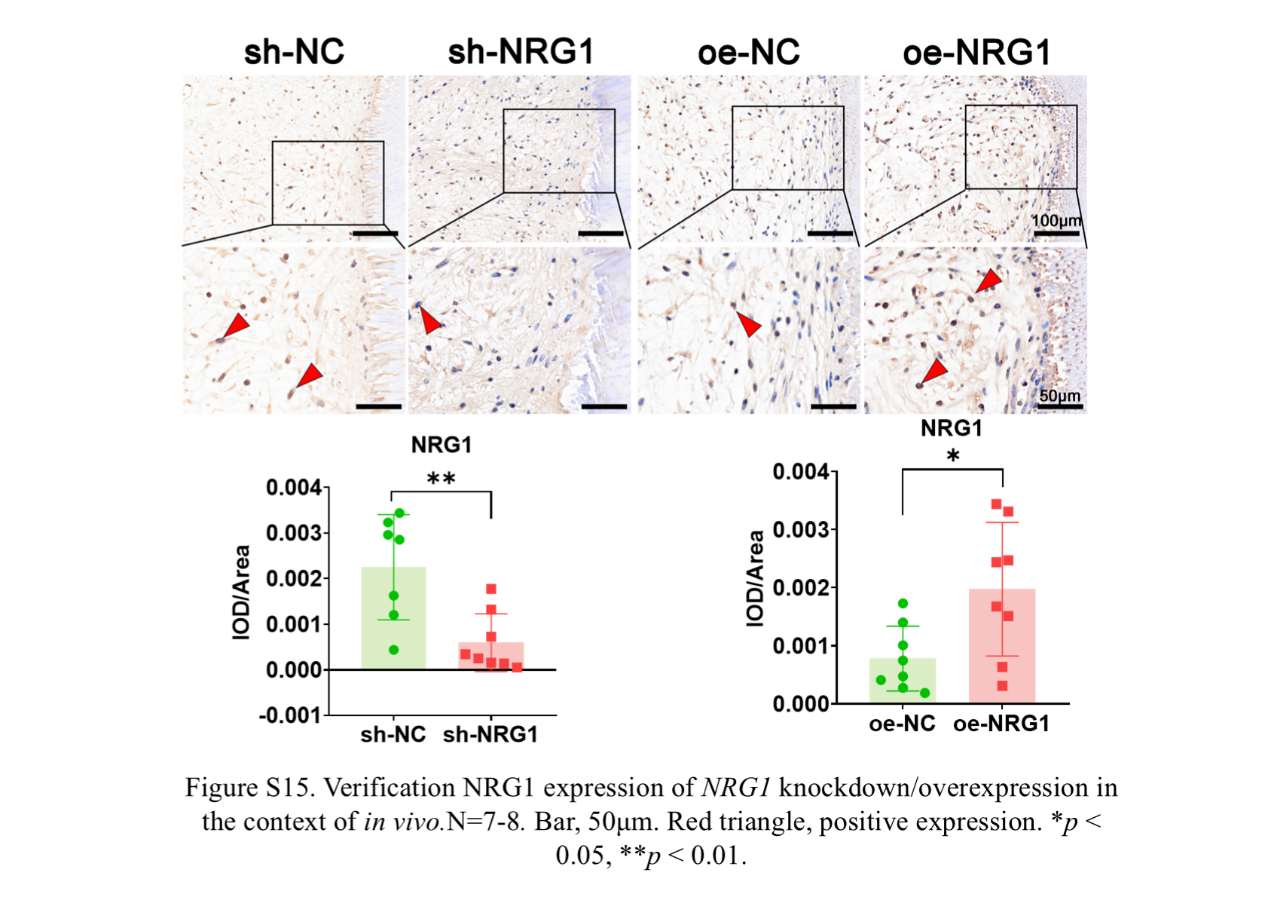


Figure S15. Verification NRG1 expression of *NRG1* knockdown/overexpression in the context of *in vivo.* N=7-8*.* Bar, 50μm. Red triangle, positive expression. **p* < 0.05, ***p* < 0.01.

**Figure S16**


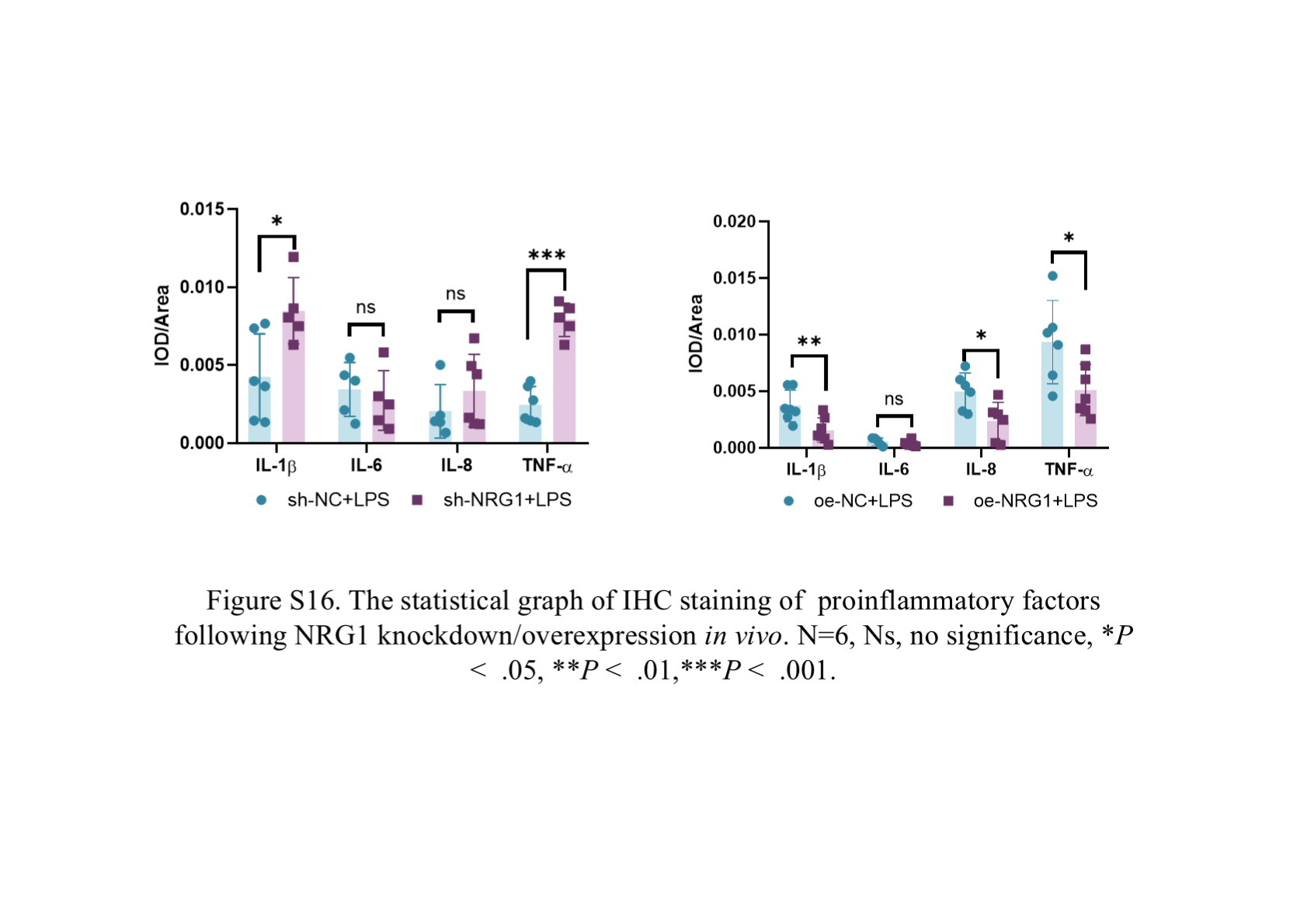


Figure S16. The statistical graph of IHC staining of proinflammatory factors following NRG1 knockdown/overexpression *in vivo*. N=6, Ns, no significance, **P* < .05, ***P* < .01, ****P* < .001.

**Figure S17**


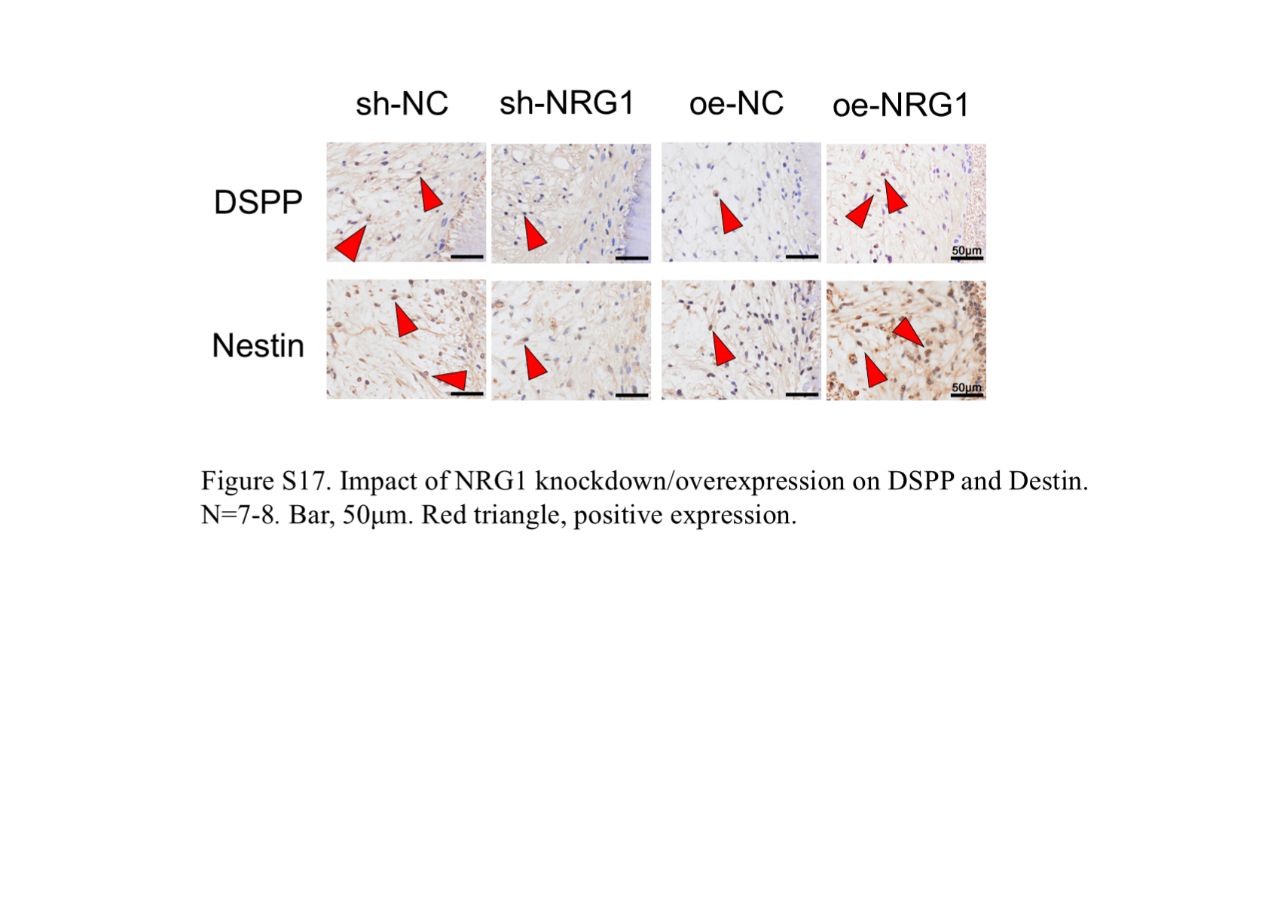


Figure S17. Impact of NRG1 knockdown/overexpression on DSPP and Destin. N=7-8*.* Bar, 50μm. Red triangle, positive expression.

**Figure S18**


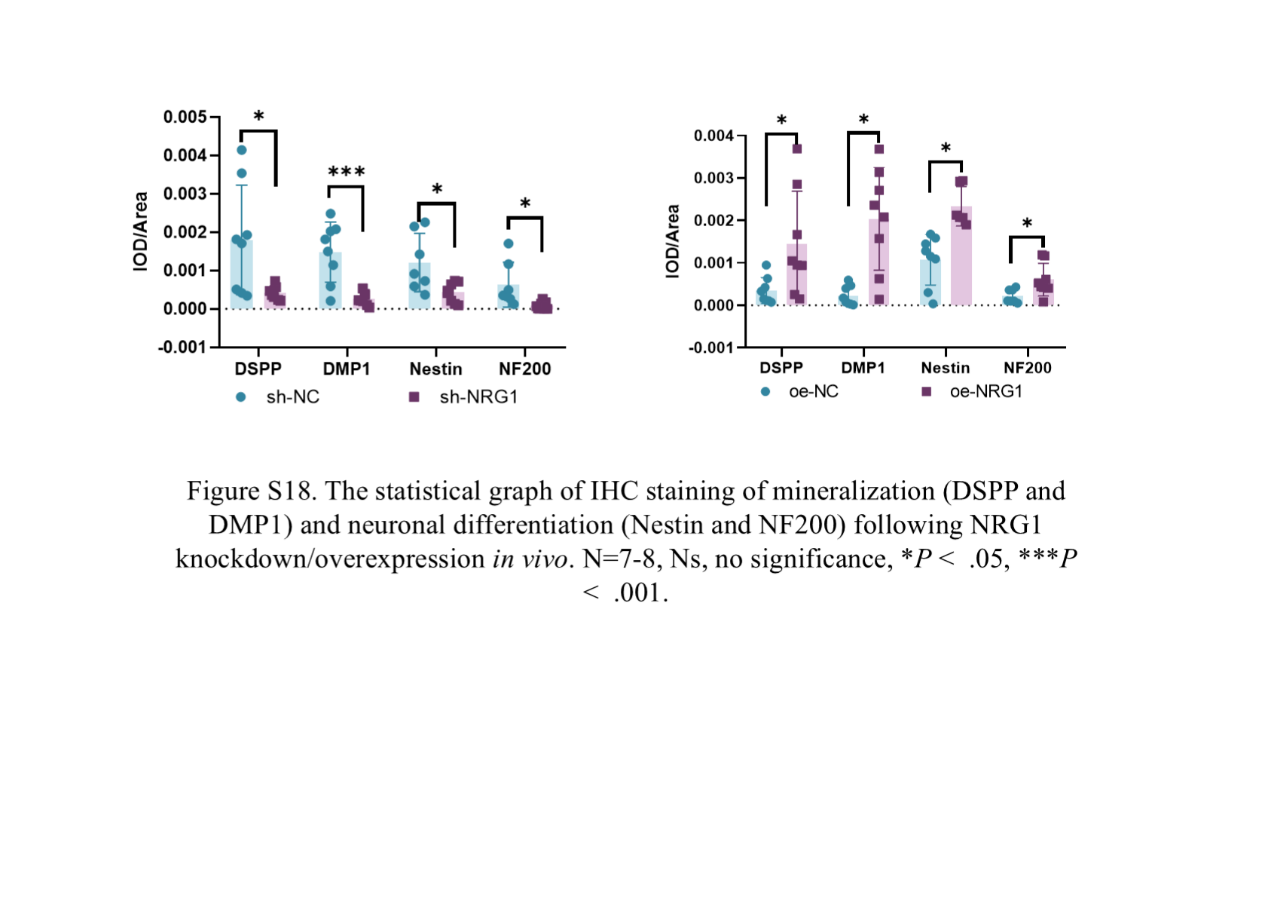


Figure S18. The statistical graph of IHC staining of mineralization (DSPP and DMP1) and neuronal differentiation (Nestin and NF200) following NRG1 knockdown/overexpression *in vivo*. N=7-8, Ns, no significance, **P* < .05, ****P* < .001.

**Figure S19**


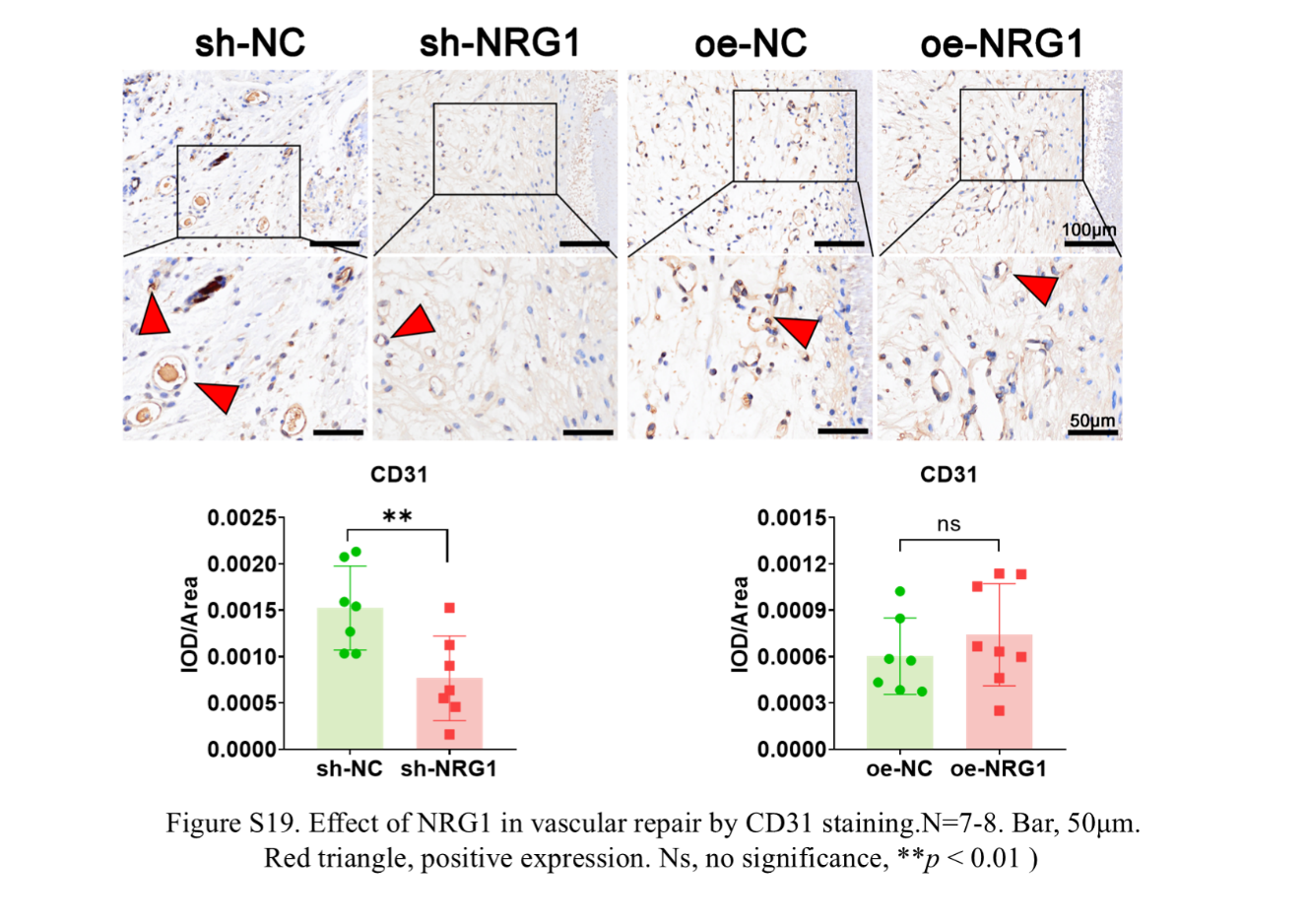


Figure S19. Effect of NRG1 in vascular repair by CD31 staining. N=7-8. Bar, 50μm. Red triangle, positive expression. Ns, no significance, ***p* < 0.01.

**Figure S20**


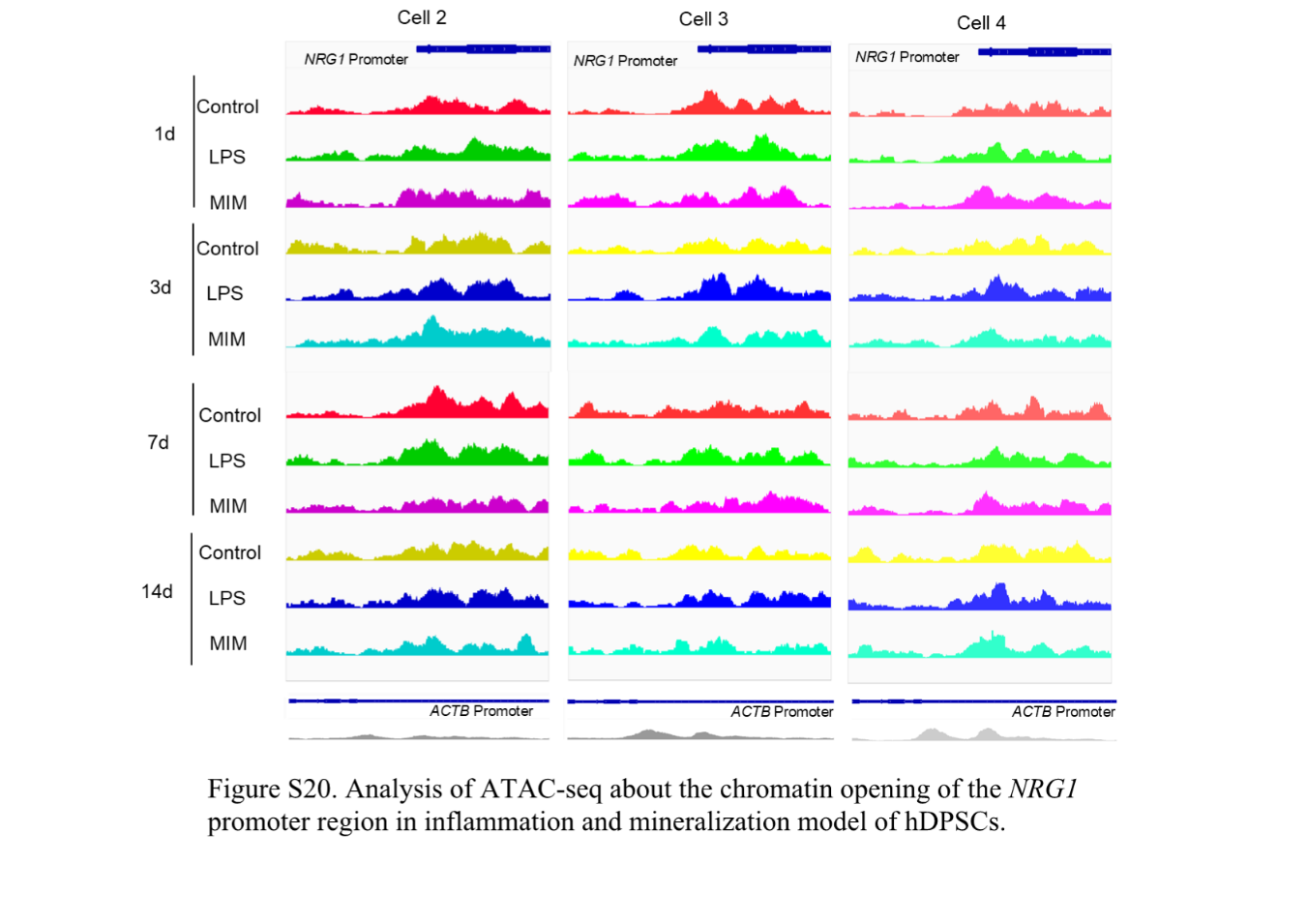


Figure S20. Analysis of ATAC-seq about the chromatin opening of the *NRG1* promoter region in inflammation and mineralization model of hDPSCs.

**Figure S21**


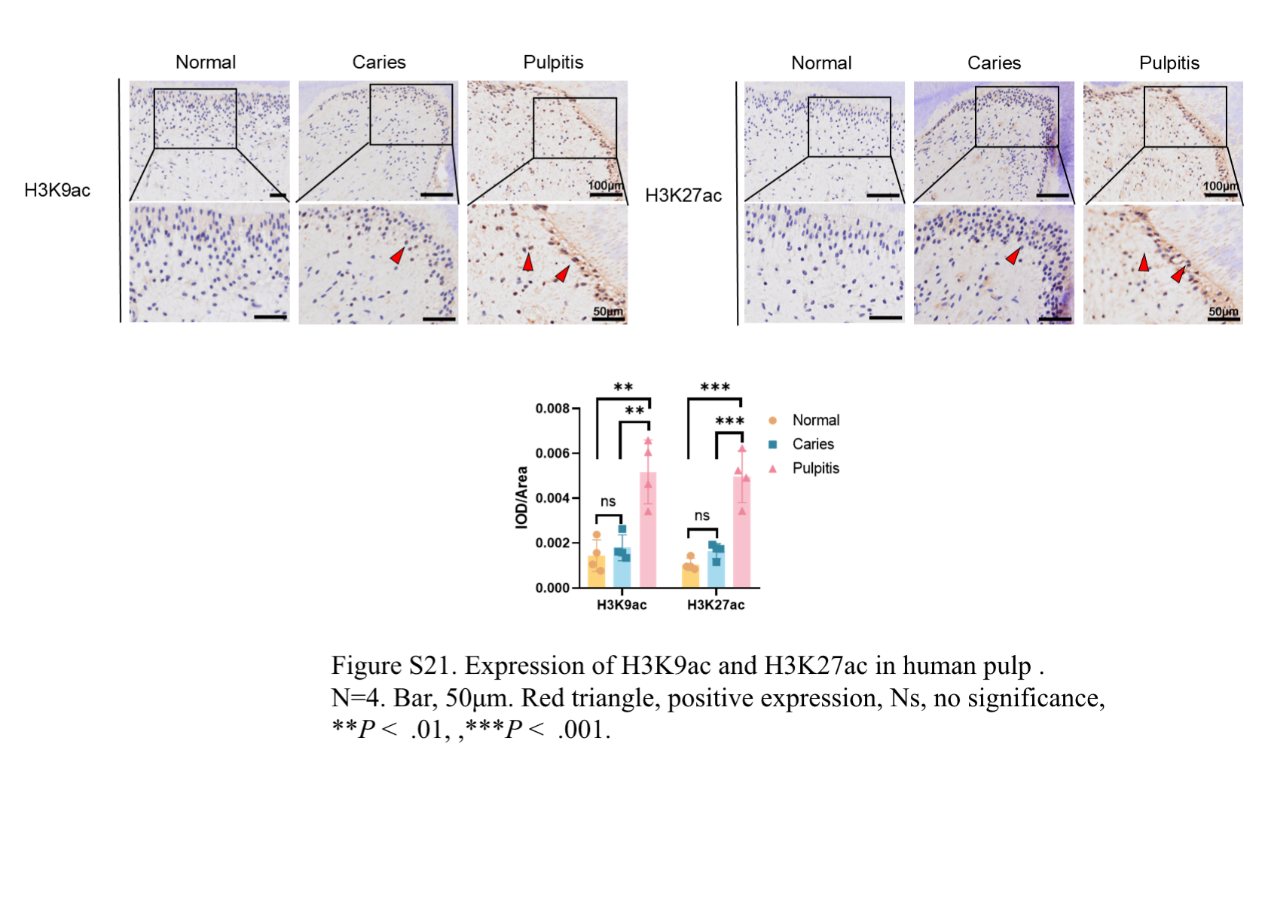


Figure S21. Expression of H3K9ac and H3K27ac in human pulp . N=4. Bar, 50μm. Red triangle, positive expression, Ns, no significance, ***P* < .01, ,****P* < .001.

**Figure S22**


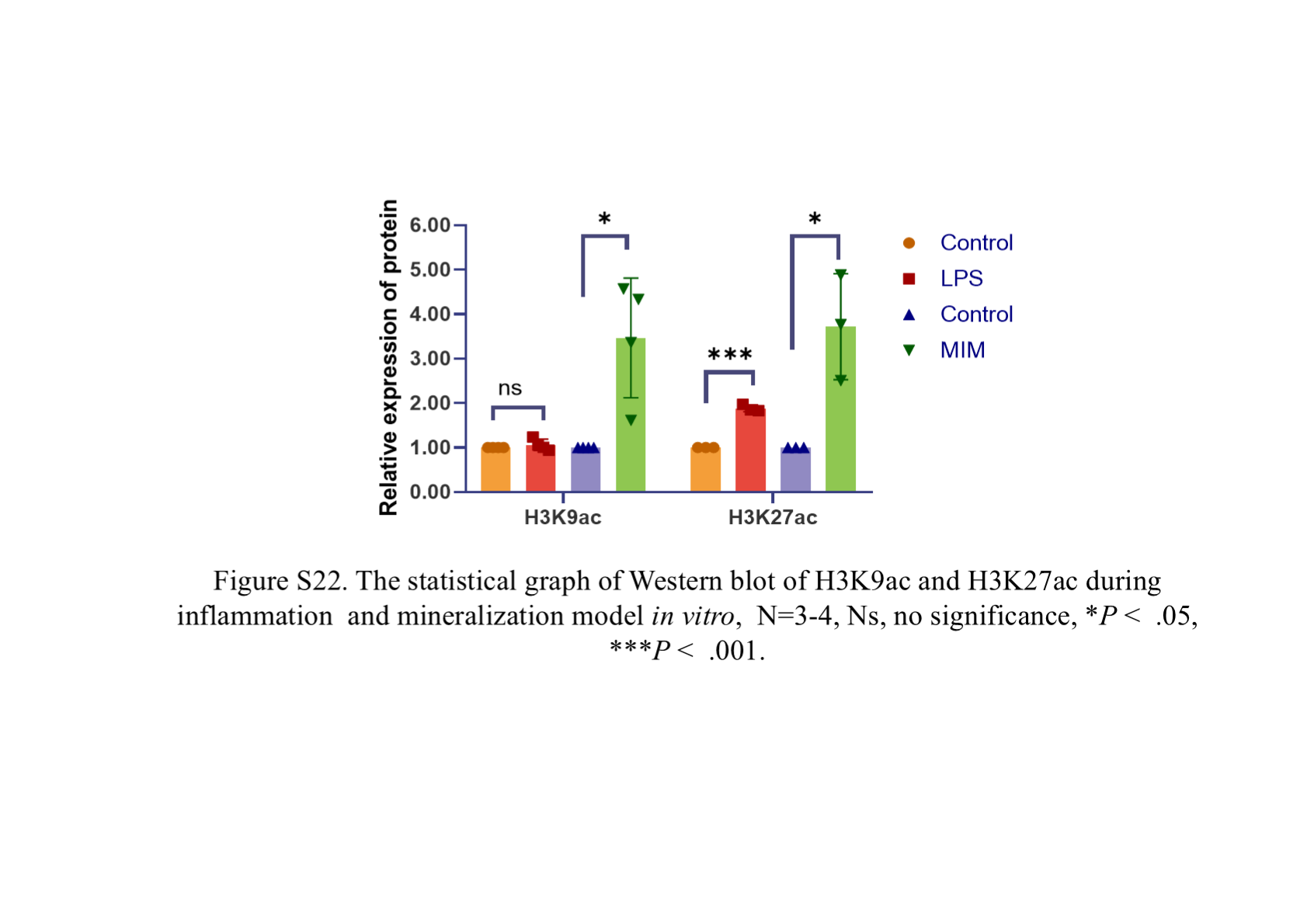


Figure S22. The statistical graph of Western blot of H3K9ac and H3K27ac during inflammation and mineralization model *in vitro*, N=3-4, Ns, no significance, **P* < .05, ****P* < .001.

**Figure S23**


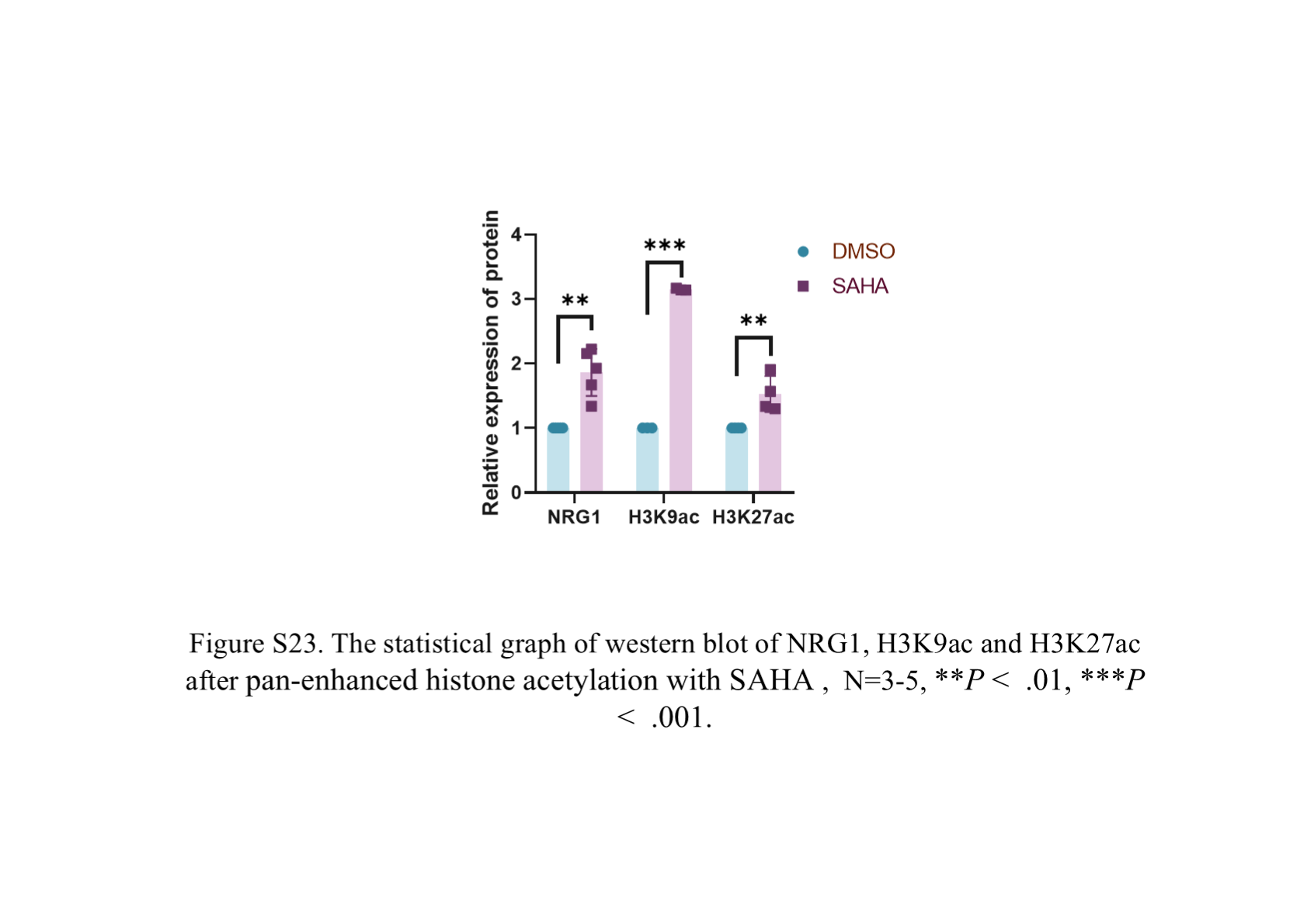


Figure S23. The statistical graph of western blot of NRG1, H3K9ac and H3K27ac after pan-enhanced histone acetylation with SAHA, N=3-5, ***P* < .01, ****P* < .001.

**Figure S24**


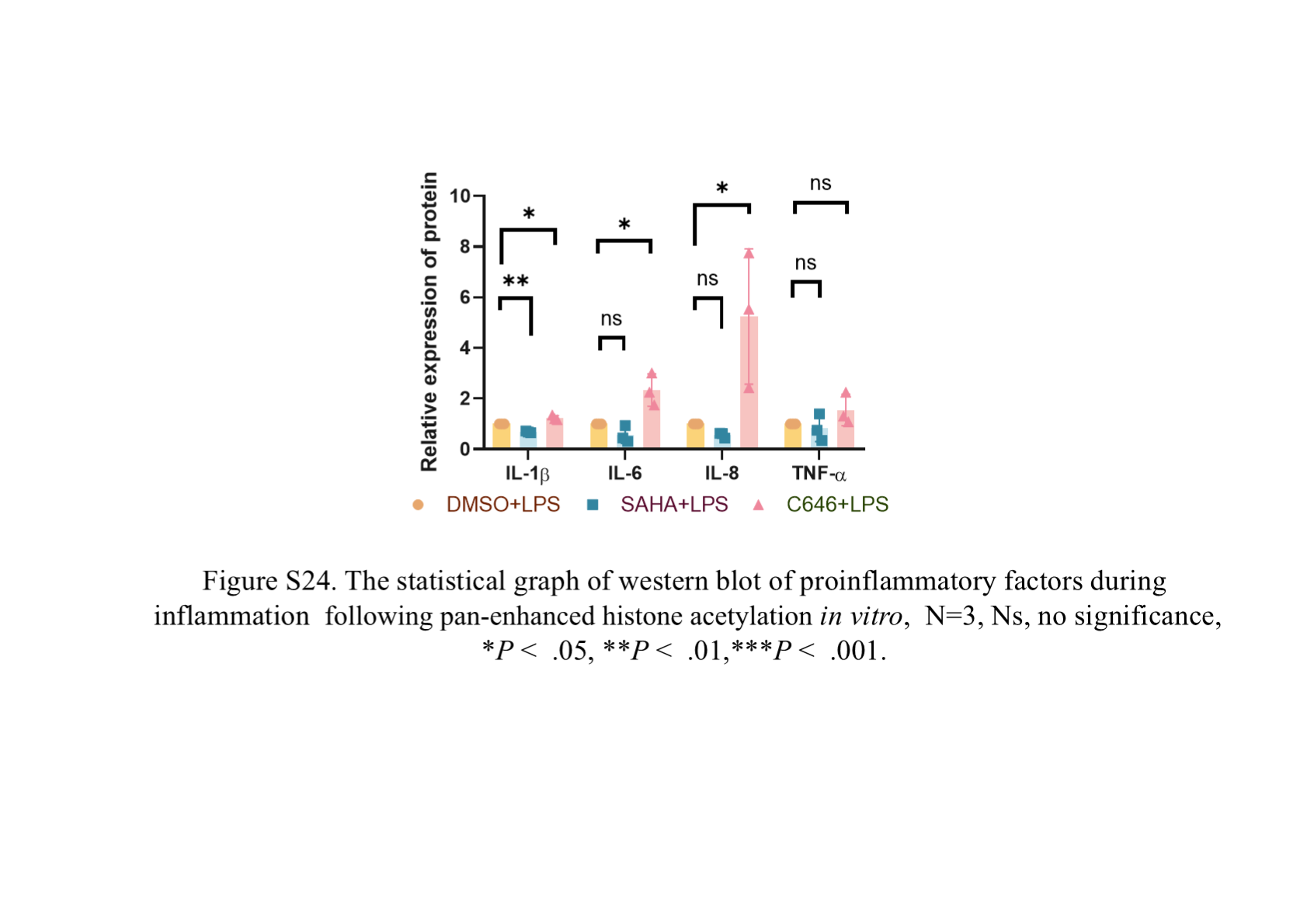


Figure S24. The statistical graph of western blot of proinflammatory factors during inflammation following pan-enhanced histone acetylation *in vitro*, N=3, Ns, no significance, **P* < .05, ***P* < .01, ****P* < .001.

**Figure S25**


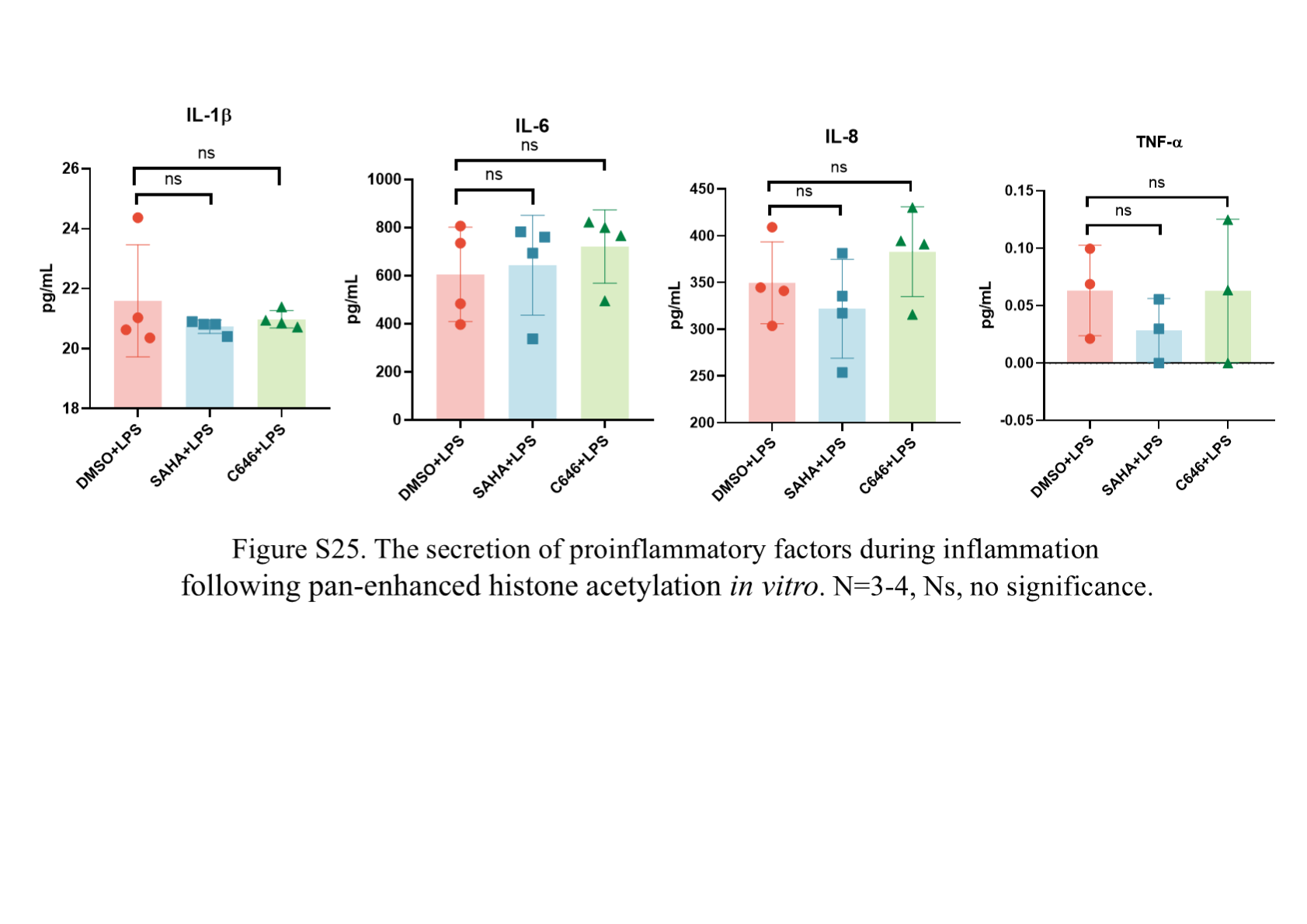


Figure S25. The secretion of proinflammatory factors during inflammation following pan-enhanced histone acetylation *in vitro*. N=3-4, Ns, no significance.

**Figure S26**


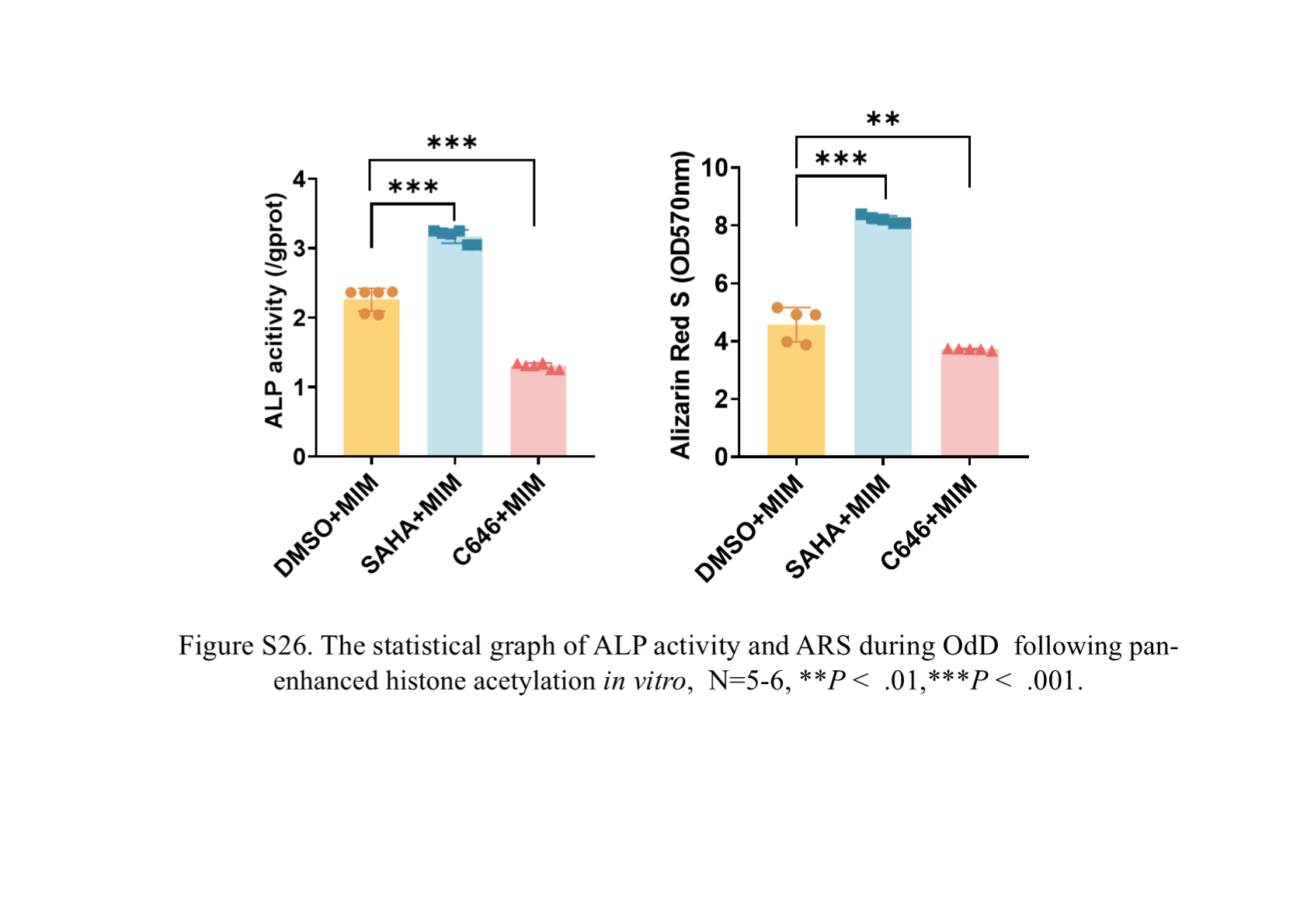


Figure S26. The statistical graph of ALP activity and ARS during OdD following pan-enhanced histone acetylation *in vitro*, N=5-6, ***P* < .01, ****P* < .001.

**Figure S27**


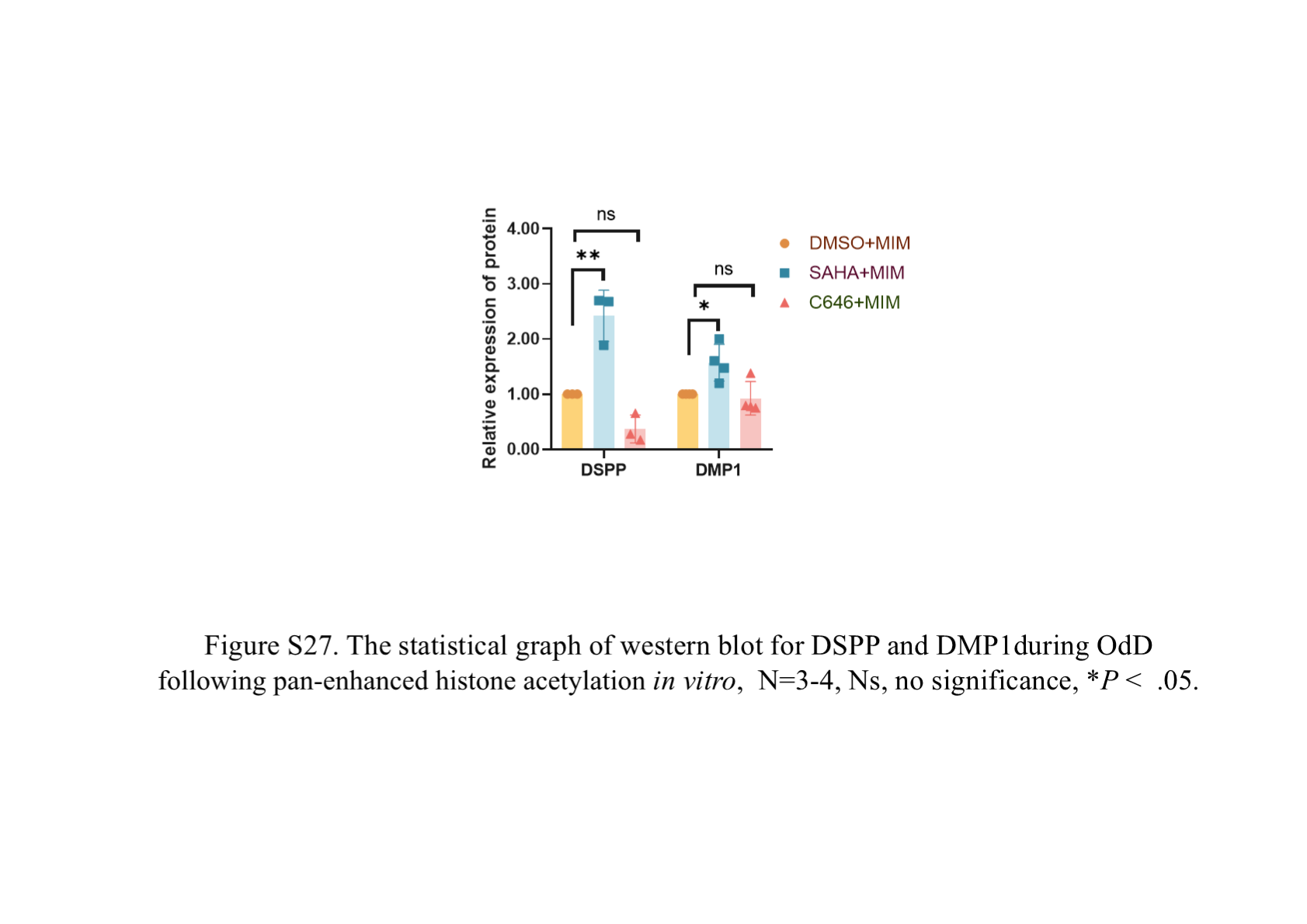


Figure S27. The statistical graph of western blot for DSPP and DMP1during OdD following pan-enhanced histone acetylation *in vitro*, N=3-4, Ns, no significance, **P* < .05.

**Figure S28**


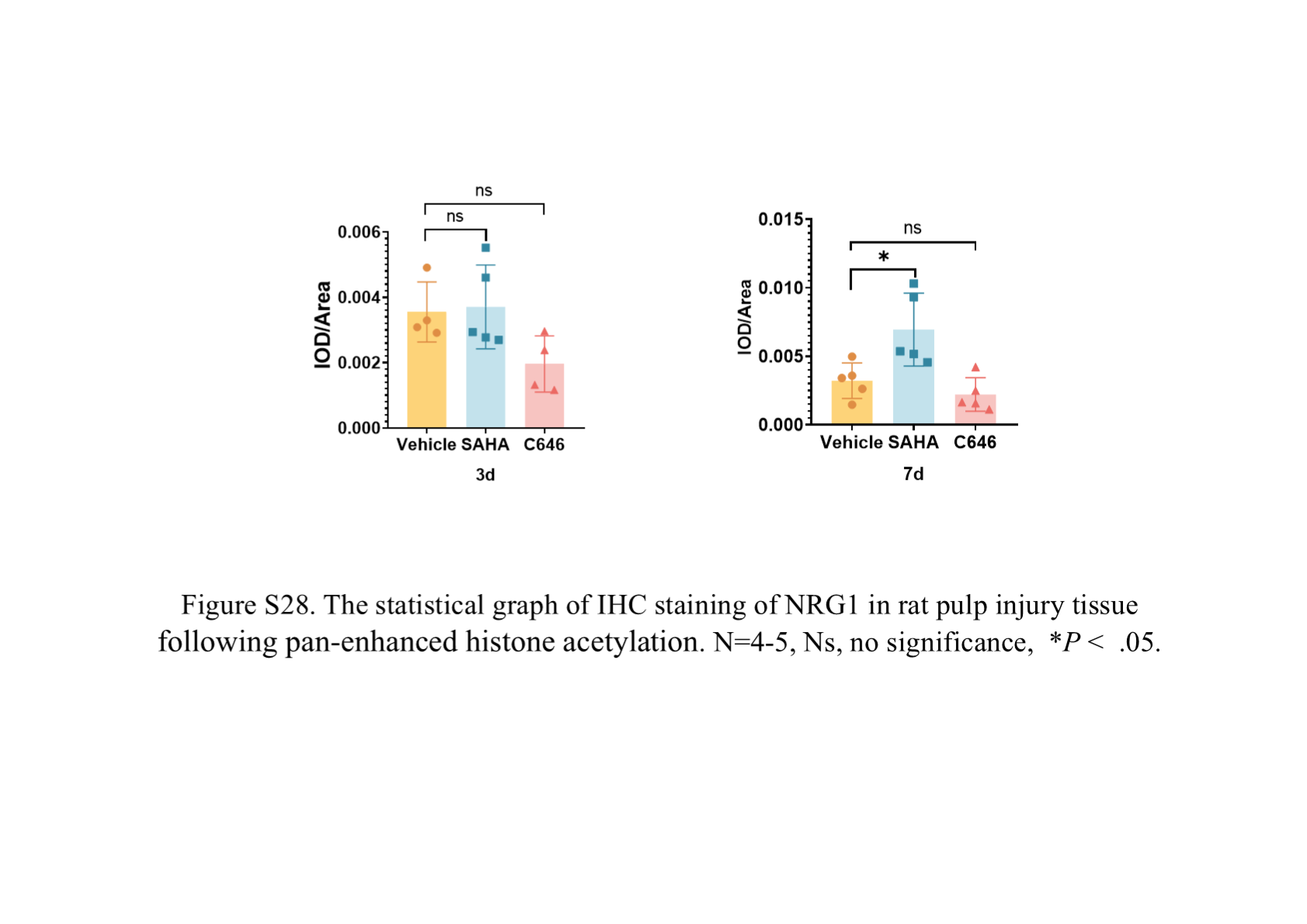


Figure S28. The statistical graph of IHC staining of NRG1 in rat pulp injury tissue following pan-enhanced histone acetylation. N=4-5, Ns, no significance, **P* < .05.

**Figure S29**


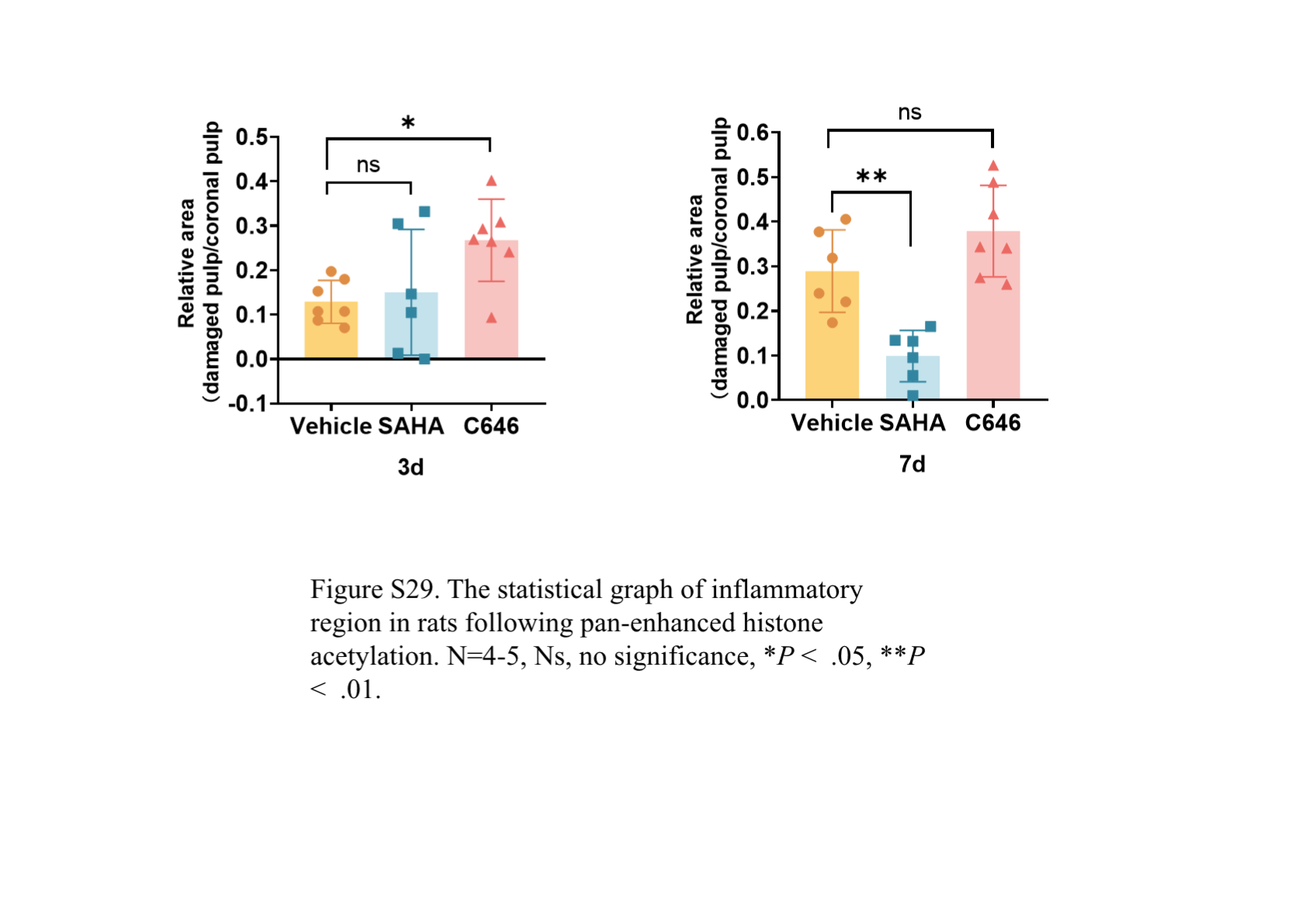


Figure S29. The statistical graph of inflammatory region in rats following pan-enhanced histone acetylation. N=4-5, Ns, no significance, **P* < .05, ***P* < .01.

**Figure S30**


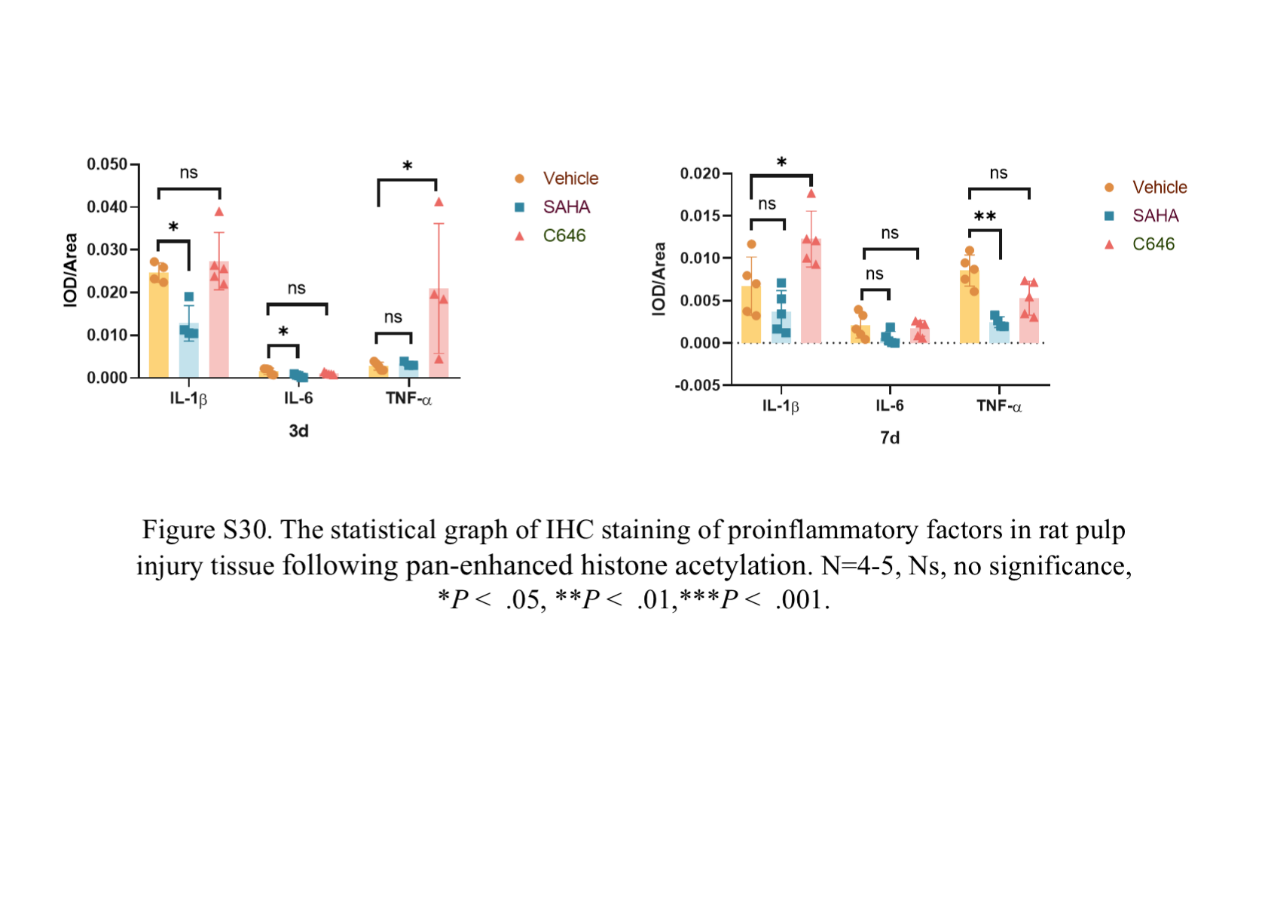


Figure S30. The statistical graph of IHC staining of proinflammatory factors in rat pulp injury tissue following pan-enhanced histone acetylation. N=4-5, Ns, no significance, **P* < .05, ***P* < .01,****P* < .001.

**Figure S31**


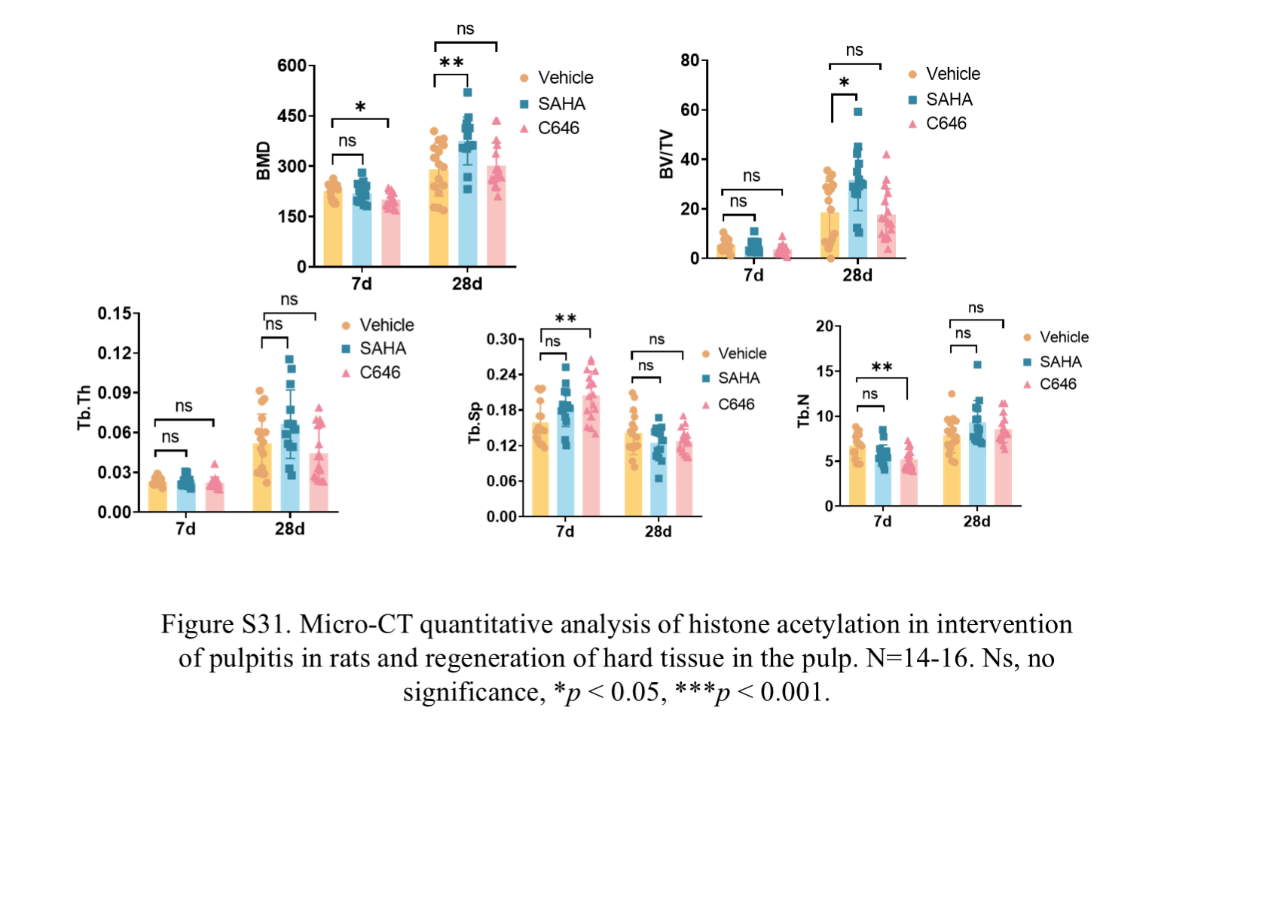


Figure S31. Micro-CT quantitative analysis of histone acetylation in intervention of pulpitis in rats and regeneration of hard tissue in the pulp. N=14-16. Ns, no significance, **p* < 0.05, ****p* < 0.001.

**Figure S32**


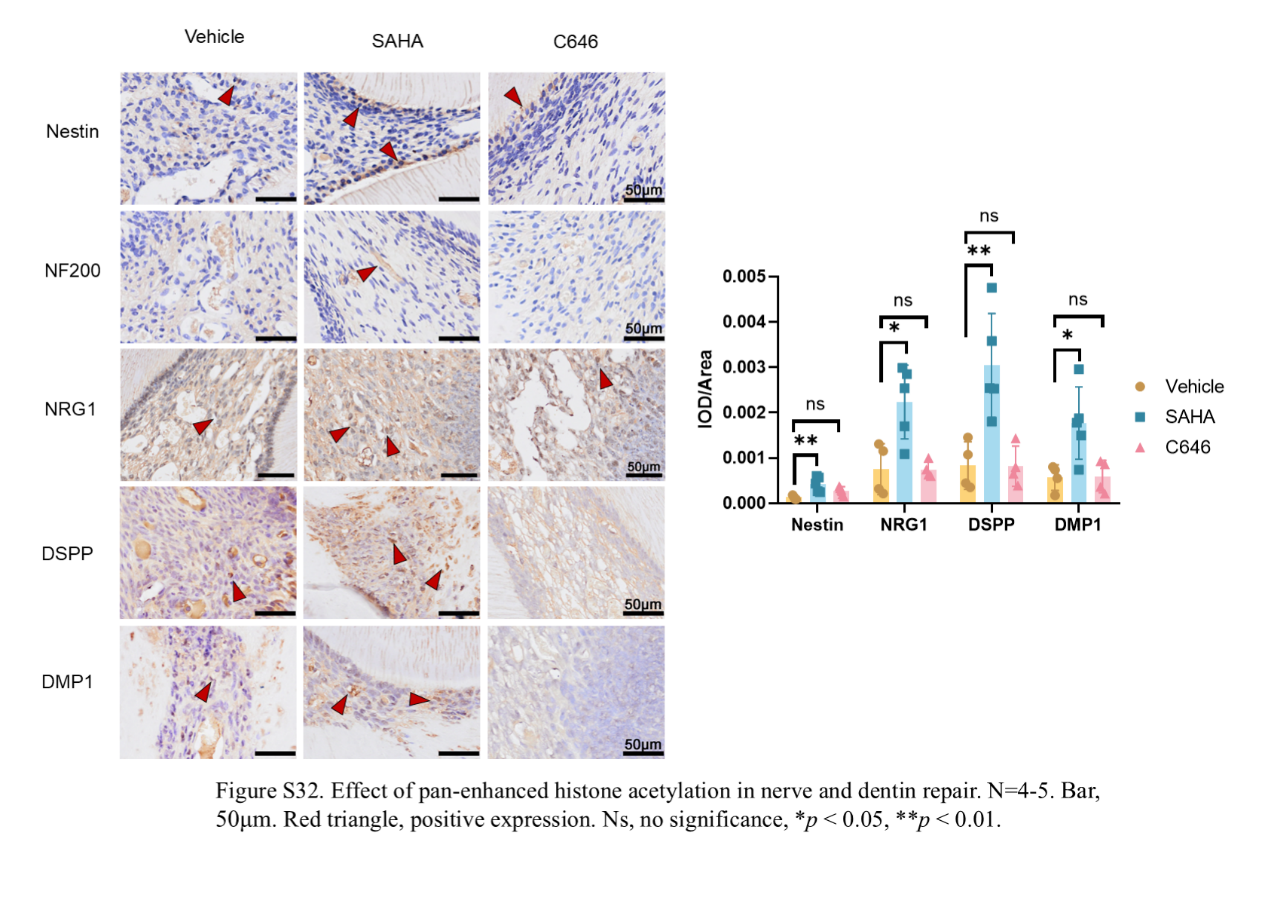


Figure S32. Effect of pan-enhanced histone acetylation in nerve and dentin repair. N=4-5. Bar, 50μm. Red triangle, positive expression. Ns, no significance, **p* < 0.05, ***p* < 0.01.

**Figure S33**


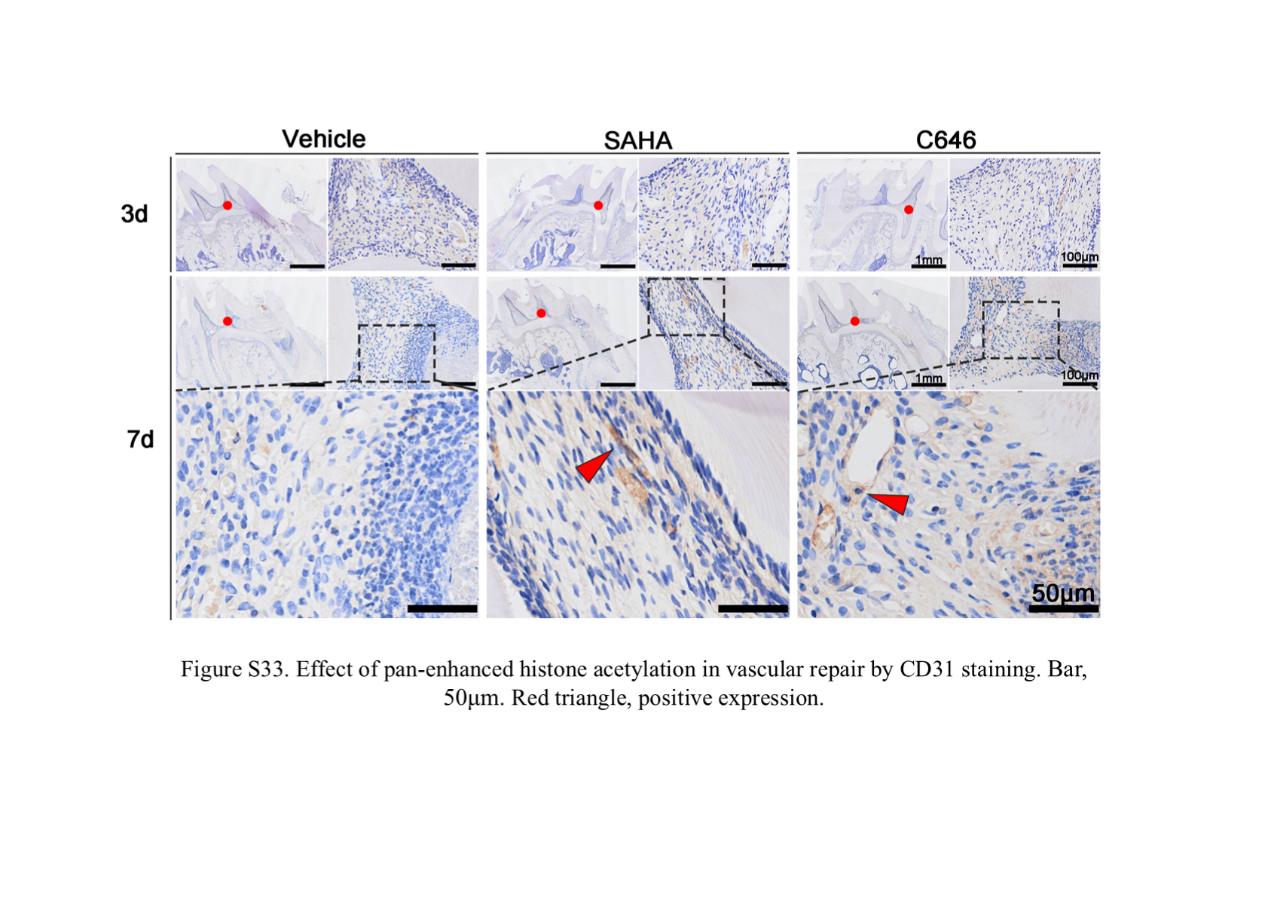


Figure S33. Effect of pan-enhanced histone acetylation in vascular repair by CD31 staining. Bar, 50μm. Red triangle, positive expression.
